# Death Receptor 5 (TNFRSF10B) Is Upregulated and TRAIL Resistance Is Reversed in Hypoxia and Normoxia in Colorectal Cancer Cell Lines after Treatment with Skyrin, the Active Metabolite of *Hypericum* spp.

**DOI:** 10.3390/cancers13071646

**Published:** 2021-04-01

**Authors:** Marián Babinčák, Rastislav Jendželovský, Ján Košuth, Martin Majerník, Jana Vargová, Kamil Mikulášek, Zbyněk Zdráhal, Peter Fedoročko

**Affiliations:** 1 Institute of Biology and Ecology, Faculty of Science, Pavol Jozef Šafárik University in Košice, Šrobárova 2, 041 54 Košice, Slovakia; marian.babincak@upjs.sk (M.B.); rastislav.jendzelovsky@upjs.sk (R.J.); jan.kosuth@upjs.sk (J.K.); martin.majernik@upjs.sk (M.M.); jana.vargova@upjs.sk (J.V.); 2 Central European Institute of Technology, Masaryk University, Kamenice 5, 625 00 Brno, Czech Republic; 357352@mail.muni.cz (K.M.); zdrahal@sci.muni.cz (Z.Z.); 3 National Centre for Biomolecular Research, Faculty of Science, Masaryk University, Kamenice 5, 625 00 Brno, Czech Republic

**Keywords:** skyrin, hypoxia, colorectal cancer, proteomics, Death receptor 5, TRAIL, TRAIL resistance, *Hypericum*

## Abstract

**Simple Summary:**

Hypoxic conditions are common in solid tumors and are very often connected with the poor prognosis of cancer patients. The lack of oxygen often causes the failure of therapy due to multiple mechanisms increasing cancer survival. Skyrin (SKR) is a secondary plant metabolite from the genus *Hypericum* spp., with a potential anticancer effects but still unknown mechanism of action. Based on our comprehensive analysis of SKR action, SKR shows significant efficiency against cancer, but not healthy cells, induces apoptosis, and upregulates Death receptor 5 in cancer cells in normoxic, as well as hypoxic conditions. SKR reverses TRAIL resistance even in TRAIL-resistant cancer cell lines in hypoxia. To sum up, SKR can be possibly used as a natural antitumor drug *per se* or as an adjuvant to TRAIL treatment in hypoxic and therapy-resistant tumors.

**Abstract:**

Skyrin (SKR) is a plant bisanthraquinone secondary metabolite from the *Hypericum* genus with potential use in anticancer therapy. However, its effect and mechanism of action are still unknown. The negative effect of SKR on HCT 116 and HT-29 cancer cell lines in hypoxic and normoxic conditions was observed. HCT 116 cells were more responsive to SKR treatment as demonstrated by decreased metabolic activity, cellularity and accumulation of cells in the G1 phase. Moreover, an increasing number of apoptotic cells was observed after treatment with SKR. Based on the LC-MS comparative proteomic data from hypoxia and normoxia (data are available via ProteomeXchange with the identifier PXD019995), SKR significantly upregulated Death receptor 5 (DR5), which was confirmed by real-time qualitative PCR (RT-qPCR). Furthermore, multiple changes in the Tumor necrosis factor-related apoptosis-inducing ligand (TRAIL)-activated cascade were observed. Moreover, the reversion of TRAIL resistance was observed in HCT 116, HT-29 and SW620 cell lines, even in hypoxia, which was linked to the upregulation of DR5. In conclusion, our results propose the use of SKR as a prospective anticancer drug, particularly as an adjuvant to TRAIL-targeting treatment to reverse TRAIL resistance in hypoxia.

## 1. Introduction

**Figure 1:**
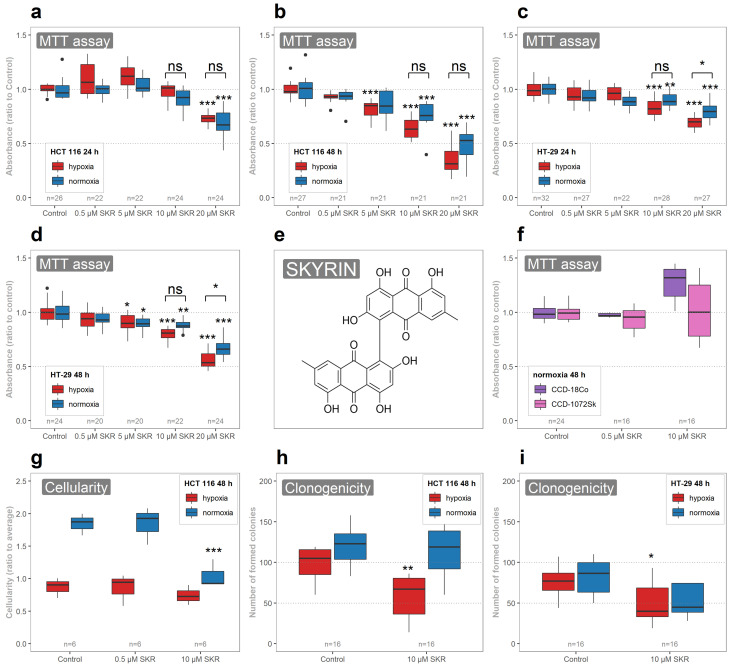
The effect of SKR on the basic cellular parameters of normal and cancer cell lines. (**a**–**e**) SKR significantly decreases the metabolic activity of both cancer cell lines, with the most prominent effect in HCT 116 cells, but with no effect on healthy cells. (**a**,**b**) In cancer cell line HCT 116, there was a significant effect after treatment with SKR, as observed in the 5 μM concentration of SKR in hypoxic conditions and 10 μM concentration of SKR in normoxic conditions after 48 h. (**c**,**d**) In HT-29 cells, the effect of SKR on metabolic activity was significant in higher concentrations, as observed in HCT 116 cell line. (**e**) Molecule of skyrin (SKR). (**f**) SKR has no negative effect on the metabolic activity of pathologically unchanged cell lines in used concentrations. (**g**) The relative cellularity in treated samples of HCT 116 cell line in normoxia was significantly decreased, approximately to the levels of untreated cells in hypoxia. There were no significant changes in the cellularity of HCT 116 cell line after the treatment with SKR in hypoxia. (**h**,**i**) The clonogenicity of SKR treated cells was not increased towards the untreated samples. The results are expressed as boxplots of at least three independent experiments. The experimental groups were compared with the matching control group in the same condition (e.g., hypoxic control or normoxic control). The number of samples (n) is noted in plots. Comparison between hypoxia and normoxia samples is denoted over the compared samples (* *p* < 0.05, ** *p* < 0.01, *** *p* < 0.001), ns—not significant).

Skyrin (SKR; CAS No. 602-06-2; PubChem CID: 73071; C${}_{30}$H${}_{18}$O${}_{10}$; molecular weight: 538.5 g/mol) is a plant secondary metabolite that is structurally very similar to hypericin. It is probably involved in hypericin synthesis [1,2] or a molecule formed in parallel reactions [3]. It was first isolated from *Penicillium islandicum* in 1954 by Howard and Raistrick [4]. Later, it was isolated from many other fungi, lichens and plants [1,5,6,7,8,9,10,11,12,13,14,15,16,17]. It has a conformation of an emodin homodimer with a bisanthraquinone structure (Figure 1e) [5,13,14,18].

The biological activity of SKR has been studied at several levels, from the inhibition of viral enzymes [6], through antimicrobial activity against Gram-negative and Gram-positive bacteria [19,20] to cytotoxic activity against several cell lines [8,11,12,16,21]. However, the mechanism of action of SKR is not fully understood. Kiyoshi et al. attributed the cytotoxic activity of SKR on mouse lymphocytic leukemia cell line L1210 to impaired adenosine triphosphate (ATP) synthesis in mitochondria [5]. There was also observed DNA fragmentation with higher concentrations of SKR in the HL-60 cell line [8]. SKR also interacts with casein kinase, estrogen receptor, dopamine beta-hydroxylase and glucagon receptor [22]. Moreover, ${}^{131}$I labeled SKR showed selective accumulation in necrotic tissue comparable to hypericin that makes it a suitable candidate as a radioisotopic drug in the therapy of solid tumors [21]. This feature of SKR could be particularly interesting regarding solid tumors, which often possess a necrotic core that is closely related to the hypoxic area. Thus, SKR could preferentially accumulate and affect hypoxic cells that are related to the chemoresistant and invasive phenotype. Many plant secondary metabolites from the *Hypericum* genus are used in the treatment of different types of cancer, although they are related to various side effects, as reviewed in [23,24].

Based on this, the interaction of SKR with biomolecules in hypoxic tissue, as well as the effect of SKR alone on those tumor cells that adapted to reduced oxygen, could be potentially interesting. Hypoxia is a condition in which a reduced oxygen content below a critical value is associated with the reduced function of organs and tissues [25,26,27]. In solid tumors, hypoxic conditions occur for several reasons, in particular due to the increased distance of the cells from the oxygen source, to structural abnormalities of the tumor vasculature or to decreased microcirculation within a solid tumor [25,26,28].

From the molecular point of view, cells in hypoxia undergo significant changes. The main reason is the elevated activity of the HIF-1 (hypoxia-inducible factor 1) complex as a result of the coupling of oxygen-sensitive subunit HIF-1α with HIF-1β [29,30]. HIF-1 regulates the expression of many genes involved in cell survival [26,28,31,32]. In cancer, HIF-1 activity is connected to angiogenesis, cell proliferation, metabolic adaptation or invasion and metastasis [33]. Many other processes are also regulated by HIF transcription factors, especially those related to drug resistance or treatment failure [33,34,35].

Death receptor 5 (DR5), known as Tumor necrosis factor-related apoptosis-inducing ligand (TRAIL) receptor 2 or Tumor necrosis factor receptor superfamily 10B or CD262 (DR5, TRAILR2 or TNFRSF10B; UniProt reference O14763) is a surface receptor that binds TRAIL and mediates apoptosis [36,37,38,39]. Moreover, HIF-2α can act as a promoter of DR5 expression [40]. However, TRAIL-promoted apoptosis is attenuated in hypoxia [41,42]. Many drugs with a similar chemical structure to SKR upregulate DR5 [43,44,45,46,47], but the mechanism is still unknown. In our study, we chose the colorectal cancer cell line, which represents a model of rapidly expanding cancer cells forming hypoxic central regions within the solid mass [48,49]. Hypoxia is also coupled with a poor prognosis to TRAIL-mediated treatment in cancer patients [41,42,50]. The lack of oxygen in combination with the increase of anti-apoptotic Bcl-2 (B-cell lymphoma 2 protein) attenuates TRAIL-mediated therapy in hypoxic conditions [41,42,51]. Thus, the sensitization of hypoxic tumors to TRAIL-mediated treatment represents a propsective anticancer strategy.

## 2. Results

### 2.1. SKR Selectively Decreases the Metabolic Activity of Cancer Cells and Their Clonogenic Ability in Hypoxia

Our first aim was to determine the effect of SKR on the metabolic activity of cancer cell lines and to determine the effective concentration for further experiments to observe a moderate effect of SKR. We observed a significant decrease in metabolic activity in both cancer cell lines treated with SKR, even after the shorter incubation period (24 h) and in both cultivation conditions based on the decreased amount of formed formazan from the MTT assay. Calculated concentrations causing a 25% decrease of metabolic activity based on the MTT assay are shown in Table 1.

However, we observed some differences in the SKR effect on the metabolic status of cells, as follows. The effect was more prominent (1) after a longer exposure of cells to SKR (24 vs. 48 h), (2) in hypoxic compared to normoxic conditions and (3) in HCT 116 cell line compared to HT-29 (Figure 1a–d). Based on the results, the concentration of SKR for further experiments was set to 10 μM.

To assess the effect of SKR on healthy cells, human fibroblast cells from the colon (CCD18-Co) and foreskin (CCD-1072Sk) cultivated in normoxic conditions were used. In contrast to cancer cells, there was no decrease of metabolic activity after SKR treatment in CCD18-Co and CCD-1072Sk cell lines, not even after prolonged exposure (48 h; Figure 1f).

After this, we intended to determine the possible effect of SKR on the total cellularity (the total number of cells) and size of cells. There were no significant changes in the size of cells based on treatment, oxygen content or cultivation period (14.1 ± 0.57 μm in HCT 116 and 15.3 ± 0.80 μm in HT-29, Figure S1). The inhibitory effect of hypoxia on cellularity was observed in both cell lines after 48 h; in cell line HCT 116, there was also a minor effect after 24 h. The significant inhibitory effect of SKR on cellularity was noticed only in HCT 116 cells in normoxia after prolonged treatment. In hypoxic conditions, no changes were observed between treated and untreated samples (Figure 1g and Figure S2). To finalize the complete picture of SKR effects on the basic parameters of cancer cells, the measurement of clonogenic ability was performed. As shown in Figure 1h,i, SKR does not affect the capability of cancer cells to form colonies under normoxia. However, when applied in hypoxia, a significant decrease in the total number of formed colonies was observed in both cancer cell lines (Figure 1i).

### 2.2. SKR Increases the Accumulation of Cells in G1 Phase and Induces Apoptosis

To look deeper into the potential mechanism action of SKR, we performed flow cytometry analysis to detect the potential incidence of apoptosis and necrosis in cells after treatment with SKR.

As shown in Figure 2i,j, SKR significantly increased the number of apoptotic cells in HCT 116 cell line after 48 h simultaneously with a decreasing number of live cells. Moreover, this effect was more remarkable in hypoxia than in normoxia. Furthermore, a smaller, nonsignificant increase in the number of apoptotic cells was observed in HT-29 cells treated for 48 h in hypoxia (Figure S3d). Representative FACS plots are shown in Figure 2a–h, and primary FACS data with backgating are presented in the Supplementary Materials (Figures S6–S29).

Finally, to determine the potential effect of SKR on the cell cycle distribution in cancer cells, the samples were stained with PI, and the amount of DNA was evaluated by flow cytometry. Significant changes in the distribution of individual phases of the cell cycle were observed only in HCT 116 cell line. After treatment with SKR under hypoxia, there was an increased number of cells in the G1 phase after 24 h (35.80% ± 2.29% compared to 28.28% ± 2.31% in hypoxic control). This effect was more pronounced after prolonged exposure to SKR (48 h) not only in hypoxic (from 58.96% ± 0.98% in control to 67.02% ± 1.38% in 10 μM SKR samples) but also under normoxic conditions (from 59.35% ± 4.65% in control to 64.87% ± 2.19% in 10 μM SKR samples) (Figure 3 and Figure S4).

### 2.3. SKR Upregulates Pathways Associated with Mitophagy and TRAIL Signalization

Since there is little known about the molecular basis of the action of SKR on cells in general, we employed comparative proteomics to reveal changes in the proteome. Due to the different actions of SKR on both employed cancer cell lines, the more sensitive HCT 116 cells and a 10 μM concentration of SKR with a more powerful effect were used. The whole proteome was measured and analyzed as described in Section 4. In total, 4974 protein groups containing 50,783 peptides were successfully identified from 1,128,203 MS/MS spectra of trypsin-digested samples. Based on the fact that all compared samples were collected at the same time after comparable treatment, Pearson’s correlation coefficients were higher than 0.94 in all pairs (Figure 4a), but with notably higher correlations within the samples from hypoxia than from normoxia. The protein group intensities were normalized, and Log2FC values between groups of samples for each protein group were calculated. For the threshold for the rating of the protein group to be significant, the adjusted *p* value for the difference between treated and untreated samples must be lower than 0.05 simultaneously in both hypoxia and normoxia. The threshold for changed regulation was set as Log2FC > +1.0 for upregulation and Log2FC < −1.0 for downregulation simultaneously or +1.5 and −1.5 for particular analysis (Figure 4b,c).

We identified 508 significantly changed protein groups in hypoxia, 801 protein groups in normoxia and 158 significantly changed protein groups in both conditions simultaneously. The numbers of upregulated protein groups after SKR treatment were 66 and 61 for hypoxia and normoxia, respectively, and the numbers of downregulated protein groups after SKR treatment were 82 and 72 for hypoxia and normoxia, respectively. In both conditions simultaneously, there were seven upregulated and eight downregulated protein groups with a Log2FC level over +1.5 or below −1.5. A list of upregulated and downregulated protein groups with Log2FC and relative abundancy is shown in Figure 4d. In Table 2, the list of all significantly upregulated and downregulated proteins is presented. The list of all identified protein groups with intensities and Log2FC within samples is provided in List of all identified protein groups (Data S1) in the Supplementary Materials.

After this, the analysis of affected pathways was performed using Reactome. Lists of significantly affected pathways are in Table 3 and Table 4. Most of the significantly overrepresented pathways with upregulated proteins were coupled with two main processes: (1) the regulation of cell death and survival connected to TRAIL signaling or extrinsic apoptosis signaling via death receptors and (2) the degradation process of mitochondria. The results from the Reactome pathway analysis of significantly downregulated protein groups might be linked to (1) the downregulation of PTEN regulating proteins leading to the reduction of cell proliferation and apoptosis promotion and (2) pathways connected to the apoptotic process in the cell.

### 2.4. SKR Significantly Upregulates Death Receptor 5 in HCT 116 Cells in Hypoxia and Normoxia

Based on the results of the comparative proteomic analysis of HCT 116 cell line treated with SKR in both conditions (normoxia and hypoxia), we assumed that the potential mechanism of SKR action could be based on upregulation of a protein known as Death receptor 5 (DR5; TNFRSF10B; UniProt reference O14763) encoded by the Tumor necrosis factor receptor superfamily 10B gene (*TNFRSF10B*). The level of protein was significantly upregulated in HCT 116 cells treated by SKR regardless of cell oxygenation (Figure 5a).

We also focused on the expression of other TRAIL and TNF-relevant proteins. Another TRAIL receptor–Death receptor 4 (DR4; TNFRSF10A; UniProt reference O00220) encoded by the Tumor necrosis factor receptor superfamily 10A gene (*TNFRSF10A*)–was significantly changed in hypoxia but not upregulated (Log2FC in hypoxia was 1.38, threshold for upregulation was +1.5; Figure 5b). In the expression of decoy receptor 3 (DcR3; TNFRSF6B; UniProt reference O95407) or FAS-associated death domain protein (FADD; UniProt reference Q13158), no significant changes were observed (Figure 5c,d). Neither TNF alpha, TNFR1 nor TNFR2 were identified by MS.

To determine if this upregulation of the DR5 protein by SKR was the result of the increased level of gene mRNA or solely protein stabilization (or some other posttranscriptional/posttranslational mechanism), RT-qPCR was performed. Consistent with proteomic analysis, we analyzed the expression of mRNA after 48 h incubation with SKR (in HCT 116 and HT-29 cell lines). We also considered the gene expression after 2 and 6 h to observe the kinetics of mRNA synthesis (TRAIL-resistant cell lines, HT-29 and SW620 cell lines, were employed for this purpose). The results presented in Figure 6a,d show that in HCT 116 cell line, regardless of oxygen level, SKR significantly increased the level of the *TNFRSF10B* gene transcripts represented by the both alternatively spliced isoforms, the isoform 1 (Figure 6a) and the isoform 2 (Figure 6d), as shown by the gene expression after 48 h. In HT-29 cell line, there was a significant increase in the *TNFRSF10B* mRNA level (in the both isoforms) after 48 h only in normoxia (Figure 6b,e). On the contrary, incubation with SKR after 6 h showed the increased expression of *TNFRSF10B* (in both isoforms) in hypoxia (Figure 6c,f). A similar effect on *TNFRSF10B* gene expression after 6 h was found also in SW620 cell line (see Figure S5e; both isoforms were detected simultaneously).

To complete the results from the analysis of the relevant proteins (detected by MS), an analysis of mRNAs encoding other TRAIL receptors and TNF-relevant proteins, namely DR4 (encoded by *TNFRSF10A* gene), TNF alpha (*TNF*), TNFR1 (*TNFRSF1A*), TNFR2 (*TNFRSF1B*) and DcR3 (*TNFRSF6B*), was performed. We found a significantly decreased mRNA level for DR4 (*TNFRSF10A*) in hypoxia after 48 h of SKR treatment in HCT 116 cell line (Figure 6g). In HT-29 cell line, no significant difference was observed (Figure 6h,i). The mRNAs of *TNF* as well as TNFR1 (*TNFRSF1B*) were below the limit of quantification in all samples. The amount of transcript encoding the TNF receptor TNFR2 (*TNFRSF1A*) was not significantly affected by SKR treatment within all analyzed samples (Figure 6j–l). Furthermore, the detected level of DcR3 mRNA was not influenced by the SKR treatment in both lines, although in HT-29 the mRNA level of the gene was below the limit of quantification.

### 2.5. SKR Reverse TRAIL Resistance in p53 Mutated Cancer Cell Line Even in Hypoxia

The next step was to determine whether the upregulation of DR5 was transformed to increase the sensitivity of cells to TRAIL. TRAIL solutions in concentrations based on our preliminary data were added to cells 24 h before the analysis (24 h after SKR treatment). The final concentration for HCT 116 cell line (TRAIL receptive cell line; p53 wild type) was 10 ng/mL, and concentrations for HT-29 and SW620 cell lines (both are TRAIL-resistant cell lines; p53 R273H mutant cell lines) were 50 ng/mL.

TRAIL increased the number of apoptotic cells in HCT 116 cell line in both conditions; however, the effect of TRAIL in hypoxia was less prominent than in normoxia. Conversely, effects of TRAIL only on HT-29 and SW620 cell lines were observed only in hypoxia. There was no significant effect of SKR with TRAIL on the induction of apoptosis in HCT 116 cell line in comparison with SKR alone (Figure 7i). On the other hand, SKR in combination with TRAIL decreased the number of live cells (Figure 7j). In HT-29 cell line as well as in SW620 cell line, the effect of TRAIL was amplified by SKR with a different level of effectiveness in both conditions, with a more prominent effect in hypoxia (Figure 8 and Figure 9). Representative FACS plots are shown in Figure 7a–h, Figure 8a–h, Figure 9a–h, and primary FACS data with backgating are presented in the Supplementary Materials (Figures S6–S29). Therefore, we assume that SKR causes the upregulation of DR5 in TRAIL-receptive as well as TRAIL-resistant cell lines, leading to the sensitization of cells to the treatment targeting this receptor.

## 3. Discussion

To determine the effect and the molecular background of the action of SKR, different cell lines cultivated under different cultivation conditions and exposed to different treatment regimens were used in our study. To do so, we analyzed the metabolic activity of SKR-treated cells, evaluated their cellularity, colony-forming ability, cell cycle distribution and the occurrence of apoptosis and necrosis and performed comparative proteomics analysis.

In our study, two different human colorectal adenocarcinoma cell lines were used with distinct responses to SKR action and hypoxia. The main difference between these cell lines that could be manifested in their different sensitivity to SKR was that HCT 116 is a p53 wild-type cell line with defect DNA mismatch repair but without chromosomal instability [52]. In contrast, HT-29 is a p53 mutant with intact mismatch repair and chromosomal instability [52]. In our study, a more pronounced effect of SKR on HCT 116 cells was observed, which was reflected by decreased metabolic activity, an increased number of apoptotic cells and the accumulation of cells in G1. It is known that p53 knocked-out cells are more sensitive to treatment leading to DNA damage [53]. Moreover, HCT 116 cell line is more sensitive to apoptosis induced by 5-Fluorouracil and oxaliplatin because of the loss of Bax expression [54]. Based on these facts, we hypothesize that the observed effect of SKR probably does not depend on DNA damage.

The attenuated effect of SKR seen in HT-29 cell line can be associated with an overexpression of ATP-binding cassette super-family G member 2 (ABCG2) [55,56]. Elevated ABCG2 levels are responsible for decreasing the intracellular accumulation and thus the efficiency of another anthraquinone derivate—hypericin—as was shown by the decreased efficacy of the hypericin-mediated treatment of adenocarcinoma cells [56,57,58]. Many other anthraquinone derived drugs, such as mitoxantrone [59,60,61,62] or hypericin [56,57], are effluxed by ABCG2 transporter. Based on the chemical similarity of SKR to anthraquinone, we assume that SKR could be the substrate for ABCG2 transporter protein. This could explain the diminished impact of SKR on the metabolic activity of HT-29 cells (with overexpressed ABCG2) compared to HCT 116 cells (with low ABCG2 expression). Since SKR does not exhibit fluorescent properties, this needs to be further elucidated; e.g., using labeled SKR. Moreover, as our results show, HT-29 cells were not only more resistant to the effect of SKR but were also less impacted by hypoxia in general (when comparing HT-29 vs. HCT 116 but also untreated vs. treated HT-29). We assume that this could be mainly because HT-29 is more tolerant of the lack of oxygen than HCT 116 [52]. Nevertheless, the long-term cultivation in discontinued hypoxia leads to a more aggressive phenotype of HCT 116 cells [52]. Hypoxia induces several intracellular signaling pathways, such as hypoxia-inducible factor or PI3K/AKT/mTOR pathways [26].

Since we performed only an MS analysis of HCT 116 cells, we cannot confirm this assumption. However, based on a great deal of evidence from other studies, hypoxia potentiates both the level and activity of ABC transporter proteins, among which are ABCG2 and P-gp through the activation of ERK pathway [34,63,64]. Thus, the activity of efflux pumps should be significantly enhanced in hypoxia [65,66]. Despite that fact, a significant effect of SKR in hypoxia was noted in HCT 116, as well as in HT-29 with ABCG2 overexpression. Nevertheless, based on the literature, the upregulation of surface death receptors is associated with the inhibited efflux function of P-gp and downregulation of c-FLIP [67].

The dose-dependent negative effect of SKR on metabolic activity was proven in both used cancer cell lines. The effect of SKR on metabolic activity corresponds with the results of other authors. IC50 values of SKR in normoxic conditions vary from 20 μM in L1210 cells [7] to 74 μM in HL-60 cells [8] and 70 μM in A549 cells [16]; thus, its effect is obviously highly dependent on cell type. The inhibition of the metabolic activity was observed in HeLa, Vero, K592, Raji, WISH, Calu-1 and HCT 116 cells. The IC50 of SKR for HCT 116 cells after 24 h presented in the literature was set to 59 μM [11,16]. For comparison, the concentration that decreases metabolic activity to 25 % for HCT 116 cells in our study, based on an MTT assay, was set to 17.88 ± 1.56 μM in normoxia after 24 h and to 6.70 ± 0.34 μM in hypoxia after 48 h (Table 1). The theoretically calculated values for a 50% decrease of metabolic activity based on an MTT assay (only partially extrapolated from the linearized model calculated from observed data—the highest used concentration was 20 μM) are 33.27 ± 3.17 μM for 24 h in normoxia, 19.68 ± 1.54 μM for 48 h in normoxia, 38.03 ± 4.09 μM for 24 h in hypoxia and 14.84 ± 0.55μM for 48 h in hypoxia. Moreover, we performed the analysis of the metabolic activity of pathologically unchanged cells in reaction to SKR treatment. We did not notice an inhibitory effect of SKR on healthy cells (Figure 1c), leading us to assume that SKR selectively affects cancer cells. Regarding the long-term effect of SKR on cell survival, similar to the attenuated colony-forming ability seen in MIA PaCa-2 cells in normoxia [16], we noticed a significantly decreased number of formed colonies in both cancer cell lines treated with SKR in hypoxia.

**Figure 2:**
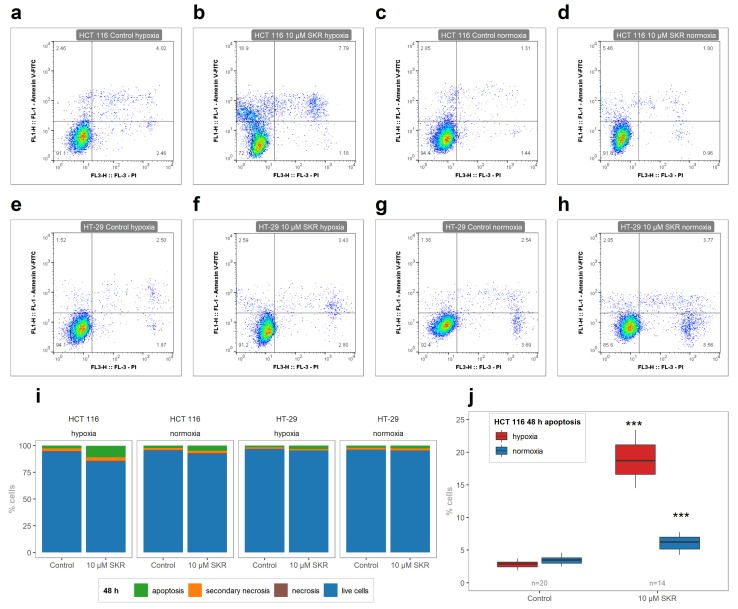
The effect of SKR on phosphatidylserine externalization in cancer cell lines HCT 116 and HT-29. (**a**–**h**) Representative FACS plots of debris-free singlets after SKR treatment in HCT 116 and HT-29 cell lines. (**i**) Ratios between the numbers of cells in gates stained with Annexin-V and PI as an average of at least three independent experiments. (**j**) Significantly increased number of apoptotic cells after treatment with SKR after 48 h in HCT 116 cell line. Control samples contain the same amount of DMSO as the sample with SKR. The results are expressed in boxplots or as average in bar plots of three independent experiments. The experimental groups were compared with the control group. The number of samples (n) is noted in plots. (*** *p* < 0.001).

**Figure 3:**
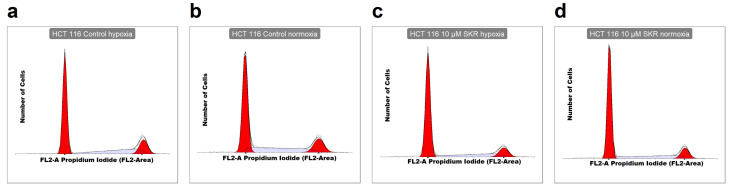

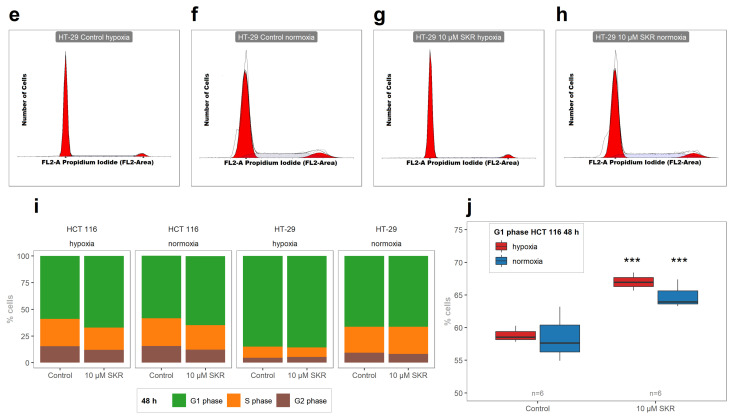
The effect of SKR on cell cycle distribution in cancer cell lines HCT 116 and HT-29. (**a**–**h**) Representative plots of cell cycle analysis after SKR treatment in HCT 116 and HT-29 cell lines. (**i**) Distributions of individual phases of the cell cycle analyzed after 48 h after treatment in HCT 116 and HT-29 cell line from at least three independent experiments. (**j**) Significant accumulation of HCT 116 cells in the G1 phase after 48 h after treatment with 10 μM SKR. Control samples contain the same amount of DMSO as the sample with SKR. The results are expressed in boxplots or as averages in bar plots of three independent experiments. The experimental groups were compared with the control group. The number of samples (n) is noted in plots. (*** *p* < 0.001).

**Figure 4:**
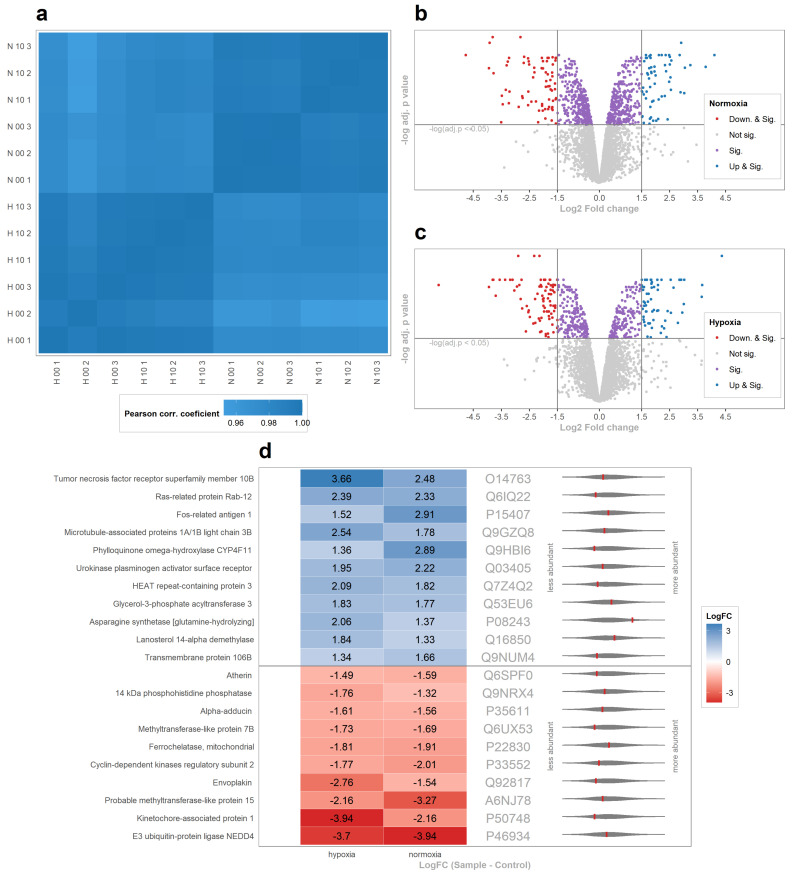
Result from the mass spectrometry (MS) analysis of the HCT 116 cells after 48 h treatment with 10 μM SKR. (**a**) Heatmap of all calculated Pearson’s correlation coefficients within all samples (hypoxia is marked by H, normoxia by N and 00 and 10 are control and 10 μM SKR samples, respectively). The last number (1–3) indicates the specific repetition. (**b**,**c**) Volcano plots of all protein groups in the samples as a comparison between control and treated samples in hypoxia and normoxia, respectively. On x axes are the Log2 Fold change values of protein groups between control and 10 μM SKR samples. The threshold for the significance of the protein group is an adj.p value lower than 0.05. (**d**) Heatmap of significantly upregulated (blue) and downregulated (red) protein groups in both conditions (hypoxia on the left and normoxia on the right) with UniProt accession numbers. Red lines in violin plots of all protein group intensity distributions represent the relative abundance of a protein group (more abundant on right).

**Figure 5:**
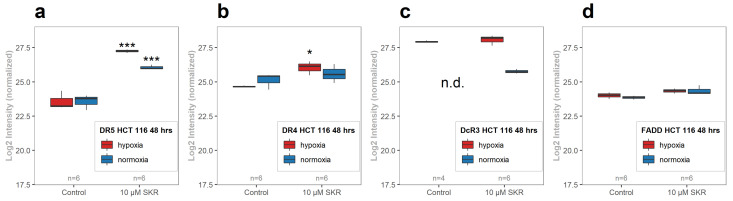
SKR after 48 h treatment of HCT 116 cells increases the expression of DR5 on protein level. Normalized log2 intensity of Death receptor 5 (DR5) protein (**a**), DR4 (**b**), DcR3 (**c**) and FAS-associated death domain protein (FADD) (**d**) evaluated by MS. The boxplots represent at least three independent experiments. DcR3 was not detected in normoxic control. The number of samples (n) is noted in plots. The experimental groups were compared with the matching control group (* *p* < 0.05, *** *p* < 0.001). n.d.— not detected.

The level of apoptosis induced by SKR treatment can also be compared with the results of other authors. MIA PaCa-2 cells were treated with SKR in a concentration of 18 μM, and a significant increase of the number of apoptotic cells was observed by fluorescent microscopy via the detection of phosphatidylserine externalization with Annexin V [16]. In our study, the significant increase of apoptotic cells was observed in a 10 μM concentration of SKR in HCT 116 cells (Figure 2). There were no changes in the number of apoptotic cells in HT-29 cell line after SKR treatment. The comparison of healthy cells without decreased metabolic activity and cancer cell lines with an increased apoptosis rate together with the upregulation of DR5 leads us to the assumption that the effect of SKR can be caused by the higher sensitivity of cells to TRAIL.

TRAIL also known as APOL2L (Apolipoprotein L2 ligand) or CD253, is a molecule that stimulates apoptosis in cancer cells but not in normal, nontransformed cells [36,37,68,69,70,71,72]. TRAIL is also an important immune effector agent regulating the growth of tumors [38]. However, the TRAIL resistance of tumor cells is a very common problem [70,72]. TRAIL resistance can be caused by a mutation in p53 [36] as well as the downregulation of DRs or upregulation of anti-apoptotic proteins [37]. A large number of drugs have been shown to overcome TRAIL resistance. The upregulation of its receptor, DR5, is the common way of sensitizing cells to treatment; for example, with doxorubicin [43,44], goniothalamin [73], emodin [45], ibuprofen [46], mitoxantrone [47], apigenin [74], Hsp90 inhibitors [75], resveratrol [76], indirubin-3-monoxime [77] or cyclopamine [78].

**Figure 6:**
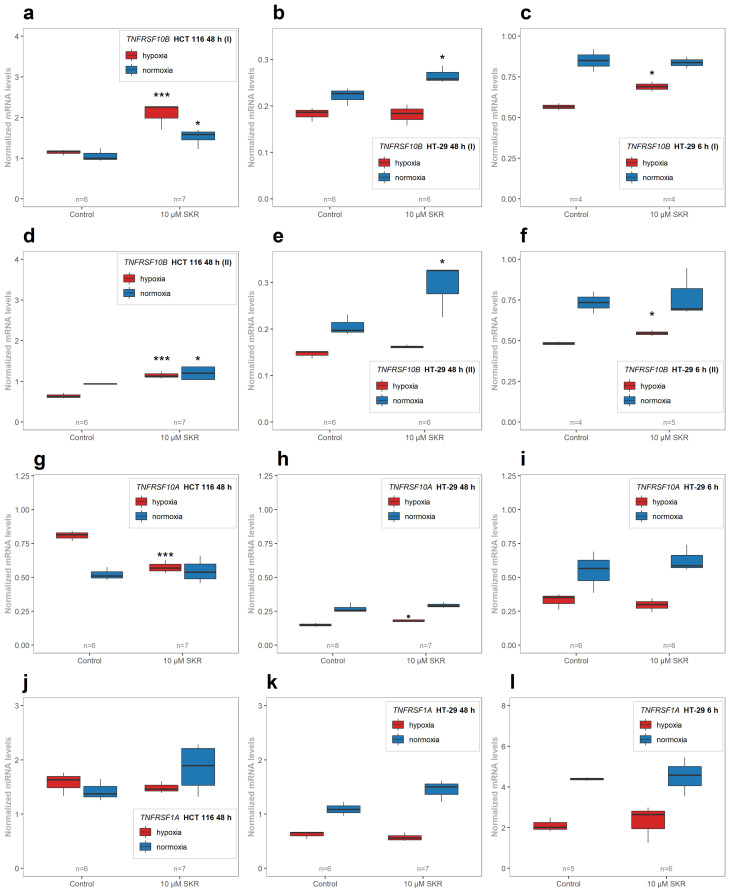
Normalized mRNA levels of *TNFRSF10B* (DR5), *TNFRSF10A* (DR4) and *TNFRSF1A* (TNFR1). Normalized mRNA levels of *TNFRSF10B* (DR5) of isoform 1 (**a**–**c**), isoform 2 (**d**–**f**) after SKR treatment. Normalized mRNA levels of *TNFRSF10A* (DR4) (**g**–**i**) and *TNFRSF1A* (TNFR1) (**j**–**l**). Normalized mRNA levels were evaluated by RT-qPCR and normalized to the level of reference gene *PMM1*. The number of samples (n) is noted in plots. The experimental groups were compared with the matching control group (* *p* < 0.05, *** *p* < 0.001).

To verify the effect of SKR on TRAIL resistance, we also performed phosphatidylserine externalization analyses of samples with TRAIL treatment and a combination of SKR and TRAIL. HT-29 cell line is TRAIL-resistant, which correlates with a notably lower basal expression of DR5 as well as DR4 (Figure 6b,c,e,f,h,i). In samples with TRAIL only, the effect in HT-29 was more pronounced in hypoxia, which was opposite to HCT 116 cell line, with a more prominent effect in normoxia. The effect of many TRAIL-targeted therapies is attenuated in hypoxia [74,78]. Furthermore, HCT 116 cells exposed to hypoxia are less susceptible to TRAIL than cells in normoxia, mainly because of the increase of anti-apoptotic Bcl-2 [41,42] and because of the attenuated apoptosis signal due to mitochondria damage during oxygen deprivation [51]. However, TRAIL-induced apoptosis can bypass the intrinsic apoptosis pathway controlled by Bcl-2 in cancer [79].

**Figure 7:**
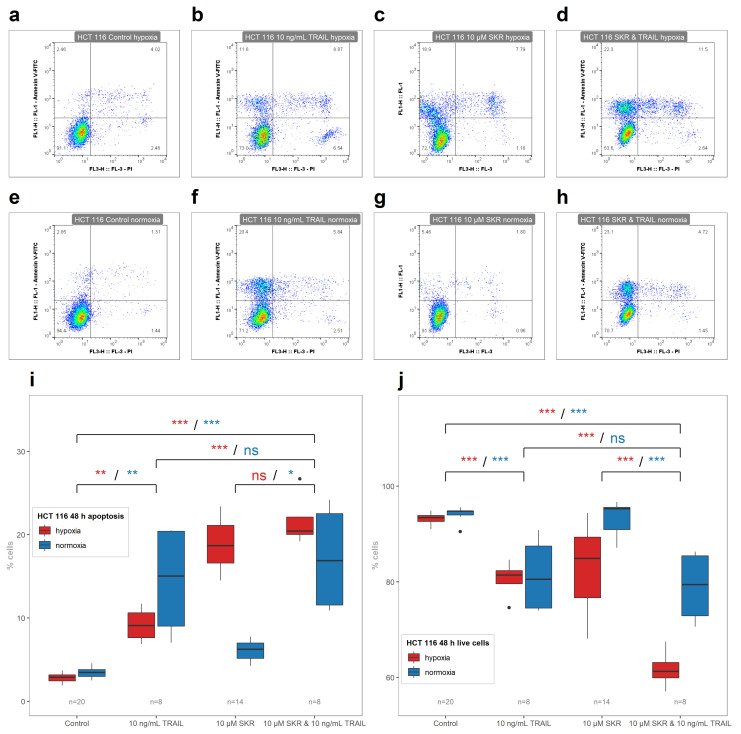
The effect of SKR in combination with TRAIL on the onset of apoptotic and live cells in HCT 116 cell line. (**a**–**h**) Representative FACS plots of debris-free singlets after SKR and TRAIL treatment in HCT 116 cell line. (**i**) Percentage of apoptotic cells after treatment with SKR and TRAIL after 48 h in HCT 116 cell line. (**j**) Percentage of live cells after treatment with SKR and TRAIL after 48 h in HCT 116 cell line. The experimental groups were compared with the matching control group or group, indicated with the line below marks. The color of the marks matches the cultivation conditions (hypoxia or normoxia). The number of samples (n) is noted in the plots. (* *p* < 0.05, ** *p* < 0.01, *** *p* < 0.001, ns—not significant).

TRAIL in combination with SKR has a cooperative effect in hypoxia on HCT 116 cell line based on the decreased number of live cells (Figure 7j). In normoxia, the effect of SKR and TRAIL was on the level of TRAIL. We assume that the lack of an increase of apoptotic cells can be caused by the relative overabundance of DR5, as well as in HCT 116 cells without SKR pretreatment, where the amount of naturally present TRAIL in the medium is sufficient for apoptosis to be switched on.

**Figure 8:**
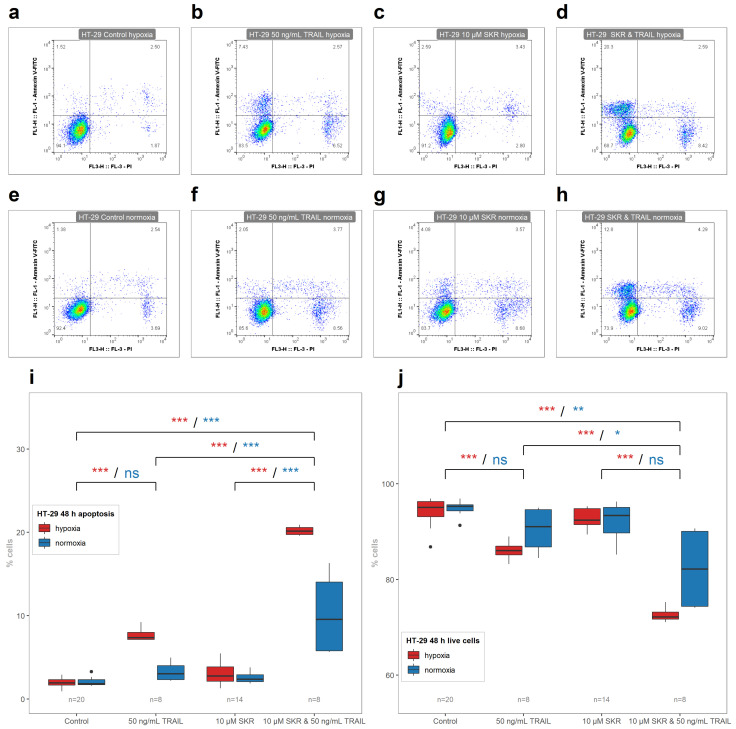
The effect of SKR in combination with TRAIL on the onset of apoptotic and live cells in HT-29 cell line. (**a**–**h**) Representative FACS plots of debris-free singlets after SKR and TRAIL treatment in HT-29 cell line. (**i**) Percentage of apoptotic cells after treatment with SKR and TRAIL after 48 h in HT-29 cell line. (**j**) Percentage of live cells after treatment with SKR and TRAIL after 48 h in HT-29 cell line. The experimental groups were compared with the matching control group or group, indicated with a line below marks. The color of the marks matches the cultivation conditions (hypoxia or normoxia). The number of samples (n) is noted in the plots. (* *p* < 0.05, ** *p* < 0.01, *** *p* < 0.001, ns—not significant).

**Figure 9:**
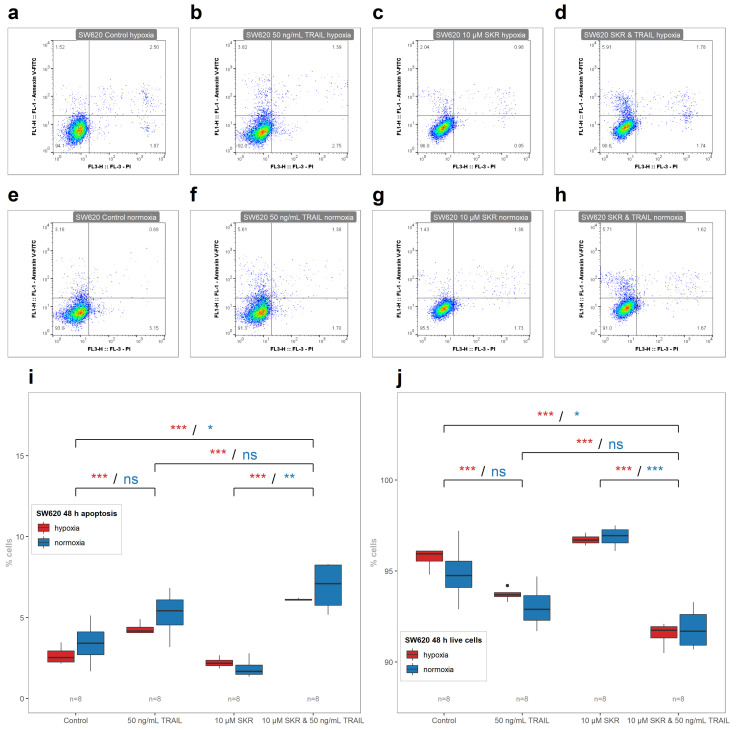
The effect of SKR in combination with TRAIL on the onset of apoptotic and live cells in SW620 cell line. (**a**–**h**) Representative FACS plots of debris-free singlets after SKR and TRAIL treatment in SW620 cell line. (**i**) Percentage of apoptotic cells after treatment with SKR and TRAIL after 48 h in SW620 cell line. (**j**) Percentage of live cells after treatment with SKR and TRAIL after 48 h in SW620 cell line. The experimental groups were compared with the matching control group or group, indicated with a line below marks. The color of the marks matches the cultivation conditions (hypoxia or normoxia). The number of samples (n) is noted in the plots. (* *p* < 0.05, ** *p* < 0.01, *** *p* < 0.001, ns—not significant).

There was also an enhanced effect of TRAIL on apoptosis induction in SKR-pretreated cells in HT-29 cell line in hypoxia as well in normoxia (Figure 8). In SW620 cell line, the only noted effect was that the hypoxia-based TRAIL resistance was reversed; in normoxia, only a non-significant increase was observed (Figure 9). In both resistant cancer cell lines, the upregulation of DR5 was reported. In HT-29, there was a significant increase in both isoforms of *TNFRSF10B* with different mRNA level kinetics. After 6 h, the upregulation was observed in hypoxia, without a change in normoxia, as in the SW620 cell line. In normoxia, the upregulation of DR5 on the mRNA level was observed in HT-29 in both isoforms after 48 h but without the changes in normoxia. The upregulation of death receptors (DR4 or DR5) induces apoptosis [73,80]. On the other hand, we assume that the lack of an effect of a single SKR treatment on the apoptosis of HT-29 and SW620 can be caused by an insufficient amount of naturally present TRAIL ligand [81] in cultivation media for the induction of apoptosis; i.e., an amount below the apoptosis-inducing threshold.

In TRAIL-resistant cancer cell lines (HT-29 and SW620), the effect of SKR and TRAIL in combination was significantly cooperative. The upregulation of the receptor can be connected to the higher sensitivity of the cell to the ligand; in this case, TRAIL, which results in the induction of apoptosis through caspase-8 activity and Bid cleavage [82]. We also confirmed the upregulated levels of *TNFRSF10B* mRNA in all cell lines after SKR treatment.

To complete the overall view of the effect of SKR on cells, the RT-qPCR analysis of other TNF-related genes was performed. *TNF* (TNF alpha) as well as *TNFRSF1B* (TNFR2) were below the limit of quantification, which corresponded with the available data from the databases. The absence of significant changes in normalized mRNA levels of *TNFRSF1A* (TNFR1) and *TNFRSF6B* (DcR3) leads us to the assumption that the effect of SKR is not mediated by TNF alpha.

For a confirmation of TRAIL pathway activation in cells after treatment with SKR, we compared the expected proteomic changes with observed changes acquired from the quantitative mass spectrometry data. According to the literature, the TRAIL signaling pathway is triggered by the binding of a ligand to DR5 and results in a cascade of reactions. For example, the nuclear factor kappa B (NF-κB) pathway is downregulated, which is connected to the decrease of cell survival and activation of genes involved in immune reaction and inflammation. The key regulatory protein of this cascade is NF-κB essential modulator (NEMO), which is upregulated as a result of TRAIL pathway activation [83,84]. In our study, NEMO, the regulatory subunit of the inhibitor IκB kinase complex, which activates the NF-κB pathway, was significantly upregulated after the treatment with SKR in hypoxia (Log2FC = 1.45). In normoxia, we observed only a nonsignificant increase of NEMO. The difference between hypoxia and normoxia can be also demonstrated by the comparison of control samples, where NEMO is significantly downregulated in hypoxia compared to normoxia. Additionally, the inhibition of the PI3K pathway sensitized tumor cells to TRAIL treatment [85]. The deactivation of PI3K leads to the deactivation of mTOR and further deregulation of p70 protein. In our study, the p70 was not significantly reduced after the treatment with SKR. Moreover, the BIRC6 protein, known as Livin, was downregulated in hypoxia (Log2FC = −0.94) as well as normoxia (Log2FC = −0.43). The downregulation of BIRC6 is related to TRAIL pathway activation [86]. Likewise, NEDD4 regulates the tumor-suppressor PTEN by ubiquitin-mediated proteasomal degradation [70,87,88]. Thus, the downregulation of NEDD4 by SKR in hypoxia and normoxia (Log2FC = −3.70 and −3.94) could be responsible for decreased PTEN degradation, leading to the impedence of cell growth and migration and promotion of apoptosis. TRAIL signalization in hypoxia partially leads to necrosis at the expense of apoptosis. This mechanism is controlled by low extracellular pH [89]. The key proteins are RIPK1 and PARP-1. Their inhibition is connected to decreased necrosis due to the switched TRAIL pathway [84,89,90]. In our samples, there was no change in the regulation of RIPK1 in normoxia after SKR treatment; in hypoxia alone, this protein was not significantly upregulated.

The analysis of the metabolic activity, cellularity and colony-forming ability of cells after SKR treatment was used to determine the overall effect of SKR on cells. HCT 116 cell line, which expresses only a low level of the potential SKR transporter ABCG2 and is less tolerant to hypoxia, showed higher sensitivity to SKR treatment. This was demonstrated by a decreased metabolic activity, cellularity and accumulation of cells in the G1 phase. We did not observe the effect of SKR on healthy cells. Moreover, the increased number of apoptotic cells after treatment with SKR in HCT 116 cell line correlated with the observations from MS. These data indicate that the potential mechanism of SKR action can be mediated by the upregulation of DR5. This was also confirmed by RTq-PCR and changes in downstream of TRAIL-activated cascade after DR5 stimulation. SKR also reverses TRAIL resistance in HT-29 cell line in hypoxia and normoxia as well as eliminating the lack of an effect of TRAIL to HCT 116 and SW620 cells in hypoxia.

## 4. Materials and Methods

### 4.1. Reagents

SKR (CAS no: 602-06-2, Sigma-Aldrich, St. Louis, MO, USA) stock solution (c = 5 mM) was prepared in dimethylsulfoxide (DMSO) and further diluted to freshly prepared working solutions immediately before addition to cell cultures. In experiments, the control sample with the same concentration of DMSO as the sample with 10 μM was used to establish the effect of the carrier. Recombinant TRAIL protein (Abcam, Cambridge, UK; ab9960) stock solution (0.5 mg/mL) was prepared by the reconstitution of lyophilized powder in pure water and further diluted to freshly prepared working solutions immediately before use.

### 4.2. Cell Cultures

HT-29, HCT 116, SW620, CCD-18Co and CCD-1072Sk cell cultures were purchased from American Type Culture Collection (ATCC, Rockville, MD, USA). HT-29 and SW620 cells were cultured in complete RPMI-1640 medium (Sigma-Aldrich, St. Louis, MO, USA), HCT 116 cells in McCoy’s medium (PAN-Biotech GmbH, Aidenbach, Germany), CCD-18Co and CCD-1072Sk in MEM medium (Biosera, Nuaille, France). All cultivation media were supplemented with 10% fetal bovine serum (Biosera, Nuaille, France) and antibiotics (1% antibiotic-antimycotic 100 × and 50 μg/mL gentamicin; Biosera, Nuaille, France).

### 4.3. Cultivation Conditions and Experimental Design

To compare the effect of SKR on cancer cells under different oxygen concentrations, cells were cultivated in a glove box under controlled conditions (Coy Laboratory Products, Inc., Grass Lake, MI, USA) with two separate cabinets. The oxygen concentration in the hypoxic cabinet was set to 1% (partially 1% of O${}_{2}$, 5% of CO${}_{2}$, 94% of N${}_{2}$) and in the normoxic cabinet to 20% (partially 20% of O${}_{2}$, 5% of CO${}_{2}$, 75% of N${}_{2}$). Humidity and temperature were the same for both cabinets; i.e., 95% and 37 ${}^{\circ }C$, respectively.

After seeding, cells were allowed to settle for 8 h at standard conditions in an incubator. Subsequently, cells were moved to hypoxic or normoxic chambers and cultivated for 16 h. Then, SKR solutions (from 0.5 μM to 20 μM) were added to cells for 24 or 48 h with subsequent analysis. All experiments (except the mass spectrometry analysis, RT-qPCR analysis and phosphatidylserine externalization analysis in combination with TRAIL) were performed at least in three independent technical and biological replicates for all measured factors: (1) cell lines: HT-29 and HCT 116; (2) oxygen content: 1% and 20%; and (3) time of cultivation: 24 and 48 h.

### 4.4. Metabolic Activity Assay

Metabolic activity assays (MTT) were performed as reported previously [49] to evaluate changes in the metabolic activity of cells after treatment with SKR. Solutions of SKR (from 0.5 μM to 20 μM) were added to cells in a 96-well plate (TPP Techno Plastic Products AG, Trasadingen, Switzerland) to examine the SKR effect on metabolic activity based on MTT assay and to determine SKR concentrations for further experiments. Of note, IC50 values can be only extrapolated, because of the relatively low inhibition rate of SKR after the shorter incubation period. After 24 and 48 h, MTT (3-[4,5-dimethylthiazol-2-yl]-2,5-diphenyltetrazolium bromide) (Sigma-Aldrich, St. Louis, MO, USA) from a stock solution (5 mg/mL) was added to the cells in a 96-well plate. The reaction was stopped after 4 h incubation and the insoluble formazan was dissolved by the addition of SDS at a final concentration of 3.3%. The absorbance of metabolized formazan (λ = 584 nm) was measured using a BMG FLUOstar Optima (BMG Labtech GmbH, Offenburg, Germany). Results were evaluated as the ratio of the absorbance of the treated sample to the untreated matching control. The experimental groups were compared with the matching control groups (normoxic control or hypoxic control).

### 4.5. Analysis of Cellularity and Cell Size

The analysis of cellularity (the total number of cells in the sample) and cell size was performed using the Vi-CELL XR Cell Viability Analyzer (Beckman Coulter, Indianapolis, IN, USA). Floating and adherent cells were harvested for analysis by trypsinization at scheduled time points, washed with PBS and analyzed. Cellularity is presented as the ratio of total cell numbers to the average total number of cells from all samples of the experiment. Cell size was evaluated automatically as the average diameter of cells in μm in the analyzed cell suspension. The experimental groups were compared with the matching control groups (normoxic control or hypoxic control).

### 4.6. Phosphatidylserine Externalization Analysis

For the measurement of the viability and phosphatidylserine externalization as a marker of apoptosis, a BD Pharmingen FITC Annexin V Apoptosis Detection Kit I (BD Biosciences, San Jose, CA, USA) was used according to the manufacturer’s instructions. Total cells were harvested 24 and 48 h after SKR addition. Cells were washed with PBS, centrifuged and stained with Annexin V-FITC for 20 min at room temperature in the dark and subsequently stained with propidium iodide (PI) for 5 min before the measurement. A total number of 10,000 cells per sample were analyzed using a BD FACSCalibur flow cytometer (BD Biosciences, San Jose, CA, USA). Fluorescence was detected via a 530/30 nm band-pass filter (FL-1; Annexin V—FITC) and a 670 nm long-pass filter (FL-3; PI). Raw files were analyzed using FlowJo software (Tree Star Inc., Ashland, OR, USA). Only debris-free single cells were analyzed. Examples of gating strategies are presented in the Supplementary Materials (Figures S6–S29). An apoptotic cell was considered as Annexin V${}^{+}$/PI${}^{-}$, a secondary necrotic cell was Annexin V${}^{+}$/PI${}^{+}$, a necrotic cell was an Annexin V${}^{-}$/PI${}^{+}$ cell and a live cell was Annexin V${}^{-}$/PI${}^{-}$. Results are presented as the average percentage of cells from three independent experiments. The experimental groups were compared with the matching control groups (normoxic control or hypoxic control) or with other samples.

### 4.7. Analysis of Cell Cycle Distribution

To analyze the effect of SKR on the whole population of cells, the cell cycle distribution of cells was examined. Adherent cells were harvested by trypsinization and together with floating cells were washed with cold PBS, centrifuged, fixed in cold 70% ethanol and stored at −20 ${}^{\circ }C$ (at least overnight) for further analysis. Fixed cells were subsequently centrifuged, washed with PBS, and stained with staining solution (20 μg/mL PI, 137 μg/mL RNAse A and 0.1% Triton X-100 in PBS). After 30 min incubation in the dark, samples were measured with BD FACSCalibur flow cytometer (BD Biosciences, San Jose, CA, USA). To analyze the raw files, ModFit 3.0 (Verity Software House, Topsham, ME, USA) was used. Only debris-free single cells were analyzed. Examples of gating strategies are presented in the Supplementary Materials (Figures S30–S37). The results are presented as the average ratio of cells in the individual phase to all cells from three independent experiments. The experimental groups were compared with the matching control groups (normoxic control or hypoxic control).

### 4.8. Colony Forming Assay

For the analysis of the colony-forming ability, HCT 116 and HT-29 cells from hypoxia and normoxia were harvested by trypsinization, washed with PBS, centrifuged and resuspended in PBS. Then, cells were counted using Vi-CELL XR Cell Viability Analyzer (Beckman Coulter, Indianapolis, IN, USA) and seeded as 500 cells per well in fresh medium in a six-well plate (TPP Techno Plastic Products AG, Trasadingen, Switzerland). After 8 days of incubation, the medium was removed and formed colonies were washed with PBS and stained with methylene blue dye in methanol (0.8%). Afterwards, the dye was removed and the colonies were washed with PBS and subsequently scanned. All independent experiments were performed in technical duplicate (two wells from each sample) in three independent experiments. Colonies were counted using ImageJ (version 1.8.0; NIH, Bethesda, MA, USA) using the Colony Counter plugin (version 0.9), manually checked and corrected as needed. The results are presented as the number of colonies. Values are presented as technical duplicates from biological triplicates for each sample. To avoid the effect of the solvent, the control sample contained the same amount of DMSO as a sample with a higher concentration of SKR. The experimental groups were compared with the matching control groups (normoxic control or hypoxic control).

### 4.9. Mass Spectrometry

Mass spectrometry analysis (MS) was performed in the Proteomic Core Facility of Central European Institute of Technology (CEITEC), Brno, Czech Republic. Based on our previous results, HCT 116 cell line treated with SKR (10 μM) for 48 h in normoxia or hypoxia and a matching control were chosen for this analysis. Biological triplicates of all samples were prepared.

#### 4.9.1. Sample Preparation

Samples were washed three times with ice-cold PBS to prevent the contamination of samples with serum proteins in cultivation media. Then, cells were harvested by scraping in ice-cold PBS and subsequently centrifuged. Pellets were resuspended in 50 μL of SDT lysis buffer (4% sodium dodecyl sulfate (SDS), 0.1% dithiothreitol (DTT) in Tris/HCl pH 7.6) and homogenized for 120 min at 95 ${}^{\circ }C$ on aThermoMixer (Eppendorf, Hamburg, Germany). After perfect homogenization, samples were stored at −80 ${}^{\circ }C$. Samples for LC-MS analyses were prepared using filter-assisted sample preparation (FASP; Merck Millipore, Burlington, MA, USA) from 5 μL of the protein mixture. Protein reduction and alkylation with iodoacetamide and 30 kDa cut-off filters were used. Samples were incubated for 18 h with trypsin at 37 ${}^{\circ }C$, and resulting peptides were extracted into 15 μL of the final solution.

#### 4.9.2. MS Measurement and Analysis

Mass spectrometry analysis of the peptide mixture was done using the RSLCnano system, which was connected online to an Orbitrap Q-Exactive HF-X system (Thermo Fisher Scientific, Waltham, MA, USA). Prior to LC separation, tryptic digests were concentrated and desalted online using a cartridge trapping column (300 μm × 5 mm) filled with 5 μm particles C18 PepMap100 sorbent (Thermo Fisher Scientific, Waltham, MA, USA). The peptides were eluted from the trapping column onto an Acclaim Pepmap100 C18 analytical column (3-μm particles, 75 μm × 500 mm; Thermo Fisher Scientific, Waltham, MA, USA) and separated by the following gradient program: mobile phase A: 0.1% formic acid in 5% DMSO water; mobile phase B: 0.1% formic acid and 5% DMSO in 80% acetonitrile, flow rate 300 nL/min. The gradient elution started at 2% (0–5th min) of mobile phase B, increased from 2% to 35% (5th–105th min), then increased linearly to 90% (105th–110th min) of mobile phase B and remained at this state for the next 10 min. The equilibration of the trapping column and the column was done prior to sample injection to the sample loop. For quality control purposes, Biognosys iRT peptides (Biognosys AG, Schlieren, Switzerland) were added during each analysis. The samples were measured in a random order with a blank analysis for the samples. Approximately 2000 ng was subjected to LC-MS analysis per sample. The analytical column outlet was directly linked to the Digital PicoView 550 (New Objective, Woburn, MA, USA) ion source with sheath gas option and SilicaTip emitter (FS360-20-15-N-20-C12; New Objective, Woburn, MA, USA) utilization. An ABIRD (Active Background Ion Reduction Device; ESI Source Solutions, Woburn, MA, USA) was installed. MS data were acquired in a data-dependent strategy, selecting up to the top 20 precursors based on precursor abundance in the survey scan (350–2000 m/z). The resolution of the survey scan was 120,000 (at 200 m/z) with a target value of 3 × 10^6^ ions and a maximum injection time of 100 ms. HCD MS/MS spectra were acquired with a target value of 1 × 10^5^ and resolution of 15,000 (at 200 m/z). The maximum injection time for MS/MS was 50 ms. Dynamic exclusion was enabled for 40 s after one MS/MS spectrum acquisition. The isolation window for MS/MS fragmentation was set to 1.2 m/z.

LC-MS data processing was done using MaxQuant software (version 1.6.2.10; Max Planck Institute of Biochemistry, Martinsried, Germany) with Andromeda search engine [91,92]. UniProtKB database (taxonomy: human; taxon ID: 9606; version 180912) and MaxQuant contaminant database of 247 protein sequences were used. Matches between runs were used across biological replicates datasets to improve peptide matching. Results were set to follow a 1% false discovery rate of peptide-to-spectrum matches and protein levels. The minimal number of unique and razor peptides was set to 1. The mass spectrometry proteomics data have been deposited to the ProteomeXchange Consortium via the PRIDE partner repository with the dataset identifier PXD019995. Intensities of identified proteins were normalized across all samples prior to further data evaluation. Several normalization approaches were tested and the LoessF normalization was finally selected. Log2 intensities were calculated and used for further analysis. In addition, Pearson’s pairwise correlation coefficients were calculated, using data for all protein groups with a non-zero protein group area for the given pair of samples for sample cluster analysis. Comparative proteomics was performed by the LIMMA test and the Benjamini Hochberg method. Fold change (FC) and Log2FC values were also calculated and exported as CSV data. This analysis was performed in the KNIME analytics platform (KNIME AG, Zurich, Switzerland) using the KNIME proteomic workflow created by the CEITEC MU Proteomics laboratory (available under GPL-3.0 license at https://github.com/OmicsWorkflows, accessed on 27 August 2019). For the final report, the following parameters were set as follows: changed proteins needed to have a Log2FC higher than 1.0 or lower than −1.0 between treated samples to control samples and to be significantly changed simultaneously in both conditions (hypoxia and normoxia).

### 4.10. RT-qPCR Analysis

The normalized relative mRNA levels of all analyzed genes (Table 5) were quantified by real-time RT-qPCR with SYBR Green I detection of the amplicons. The levels of the studied genes were normalized with the reference gene *PMM1* (Gene ID: 5372). The cDNA template for RT-qPCR was prepared from total RNA by RevertAid-MuLV Reverse Transcriptase (RevertAid First Strand cDNA Synthesis Kit, Thermo Fisher Scientific Inc., Waltham, MA, USA) and a random primer mix (a mixture of anchored-dT and random hexamer primers; NEB, Ipswich, MA, USA). Total RNA was isolated by TRI Reagent (MRC Inc, Cincinnati, OH, USA) according to the manufacturer’s instructions. RNA integrity was verified by agarose gel electrophoresis and the amount of RNA/μL was determined using a BioSpec nano-spectrophotometer (Shimadzu, Kyoto, Japan). Only samples with distinct 28S/18S RNA bands were used for cDNA transcription. Human-specific primers for the respective genes were designed by PrimerBLAST [93] hosted at the NCBI web page. The primers either spanned two neighboring exons or were bound to different exons. Primer pairs with no secondary structures and the best delta G values were chosen by Unipro UGENE v1.20.0 software [94].

RT-qPCR experiments were performed using a CFX96 Touch Real-Time PCR Detection System (BioRad Laboratories, Hercules, CA, USA). For amplification reactions, Xceed qPCR SG Mix, Lo-Rox (IAB, Prague, Czech Republic), 0.5 μM For/Rev primers and 15 ng of RNA/cDNA were used. The conditions for RT-qPCR were according to Majernik et al. [49]. The amplification of single and specific gene fragments in the RT-qPCR was confirmed by melt curve analysis and by checking the size of the amplicon by agarose gel electrophoresis. The melt curves for all used primer pairs used are shown in the Supplementary Materials (Figures S38–S46). All samples were analyzed in biological triplicates from independently repeated experiments. The resulting values of target mRNA levels were normalized to the levels of *PMM1*, which was shown to be an accurate internal reference control in HCT 116, HT-29 and SW620 cell lines. The results are presented as the *PMM1*-normalized mRNA level. The experimental groups were compared with the matching control groups (normoxic control or hypoxic control).

### 4.11. Statistical Analysis

Data were analyzed and visualized using in the R environment [95] (version 4.0.3) using the standard library and libraries noted listed in Table S1 in the Supplementary Materials [95,96,97,98,99,100,101,102,103,104,105,106,107,108]. Significance between samples was assessed by a one-way ANOVA with Tukey’s post-test. Significance levels are presented as follows: * *p* < 0.05, ** *p* < 0.01, *** *p* < 0.001. For comparative proteomics, the LIMMA test and the Benjamini Hochberg method was performed. Significance levels are indicated in the capture for each figure.

## 5. Conclusions

In this article, we present a comprehensive analysis of the effect of the natural secondary metabolite SKR on cancer cells. We described the SKR-induced changes on many different levels, from the overall influence on the cellularity and metabolic activity of cells through the effects on whole cell populations to mass spectrometry analysis on the molecular level. Many biomolecules are currently used in cancer treatment, and we demonstrated that SKR possesses comparable features with other drugs. SKR has a significantly negative effect on cancer cells, induces apoptosis, upregulates DR5 in normoxic as well as hypoxic conditions without an effect on pathologically unchanged healthy cells and reverses TRAIL resistance in hypoxia as well as in TRAIL-resistant cell lines. Our results indicate the possible usage of SKR as an antitumor drug or as an adjuvant to other established treatments by increasing the accumulation of structurally similar molecules or by the sensitization of cells to TRAIL-targeted treatment.

## Figures and Tables

**Table 1:** SKR concentration resulting in a 25% decrease of metabolic activity based on MTT assay for cancer cell lines. Values are presented as concentrations in μM with standard deviation (SD) values for all used times and conditions.

	24 h Normoxia	24 h Hypoxia	48 h Normoxia	48 h Hypoxia
**HCT 116**	17.88 ± 1.56	21.96 ± 2.19	9.50 ± 0.80	6.70 ± 0.34
**HT-29**	25.67 ± 2.80	16.45 ± 1.17	15.83 ± 1.15	11.36 ± 0.65

**Table 2:** List of significantly changed protein groups after SKR treatment in hypoxia (H) and normoxia (N). Log2FC is calculated between SKR-treated samples and control. Upregulation and downregulation labels: ↑ Log2FC ≥ +1.5 (upregulated); ↓ Log2FC ≤−1.5 (downregulated).

Accession	Description of Protein Group (Gene Name)	Log2FC (H)	adj.p Value (H)	Log2FC (N)	adj.p Value (N)	Hypoxia	Normoxia
O14763	Tumor necrosis factor receptor superfamily member 10B (GN = TNFRSF10B)	3.66	0	2.48	0.01	↑	↑
Q6IQ22	Ras-related protein Rab-12 (GN = RAB12)	2.39	0.05	2.33	0.01	↑	↑
Q9Y2H2	Phosphatidylinositide phosphatase SAC2 (GN = INPP5F)	3.65	0.01	1.05	0.03	↑	
P15407	Fos-related antigen 1 (GN = FOSL1)	1.52	0	2.91	0	↑	↑
Q9GZQ8	Microtubule-associated proteins 1A/1B light chain 3B (GN = MAP1LC3B)	2.54	0	1.78	0	↑	↑
Q9HBI6	Phylloquinone omega-hydroxylase CYP4F11 (GN = CYP4F11)	1.36	0.04	2.89	0.01		↑
Q03405	Urokinase plasminogen activator surface receptor (GN = PLAUR)	1.95	0	2.22	0	↑	↑
Q7Z4Q2	HEAT repeat-containing protein 3 (GN = HEATR3)	2.09	0	1.82	0	↑	↑
Q53EU6	Glycerol-3-phosphate acyltransferase 3 (GN = GPAT3)	1.83	0.05	1.77	0	↑	↑
Q9BSH5	Haloacid dehalogenase-like hydrolase domain-containing protein 3 (GN = HDHD3)	3.01	0.01	0.44	0.02	↑	
P08243	Asparagine synthetase [glutamine-hydrolyzing] (GN = ASNS)	2.06	0	1.37	0	↑	
Q16850	Lanosterol 14-alpha demethylase (GN = CYP51A1)	1.84	0	1.33	0	↑	
Q9NUM4	Transmembrane protein 106B (GN = TMEM106B)	1.34	0.01	1.66	0.01		↑
H3BU86	STX16-NPEPL1 readthrough (NMD candidate) (GN = STX16-NPEPL1)	1.8	0.02	1.18	0.05	↑	
Q8ND82	Zinc finger protein 280C (GN = ZNF280C)	1.86	0	1.06	0.01	↑	
Q9H6H4	Receptor expression-enhancing protein 4 (GN = REEP4)	1.84	0.01	0.98	0.04	↑	
Q6WCQ1	Myosin phosphatase Rho-interacting protein (GN = MPRIP)	0.92	0.04	1.78	0		↑
O95352	Ubiquitin-like modifier-activating enzyme ATG7 (GN = ATG7)	1.56	0.01	1.11	0.02	↑	
Q8IVF2	Protein AHNAK2 (GN = AHNAK2)	1.93	0.02	0.7	0.05	↑	
O15231	Zinc finger protein 185 (GN = ZNF185)	1.51	0	1.09	0.05	↑	
Q9NQW6	Anillin (GN = ANLN)	0.52	0.04	1.84	0		↑
Q9Y617	Phosphoserine aminotransferase (GN = PSAT1)	1.5	0	0.83	0	↑	
Q9NR12	PDZ and LIM domain protein 7 (GN = PDLIM7)	1.51	0	0.72	0.01	↑	
Q9NP97	Dynein light chain roadblock-type 1 (GN = DYNLRB1)	−1.22	0.04	2.37	0		↑
Q8N5M9	Protein jagunal homolog 1 (GN = JAGN1)	−2.59	0.01	3.24	0	↓	↑
Q9NUQ3	Gamma-taxilin (GN = TXLNG)	−0.91	0.02	1.52	0.01		↑
P15153	Ras-related C3 botulinum toxin substrate 2 (GN = RAC2)	1.57	0.02	−1.14	0.01	↑	
P14635	G2/mitotic-specific cyclin-B1 (GN = CCNB1)	1.86	0	−1.45	0.04	↑	
Q9H2C0	Gigaxonin (GN = GAN)	−2.48	0.02	2.43	0.04	↓	↑
Q9UL03	Integrator complex subunit 6 (GN = INTS6)	−1.77	0	1.5	0	↓	
Q14BN4	Sarcolemmal membrane-associated protein (GN = SLMAP)	4.26	0.04	−4.77	0	↑	↓
Q9HCS7	Pre-mRNA-splicing factor SYF1 (GN = XAB2)	−1.79	0.02	0.65	0.04	↓	
P31483	Nucleolysin TIA-1 isoform p40 (GN = TIA1)	0.65	0.03	−1.83	0.02		↓
Q16555	Dihydropyrimidinase-related protein 2 (GN = DPYSL2)	−0.46	0.03	−1.53	0		↓
P82664	28S ribosomal protein S10, mitochondrial (GN = MRPS10)	−1.65	0	−0.45	0.03	↓	
P55011	Solute carrier family 12 member 2 (GN = SLC12A2)	−1.72	0.01	−0.45	0.04	↓	
Q9H8V3	Protein ECT2 (GN = ECT2)	−1.83	0.01	−0.42	0.04	↓	
Q9P2R3	Rabankyrin-5 (GN = ANKFY1)	−1.67	0.01	−0.88	0.01	↓	
P00395	Cytochrome c oxidase subunit 1 (GN = MT-CO1)	−0.91	0.03	−1.66	0		↓
Q92466	DNA damage-binding protein 2 (GN = DDB2)	−1.64	0.04	−1.15	0.03	↓	
Q9UHL4	Dipeptidyl peptidase 2 (GN = DPP7)	−1.68	0.03	−1.12	0.03	↓	
Q9BRK5	45 kDa calcium-binding protein (GN = SDF4)	−2.02	0.01	−0.81	0	↓	
Q6SPF0	Atherin (GN = SAMD1)	−1.49	0.03	−1.59	0.01		↓
Q9NRX4	14 kDa phosphohistidine phosphatase (GN = PHPT1)	−1.76	0	−1.32	0.01	↓	
P14406	Cytochrome c oxidase subunit 7A2, mitochondrial (GN = COX7A2)	−2.14	0	−1.01	0	↓	
P35611	Alpha-adducin (GN=ADD1)	−1.61	0.01	−1.56	0	↓	↓
Q52LW3	Rho GTPase-activating protein 29 (GN = ARHGAP29)	−2.1	0	−1.09	0	↓	
Q6UX53	Methyltransferase-like protein 7B (GN = METTL7B)	−1.73	0	−1.69	0	↓	↓
P22830	Ferrochelatase, mitochondrial (GN = FECH)	−1.81	0.01	−1.91	0.01	↓	↓
O94832	Unconventional myosin-Id (GN = MYO1D)	−2.52	0.01	−1.23	0.01	↓	
P33552	Cyclin-dependent kinases regulatory subunit 2 (GN = CKS2)	−1.77	0.01	−2.01	0	↓	↓
Q92817	Envoplakin (GN = EVPL)	−2.76	0.01	−1.54	0.01	↓	↓
A6NJ78	Probable methyltransferase-like protein 15 (GN = METTL15)	−2.16	0.02	−3.27	0	↓	↓
P50748	Kinetochore-associated protein 1 (GN = KNTC1)	−3.94	0	−2.16	0.02	↓	↓
Q14517	Protocadherin Fat 1 (GN = FAT1)	−5.73	0	−0.49	0.05	↓	
P46934	E3 ubiquitin-protein ligase NEDD4 (GN = NEDD4)	−3.7	0.01	−3.94	0	↓	↓

**Table 3:** List of pathways significantly connected to upregulated proteins. As the input for Reactome analysis, we used a list of all protein groups that were significantly changed (adj. *p* value < 0.05) between control and sample in both conditions and upregulated (Log2FC > 1). Listed are 20 pathways with a higher *p*-value; each entity’s *p*-value and FDR was calculated by the Reactome engine.

Pathway Name	Entities Found	Entities Total	Entities’ *p*-Value	Entities’ FDR
Rab regulation of trafficking	2	123	0.00236	0.0398
Attachment of GPI anchor to uPAR	1	7	0.00429	0.0398
TRAIL signaling	1	8	0.0049	0.0398
Regulation by c-FLIP	1	11	0.00674	0.0398
Dimerization of procaspase-8	1	11	0.00674	0.0398
Receptor-mediated mitophagy	1	11	0.00674	0.0398
Pexophagy	1	11	0.00674	0.0398
TP53 regulates transcription of death receptors and ligands	1	12	0.00735	0.0398
Dissolution of Fibrin Clot	1	13	0.00796	0.0398
CASP8 activity is inhibited	1	14	0.00857	0.0421
Caspase activation via death receptors in the presence of ligand	1	19	0.0116	0.0421
Regulation of necroptotic cell death	1	21	0.0128	0.0421
Pink/Parkin mediated mitophagy	1	22	0.0134	0.0421
RIPK1-mediated regulated necrosis	1	23	0.014	0.0421
Regulated necrosis	1	23	0.014	0.0421
Mitophagy	1	29	0.0177	0.0474
Caspase activation via extrinsic apoptotic signaling pathway	1	29	0.0177	0.0474
Synthesis of PA	1	39	0.0237	0.0474
NGF-stimulated transcription	1	39	0.0237	0.0474

**Table 4:** List of pathways significantly connected to downregulated proteins. As an input for Reactome analysis, we used a list of all protein groups that were significantly changed (adj. *p* value < 0.05) between the control and sample in both conditions and downregulated (Log2FC < −1). All pathways are listed; each entity’s *p*-value and FDR was calculated by the Reactome engine.

Pathway Name	Entities Found	Entities Total	Entities’ *p*-Value	Entities’ FDR
Regulation of PTEN localization	1	9	0.0063	0.126
Downregulation of ERBB4 signaling	1	11	0.0077	0.126
Caspase-mediated cleavage of cytoskeletal proteins	1	12	0.00839	0.126
Heme biosynthesis	1	15	0.0105	0.126
Miscellaneous transport and binding events	1	26	0.0181	0.126
Metabolism of porphyrins	1	28	0.0195	0.126
Apoptotic cleavage of cellular proteins	1	38	0.0264	0.126
XBP1(S) activates chaperone genes	1	48	0.0332	0.126
IRE1alpha activates chaperones	1	50	0.0346	0.126
Apoptotic execution phase	1	52	0.0359	0.126
Signaling by ERBB4	1	62	0.0427	0.126
Regulation of PTEN stability and activity	1	69	0.0474	0.126

**Table 5:** Sequences of used RT-qPCR primers. Annealing Temperatures (Ta) and Product Lenghts are in table.

Gene (NCBI Gene ID)	GenBank Ref.seq	Primer Name	Sequence	Ta and Product Length
*TNFRSF10B*	NM_003842	DR5_for	GTGATTCAGGTGAAGTGGAGC	60 ${}^{\circ }C$
(8795)	NM_147187	DR5_rev	CGACCTTGACCATCCCTCTG	147 bp
*TNFRSF10B*	NM_003842	DR5.v1_for	ACTCCTGCCTCTCCCTGTTC	60 ${}^{\circ }C$
(8795)		DR5.v1_rev	AGGTCGTTGTGAGCTTCTGTC	186 bp
*TNFRSF10B*	NM_147187	DR5.v2_for	CTAAGTCCCTGCACCACGAC	60 ${}^{\circ }C$
(8795)		DR5.v2_rev	TGACTCCTATGATGATGCCTGATT	193 bp
*TNFRSF10A*	NM_003844	DR4_for	GTTGGTGGCTGTGCTGATTG	60 ${}^{\circ }C$
(8797)		DR4_rev	TGCGTTGCTCAGAATCTCGT	151 bp
*TNF* (TNF alpha)	NM_000594	TNFa_for	TGTCCACACGATCCCAACAC	60 ${}^{\circ }C$
(7124)		TNFa_rev	GGCTGTCACACCCACAATCA	152 bp
*TNFRSF1A*	NM_001065	TNFRSF1A_for	CGTGATCTCTATGCCCGAGT	60 ${}^{\circ }C$
(7132)		TNFRSF1A_rev	GACCAGTCCAATAACCCCTGA	244 bp
*TNFRSF1B*	NM_001066	TNFRSF1B_for	TGTCCACACGATCCCAACAC	60 ${}^{\circ }C$
(7133)		TNFRSF1B_rev	GGCTGTCACACCCACAATCA	152 bp
*TNFRSF6B*	NM_003823	DcR3_for	CTCTTCCTCCCATGACACCCT	60 ${}^{\circ }C$
(8771)		DcR3_rev	ATGGAGATGTCCTGGAAAGCC	123 bp
*PMM1*	NM_002676	PMM1_for	GCTCGCCAGAAAATTGACCCT	61 ${}^{\circ }C$
(5372)		PMM1_rev	ATACTGCACCGTCCCGTTCT	177 bp

## Data Availability

Mass spectrometry data are available via ProteomeXchange with identifier PXD019995.

## Supplementary Materials

The supplementary materials are available online https://www.mdpi.com/article/10.3390/cancers13071646/s1, Data S1: List of all identified protein groups, Table S1: Used R libraries, Figure S1: The effect of SKR on average diameter of cells, Figure S2: The effect of SKR on cellularity, Figure S3: The effect of SKR on proportions of cells in apoptosis, secondary necrosis, necrosis and live cells, Figure S4: The effect of SKR on cell cycle distribution, Figure S5: Normalized mRNA levels of TNFRSF10B in different cell lines in different times, Figure S6: Phosphatidylserine externalization analysis gating strategy example (HCT 116, hypoxia, Control, 48 h), Figure S7: Phosphatidylserine externalization analysis gating strategy example (HCT 116, hypoxia, 10 ng/mL TRAIL, 48 h), Figure S8: Phosphatidylserine externalization analysis gating strategy example (HCT 116, hypoxia, 10 µM SKR, 48 h), Figure S9: Phosphatidylserine externalization analysis gating strategy example (HCT 116, hypoxia, 10 µM and 10 ng/mL TRAIL, 48 h), Figure S10: Phosphatidylserine externalization analysis gating strategy example (HCT 116, normoxia, Control, 48 h), Figure S11: Phosphatidylserine externalization analysis gating strategy example (HCT 116, normoxia, 10 ng/mL TRAIL, 48 h), Figure S12: Phosphatidylserine externalization analysis gating strategy example (HCT 116, normoxia, 10 µM SKR, 48 h), Figure S13: Phosphatidylserine externalization analysis gating strategy example (HCT 116, normoxia, 10 µM and 10 ng/mL TRAIL, 48 h), Figure S14: Phosphatidylserine externalization analysis gating strategy example (HT-29, hypoxia, Control, 48 h), Figure S15: Phosphatidylserine externalization analysis gating strategy example (HT-29, hypoxia, 50 ng/mL TRAIL, 48 h), Figure S16: Phosphatidylserine externalization analysis gating strategy example (HT-29, hypoxia, 10 µM SKR, 48 h), Figure S17: Phosphatidylserine externalization analysis gating strategy example (HT-29, hypoxia, 10 µM and 50 ng/mL TRAIL, 48 h), Figure S18: Phosphatidylserine externalization analysis gating strategy example (HT-29, normoxia, Control, 48 h), Figure S19: Phosphatidylserine externalization analysis gating strategy example (HT-29, normoxia, 50 ng/mL TRAIL, 48 h), Figure S20: Phosphatidylserine externalization analysis gating strategy example (HT-29, normoxia, 10 µM SKR, 48 h), Figure S21: Phosphatidylserine externalization analysis gating strategy example (HT-29, normoxia, 10 µM and 50 ng/mL TRAIL, 48 h), Figure S22: Phosphatidylserine externalization analysis gating strategy example (SW620, hypoxia, Control, 48 h), Figure S23: Phosphatidylserine externalization analysis gating strategy example (SW620, hypoxia, 50 ng/mL TRAIL, 48 h), Figure S24: Phosphatidylserine externalization analysis gating strategy example (SW620, hypoxia, 10 µM SKR, 48 h), Figure S25: Phosphatidylserine externalization analysis gating strategy example (SW620, hypoxia, 10 µM and 50 ng/mL TRAIL, 48 h), Figure S26: Phosphatidylserine externalization analysis gating strategy example (SW620, normoxia, Control, 48 h), Figure S27: Phosphatidylserine externalization analysis gating strategy example (SW620, normoxia, 50 ng/mL TRAIL, 48 h), Figure S28: Phosphatidylserine externalization analysis gating strategy example (SW620, normoxia, 10 µM SKR, 48 h), Figure S29: Phosphatidylserine externalization analysis gating strategy example (SW620, normoxia, 10 µM and 50 ng/mL TRAIL, 48 h), Figure S30: Cell cycle analysis gating strategy example (HCT 116, normoxia, Control, 48 h), Figure S31: Cell cycle analysis gating strategy example HCT 116, normoxia, 10 µM SKR, 48 h, Figure S32: Cell cycle analysis gating strategy example HCT 116, hypoxia, Control, 48 h, Figure S33: Cell cycle analysis gating strategy example HCT 116, hypoxia, 10 µM SKR, 48 h, Figure S34: Cell cycle analysis gating strategy example HT-29, normoxia, Control, 48 h, Figure S35: Cell cycle analysis gating strategy example HT-29, normoxia, 10 µM SKR, 48 h, Figure S36: Cell cycle analysis gating strategy example HT-29, hypoxia, Control, 48 h, Figure S37: Cell cycle analysis gating strategy example HT-29, hypoxia, 10 µM SKR, 48 h, Figure S38: Melt curve after of the TNFRSF10B (DR5—both isoforms) gene fragment amplified in RT-qPCR by oligonucleotide primers DR5_for and DR5_rev, Figure S39: Melt curve after of the TNFRSF10B (DR5—isoform 1) gene fragment amplified in RT-qPCR by oligonucleotide primers DR5.v1_for and DR5.v1_rev, Figure S40: Melt curve after of the TNFRSF10B (DR5—isoform 2) gene fragment amplified in RT-qPCR by oligonucleotide primers DR5.v2_for and DR5.v2_rev, Figure S41: Melt curve after of the TNFRSF10A (DR4) gene fragment amplified in RT-qPCR by oligonucleotide primers DR4_for and DR4_rev, Figure S42: Melt curve after of the TNF (TNF alpha) gene fragment amplified in RT-qPCR by oligonucleotide primers TNF_for and TNF_rev, Figure S43: Melt curve after of the TNFRSF1A (TNFR1) gene fragment amplified in RT-qPCR by oligonucleotide primers TNFRSF1A_for and TNFRSF1A _rev, Figure S44: Melt curve after of the TNFRSF1B (TNFR2) gene fragment amplified in RT-qPCR by oligonucleotide primers TNFRSF1B_for and TNFRSF1B_rev, Figure S45: Melt curve after of the TNFRSF6B (DcR3) gene fragment amplified in RT-qPCR by oligonucleotide primers DcR3_for and DcR3_rev, Figure S46: Melt curve after of the PMM1 gene fragment, reference gene, amplified in RT-qPCR by oligonucleotide primers PMM1_for and PMM1_rev.

## Abbreviations

The following abbreviations are used in this manuscript:

ABCG2	ATP-binding cassette super-family G member 2
DMSO	Dimethylsulfoxid
DR5	Death receptor 5; known as TNFRSF10B (Tumor necrosis factor receptor superfamily 10B)
DTT	Dithiothreitol
FACS	Fluorescence-activated cell sorting
FDR	False discovery rate
GN	gene name
IC50	Half maximal inhibitory concentration
LC-MS	Liquid chromatography–mass spectrometry
MTT	3-(4,5-dimethylthiazol-2-yl)-2,5-diphenyltetrazolium bromide
PBS	Phosphate-buffered saline
PTEN	Phosphatase and tensin homolog
RT-qPCR	Reverse transcription quantitative polymerase chain reaction
SDS	Sodium dodecyl sulfate
SKR	Skyrin
TNF	Tumor necrosis factor
TRAIL	TNF-related apoptosis-inducing ligand
