# Supplementary material for: Death Receptor 5 (TNFRSF10B) Is Upregulated and TRAIL Resistance Is Reversed in Hypoxia and Normoxia in Colorectal Cancer Cell Lines after Treatment with Skyrin, the Active Metabolite of Hypericum spp
Source: Cancers (Basel). 2021 Apr 1;13(7):1646. doi: 10.3390/cancers13071646 (PMC8036732; doi:10.3390/cancers13071646)
Supplement: Supplementary file 1 [file cancers-13-01646-s001.zip › cancers-1060945-supplementary.pdf]

cancers

MDPI

Article

Supplementary Materials: Death Receptor 5 (TNFRSF10B) is Upregulated and TRAIL Resistance is Reversed in Hypoxia and Normoxia in Colorectal Cancer Cell Lines after Treatment with Skyrin, the Active Metabolite of *Hypericum* spp.

Marián Babinčák, Rastislav Jendželovský, Ján Košuth, Martin Majerník, Jana Vargová, Kamil Mikulášek, Zbyněk Zdráhal and Peter Fedoročko

Table S1. Used R libraries.

Name of Library	Version
dplyr	1.0.2
EnvStats	2.3.0
MASS	7.3-53
tidyr	1.1.2
lattice	0.20-41
ggplot2	3.3.2
ggsci	2.9
ggExtra	0.9
ggpubr	0.4.0
reshape2	1.4.4
tidyverse	1.3.0
gridExtra	2.3
GGally	2.0.0
forcats	0.5.0
ggimage	0.2.8
magick	2.6.0
kittyR	1.0.0

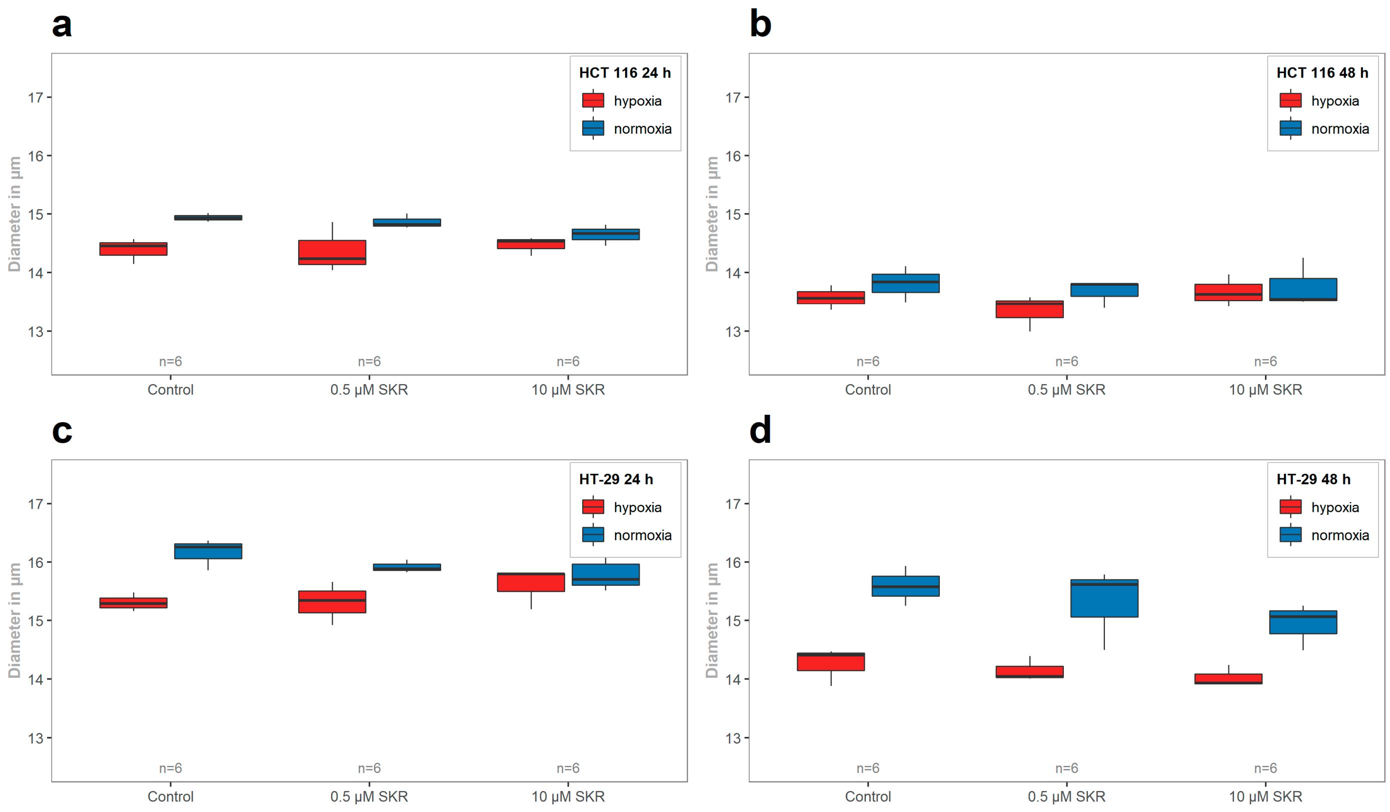

Figure S1. The effect of SKR on average diameter of cells. (**a**) HCT 116 after 24 h, (**b**) HCT 116 after 48 h, (**c**) HT-29 after 24 h, (**d**) HT-29 after 48 h. Number of samples (n) is noted in plots.

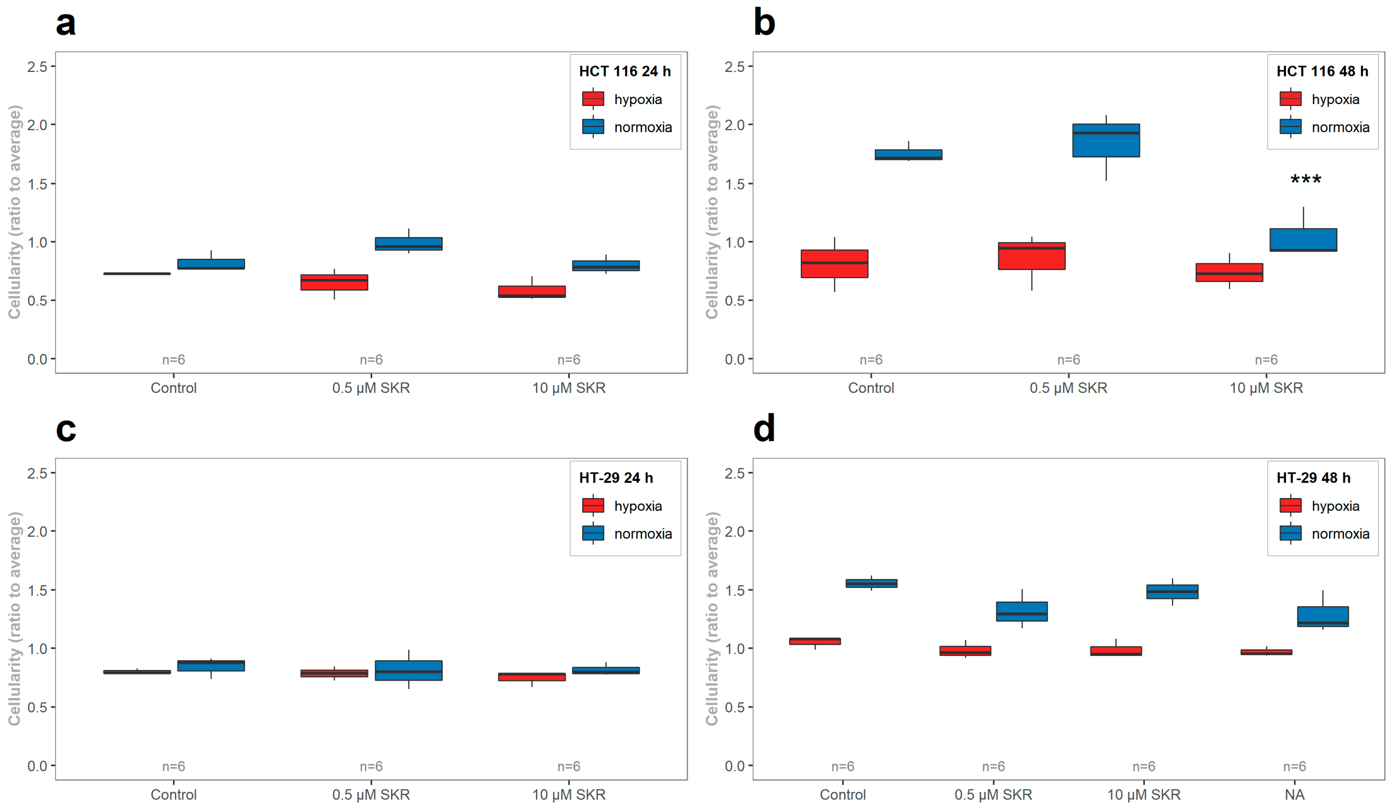

Figure S2. The effect of SKR on cellularity. (**a**) HCT 116 after 24 h, (**b**) HCT 116after 48 h, (**c**) HT-29 after 24 h, (**d**) HT-29 after 48 h. The experimental groups were compared with the control group. (*** $p < 0.001$). Number of samples (n) is noted in plots.

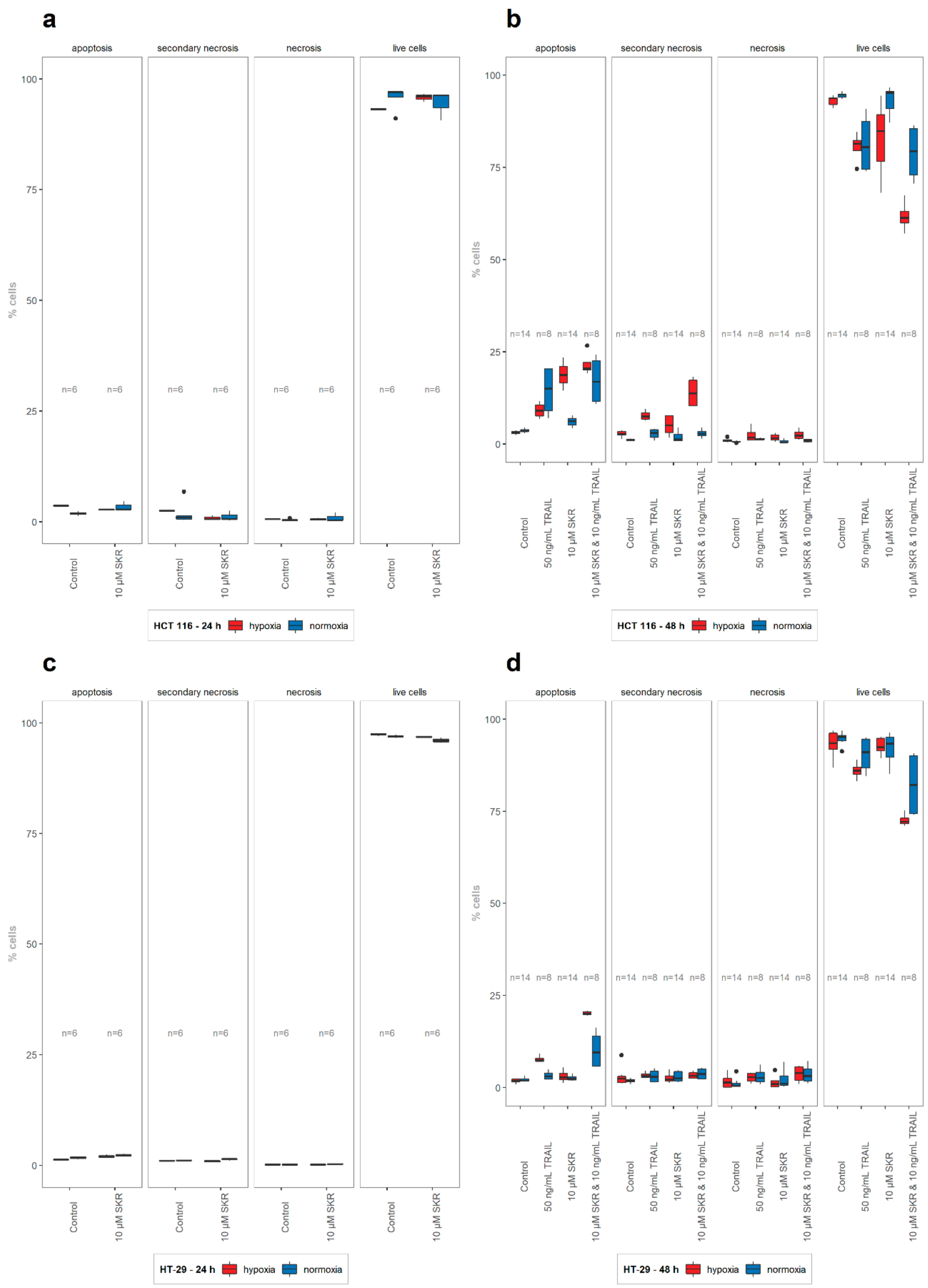

Figure S3. The effect of SKR on proportions of cells in apoptosis, secondary necrosis, necrosis, and live cells. (**a**) HCT 116 after 24 h, (**b**) HCT 116 after 48 h, (**c**) HT-29 after 24 h, (**d**) HT-29 after 48 h. Number of samples (n) is noted in plots.

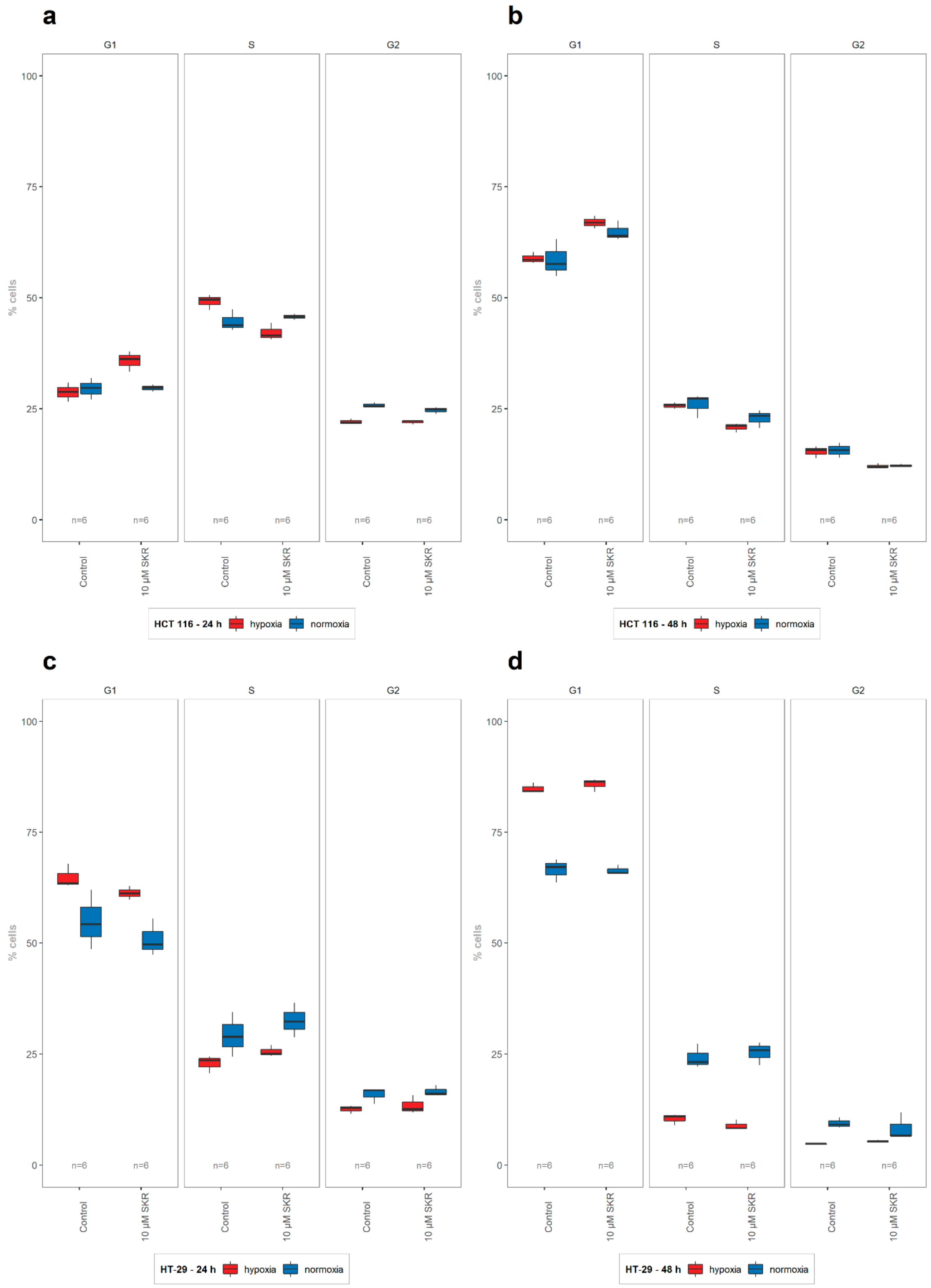

Figure S4. The effect of SKR on cell cycle distribution. (**a**) HCT 116 after 24 h, (**b**) HCT 116 after 48 h, (**c**) HT-29 after 24 h, (**d**) HT-29 after 48 h. Number of samples (n) is noted in plots.

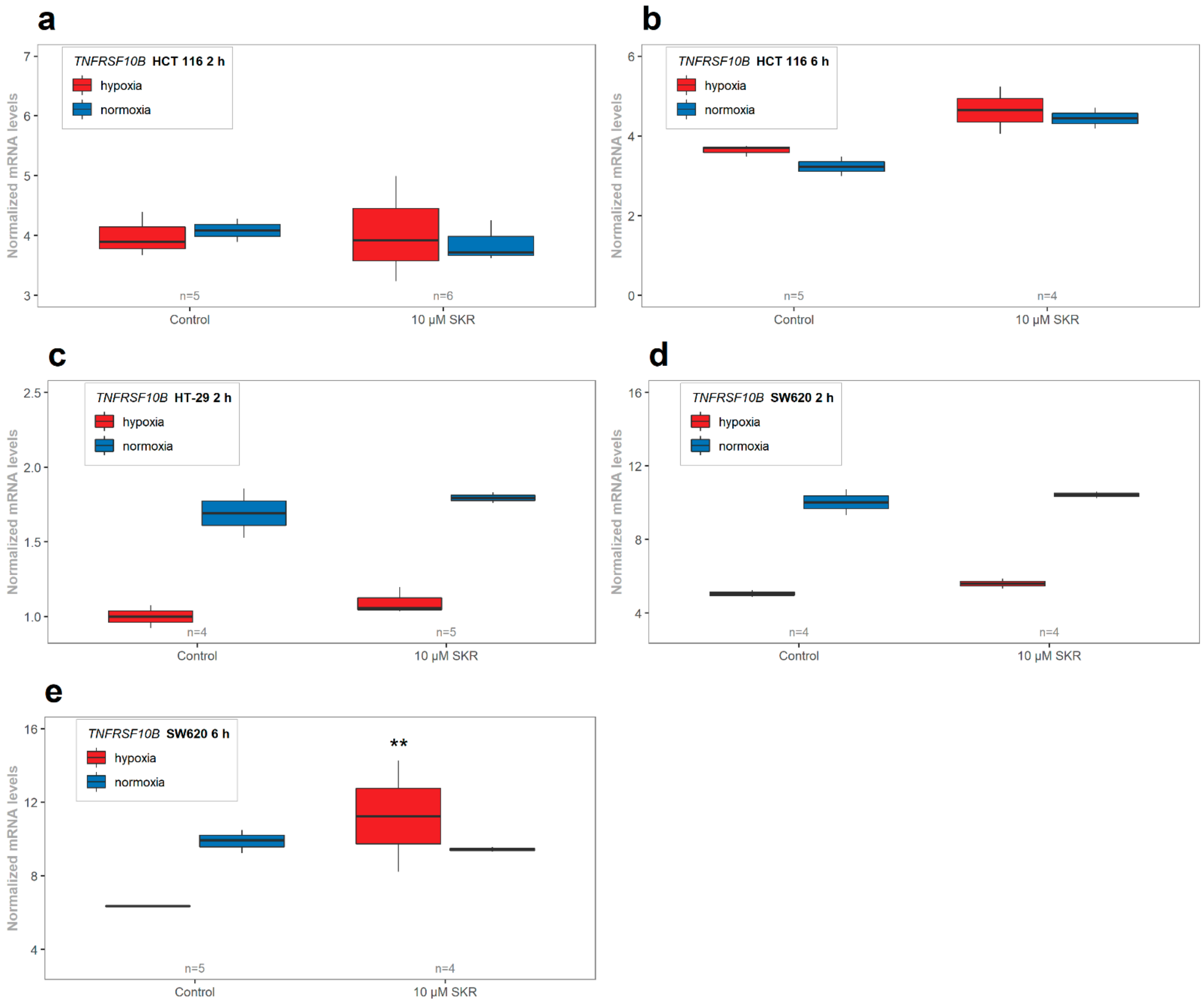

Figure S5. Normalized mRNA levels of *TNFRSF10B* in different cell lines in different times. (**a**) HCT 116 after 2 h, (**b**) HCT 116 after 6 h, (**c**) HT-29 after 2 h, (**d**) SW620 after 2 h, (**e**) SW620 after 6 h. The relative levels of levels of *TNFRSF10B* mRNA were evaluated by RT-qPCR and normalized to the level of reference gene *PMM1*. The experimental groups were compared with the matching control group. Number of samples (n) is noted in plots. (** $p < 0.01$).

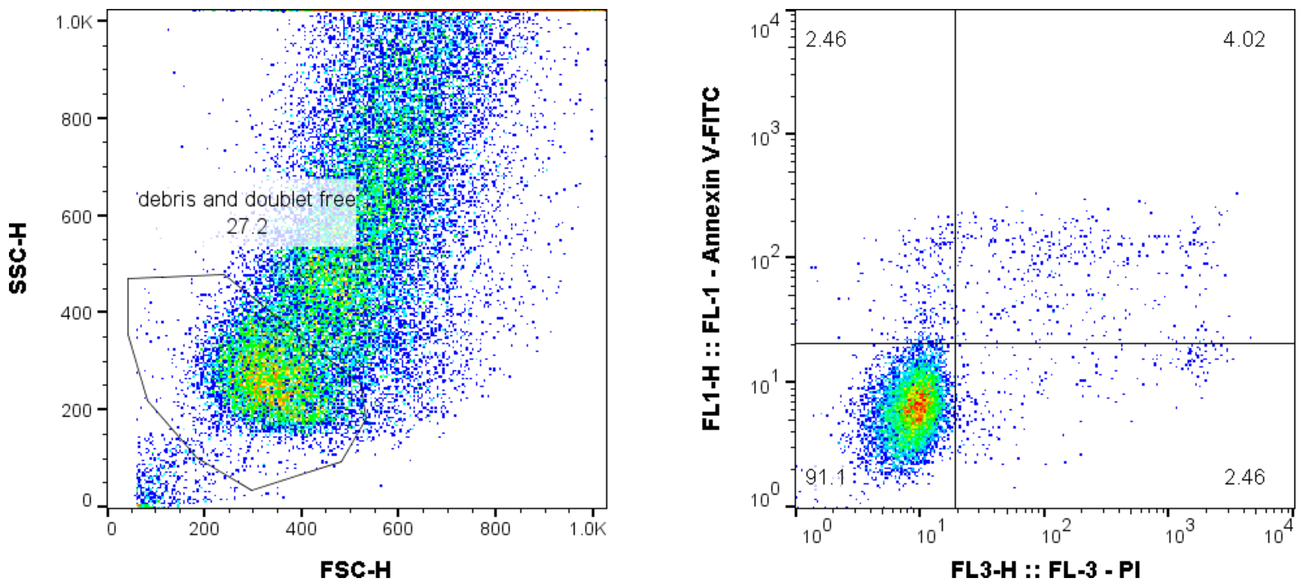

Figure S6. Phosphatidylserine externalization analysis gating strategy example (HCT 116, hypoxia, Control, 48 h).

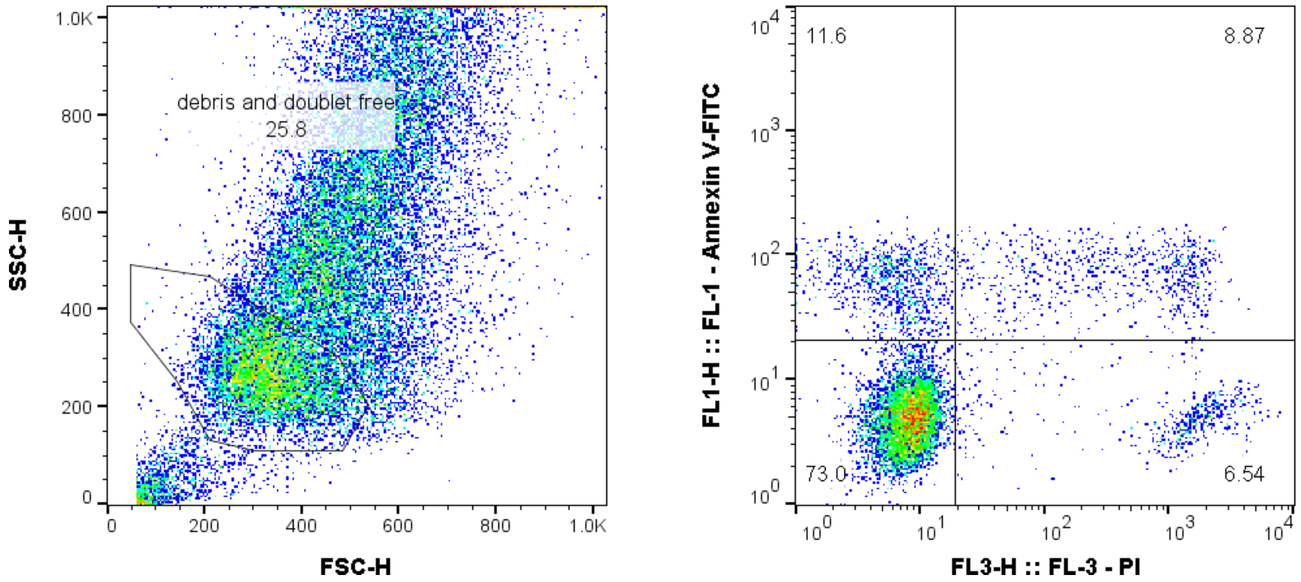

Figure S7. Phosphatidylserine externalization analysis gating strategy example (HCT 116, hypoxia, 10 ng/mL TRAIL, 48 h).

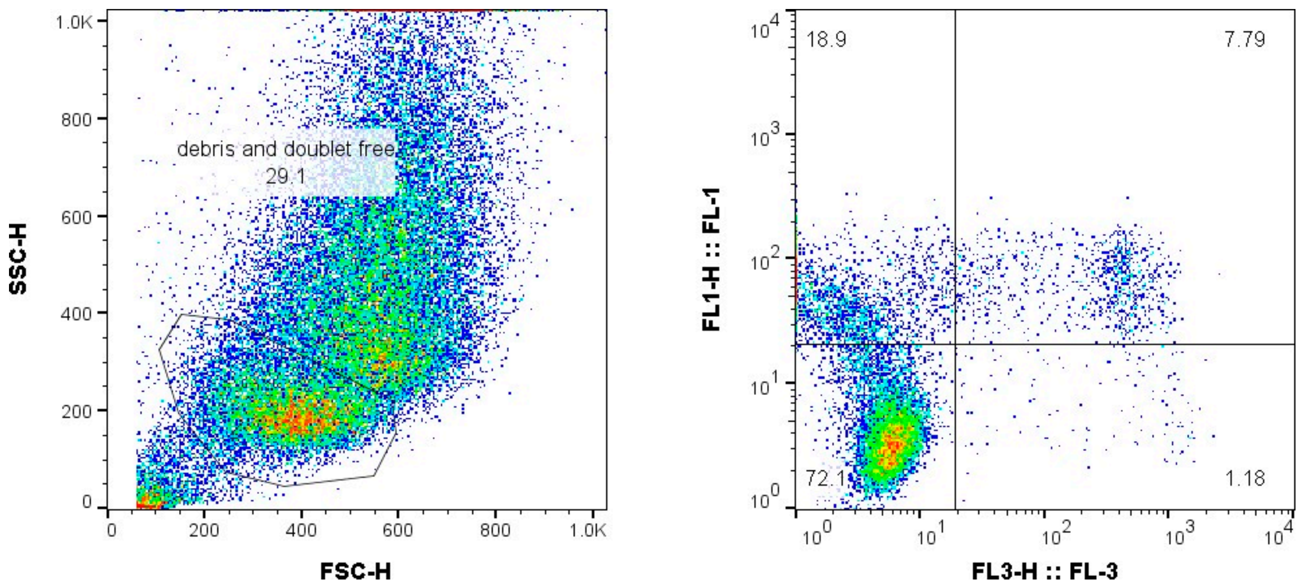

Figure S8. Phosphatidylserine externalization analysis gating strategy example (HCT 116, hypoxia, 10 µM SKR, 48 h).

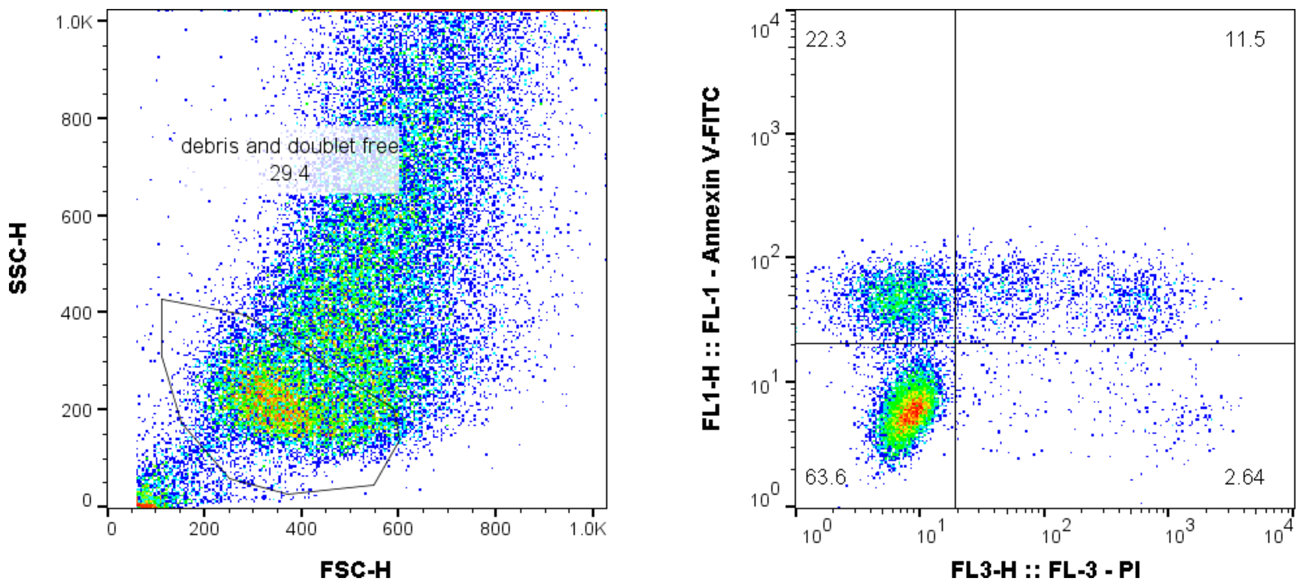

Figure S9. Phosphatidylserine externalization analysis gating strategy example (HCT 116, hypoxia, 10 µM & 10 ng/mL TRAIL, 48 h).

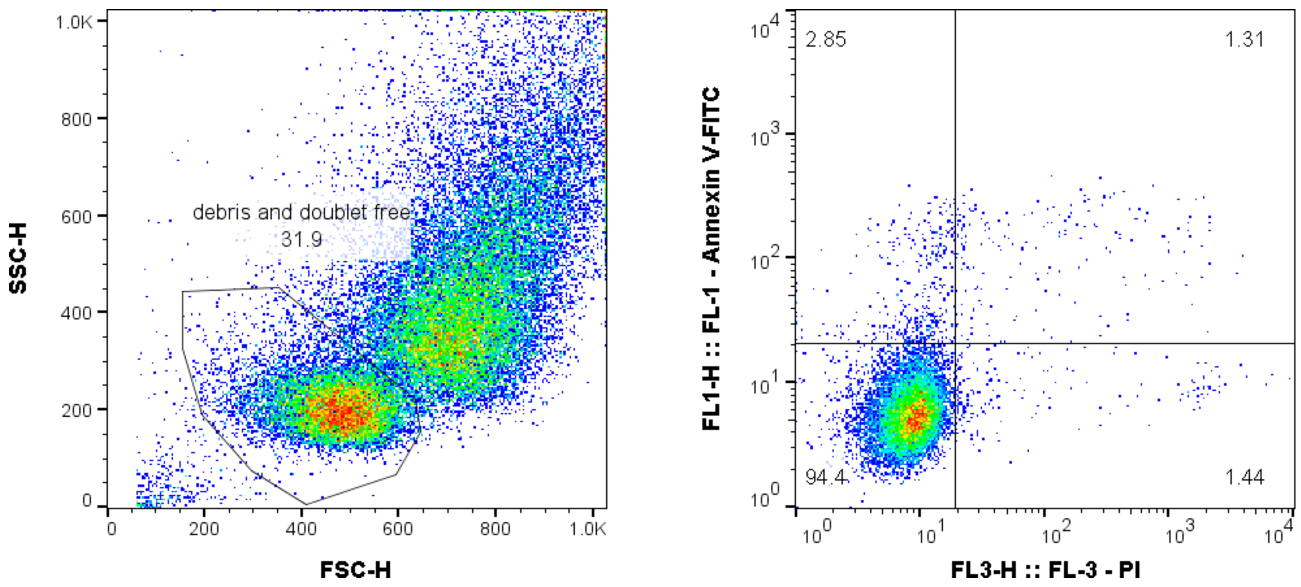

Figure S10. Phosphatidylserine externalization analysis gating strategy example (HCT 116, normoxia, Control, 48 h).

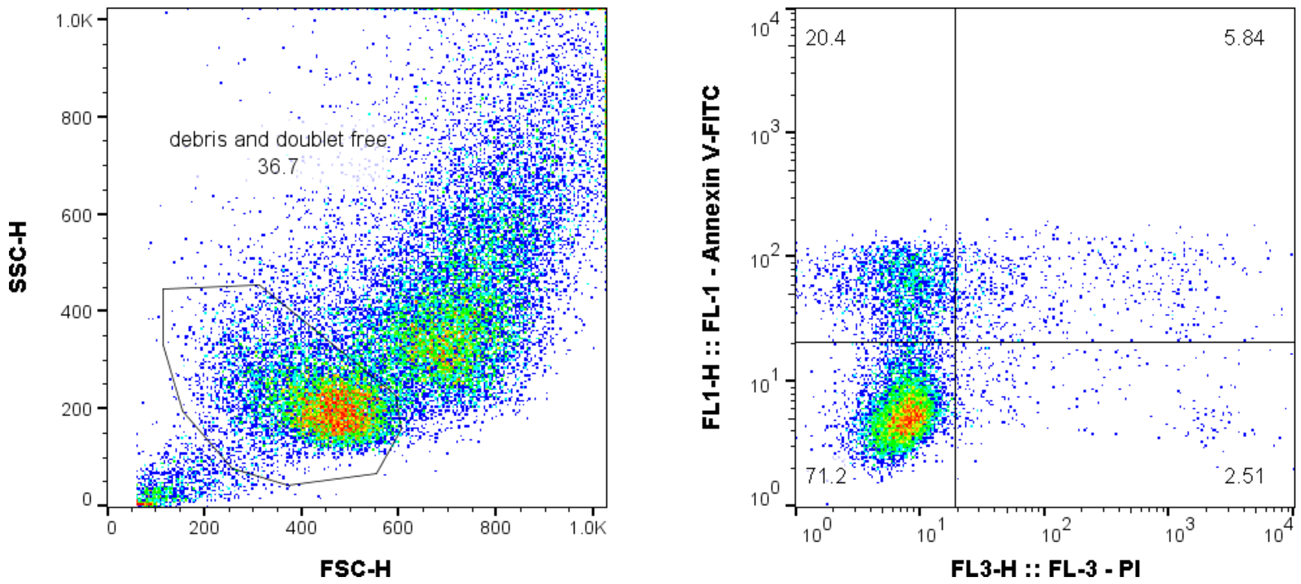

Figure S11. Phosphatidylserine externalization analysis gating strategy example (HCT 116, normoxia, 10 ng/mL TRAIL, 48 h).

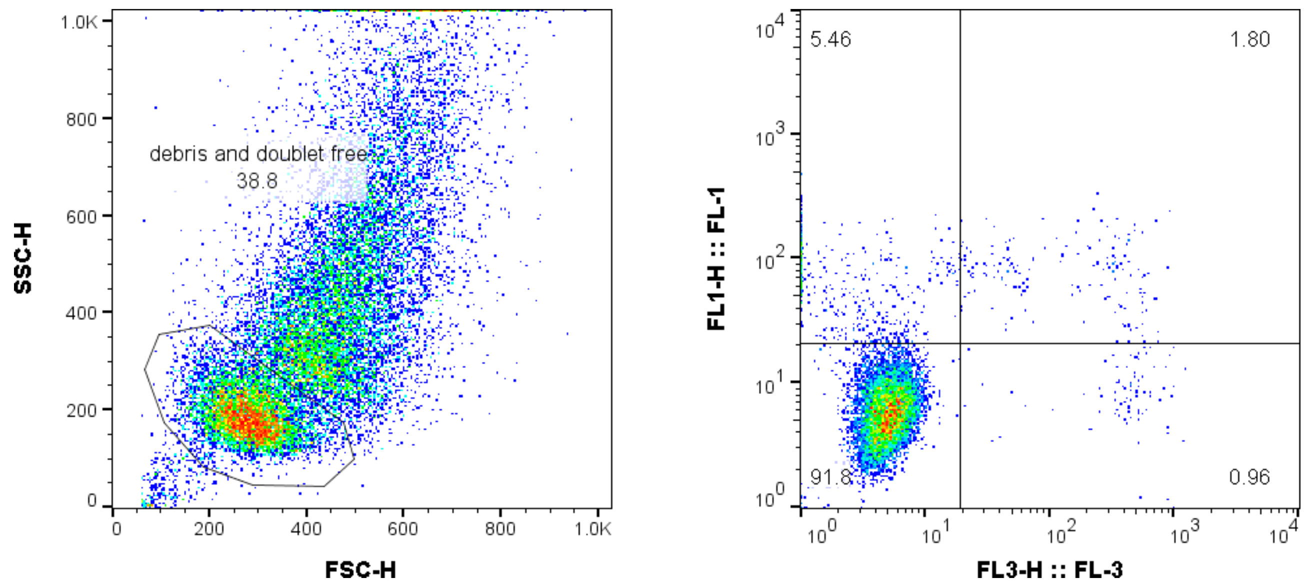

Figure S12. Phosphatidylserine externalization analysis gating strategy example (HCT 116, normoxia, 10 µM SKR, 48 h).

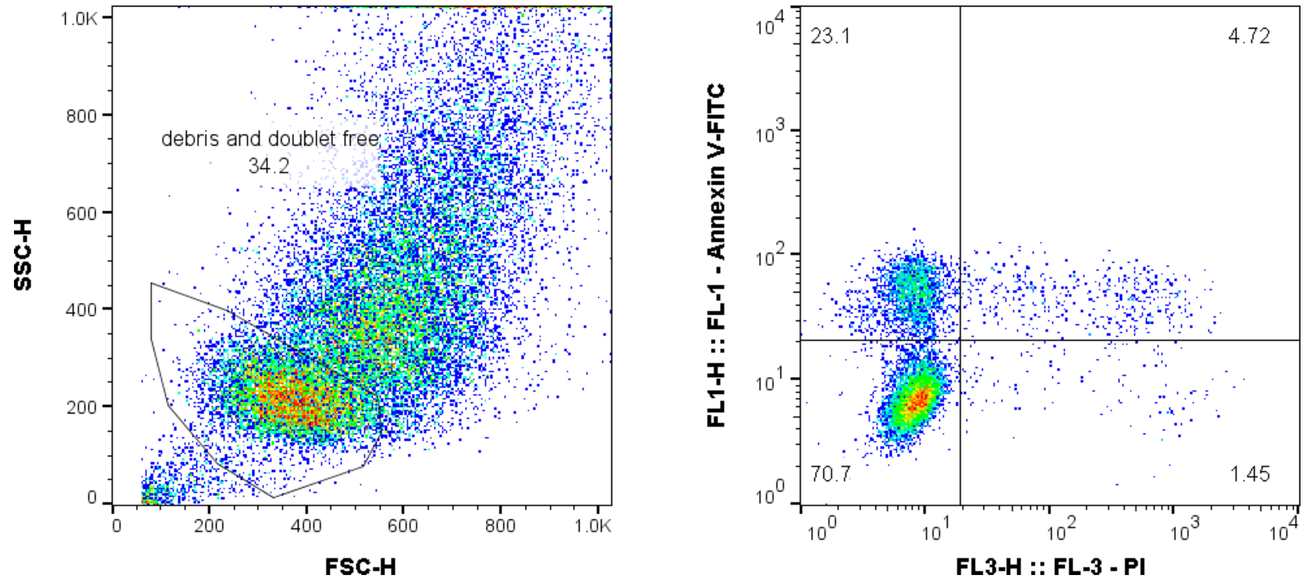

Figure S13. Phosphatidylserine externalization analysis gating strategy example (HCT 116, normoxia, 10 µM & 10 ng/mL TRAIL, 48 h).

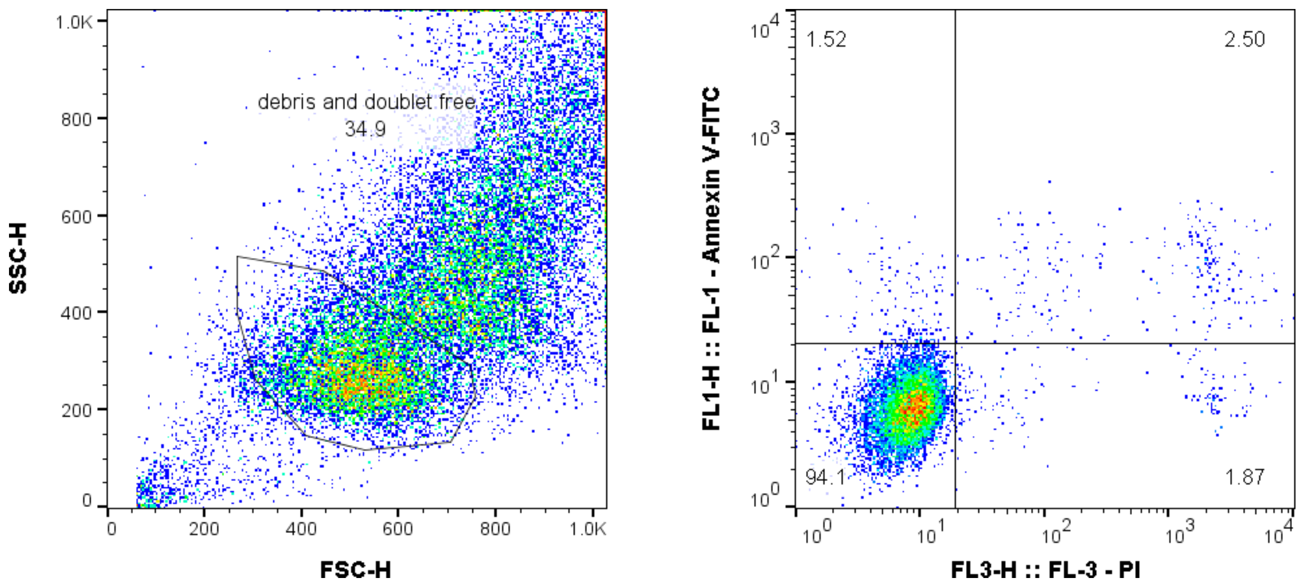

Figure S14. Phosphatidylserine externalization analysis gating strategy example (HT-29, hypoxia, Control, 48 h).

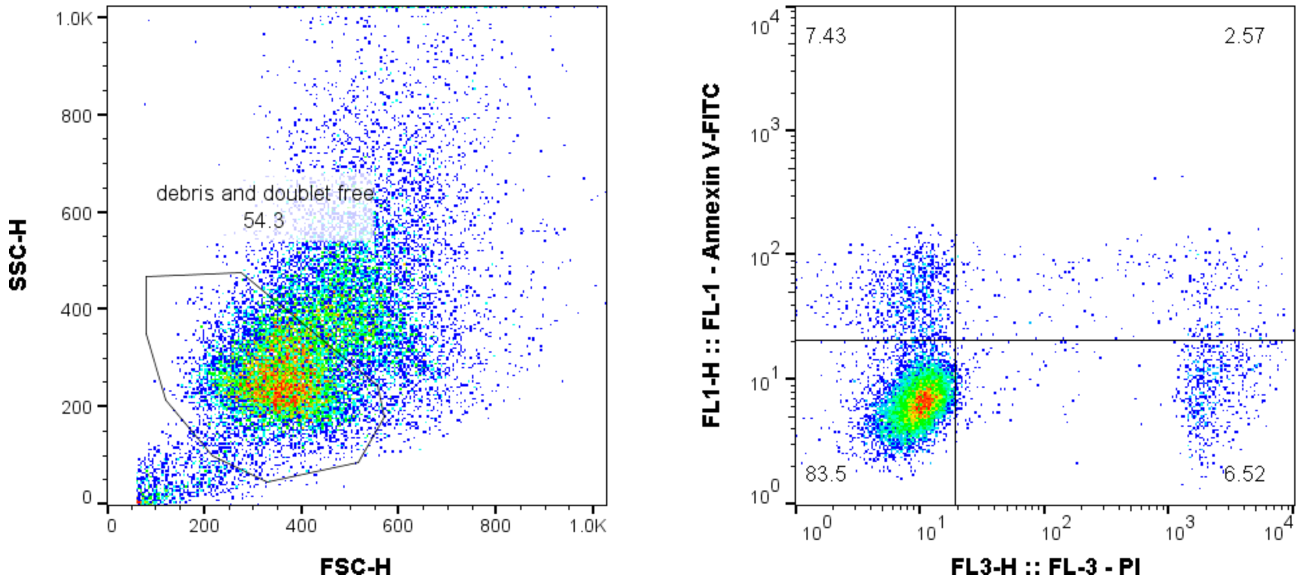

Figure S15. Phosphatidylserine externalization analysis gating strategy example (HT-29, hypoxia, 50 ng/mL TRAIL, 48 h).

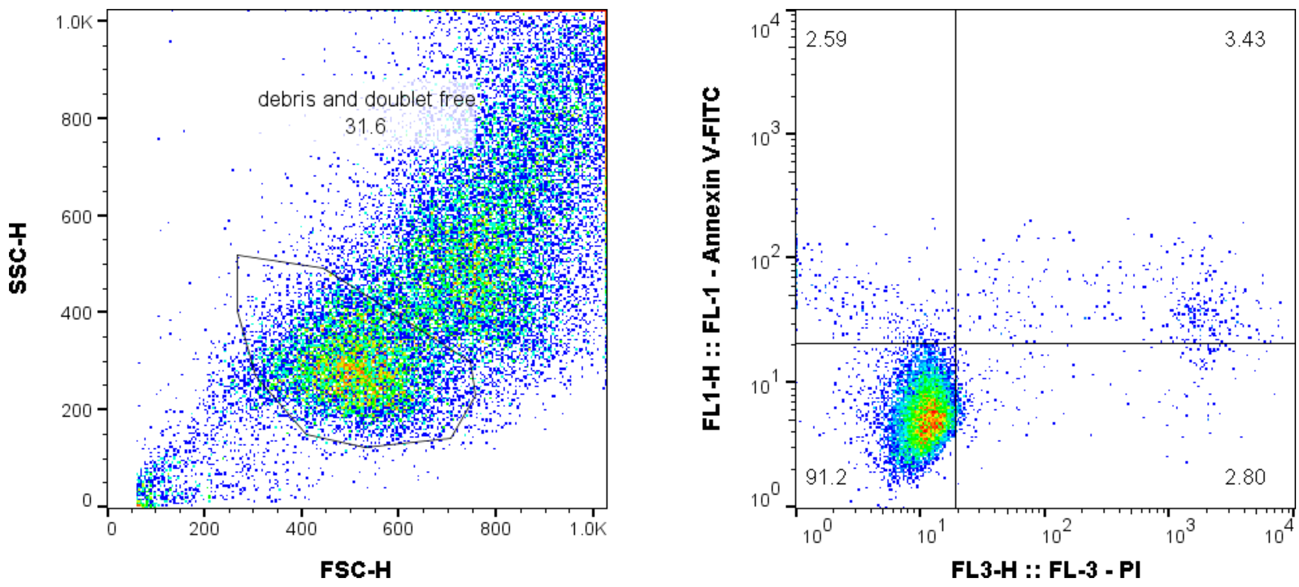

Figure S16. Phosphatidylserine externalization analysis gating strategy example (HT-29, hypoxia, 10 µM SKR, 48 h).

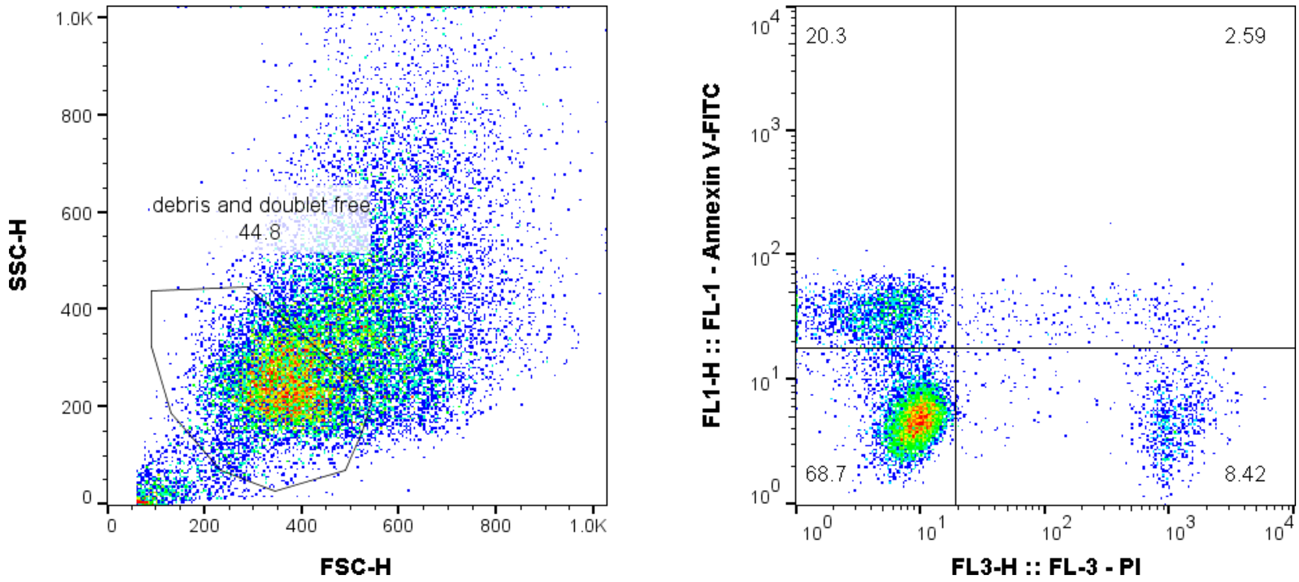

Figure S17. Phosphatidylserine externalization analysis gating strategy example (HT-29, hypoxia, 10 µM & 50 ng/mL TRAIL, 48 h).

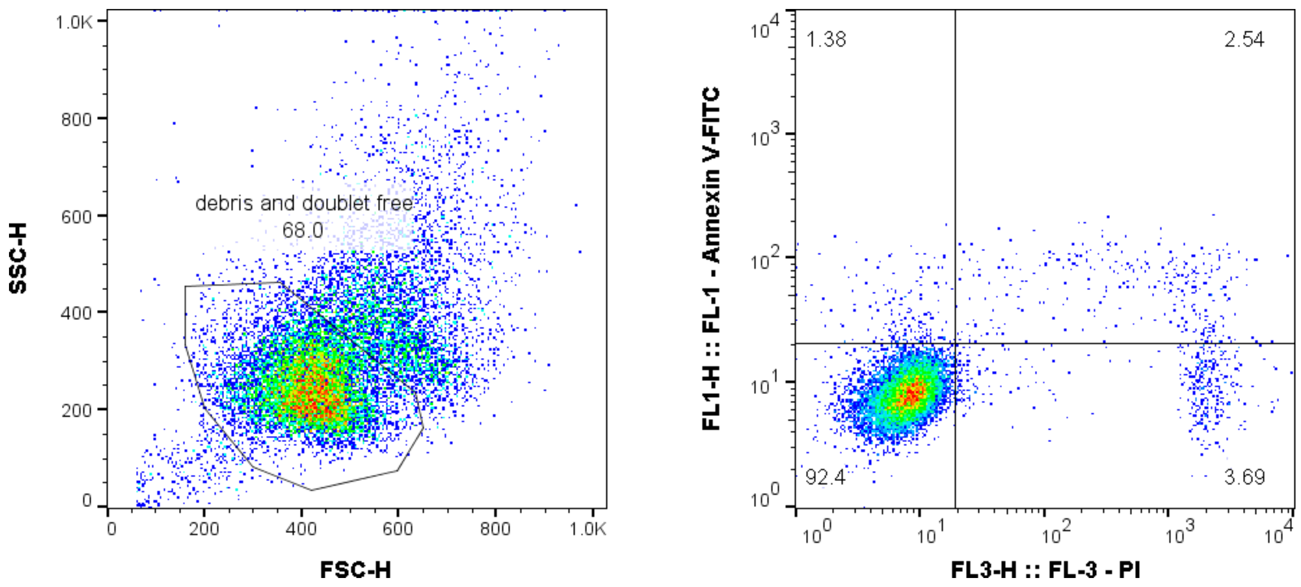

Figure S18. Phosphatidylserine externalization analysis gating strategy example (HT-29, normoxia, Control, 48 h).

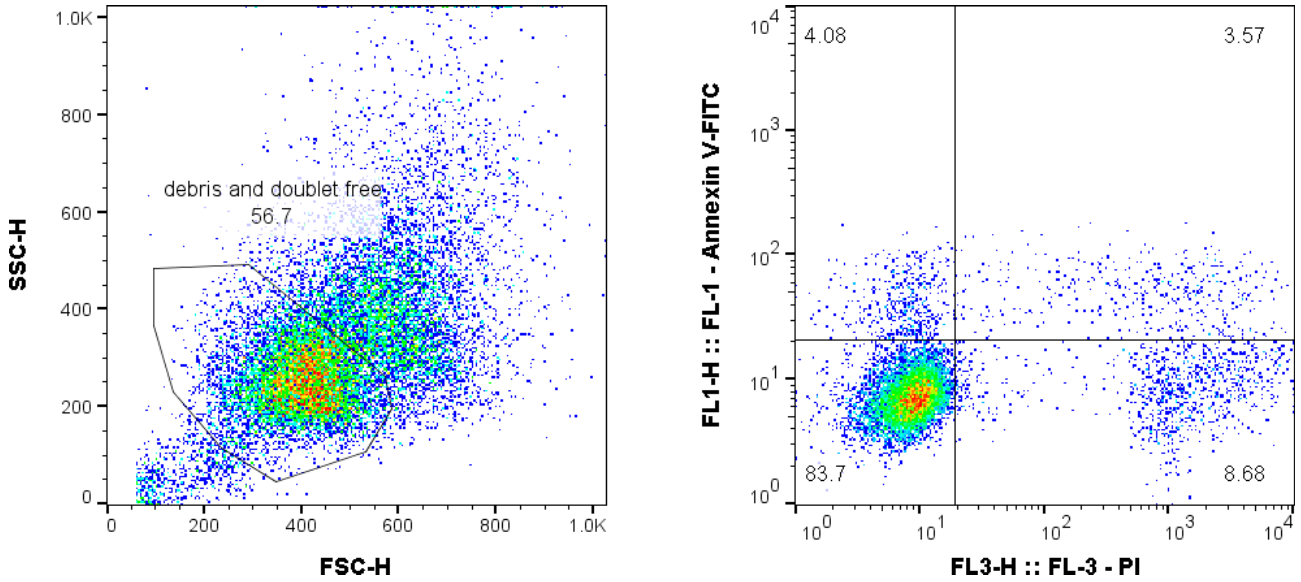

Figure S19. Phosphatidylserine externalization analysis gating strategy example (HT-29, normoxia, 50 ng/mL TRAIL, 48 h).

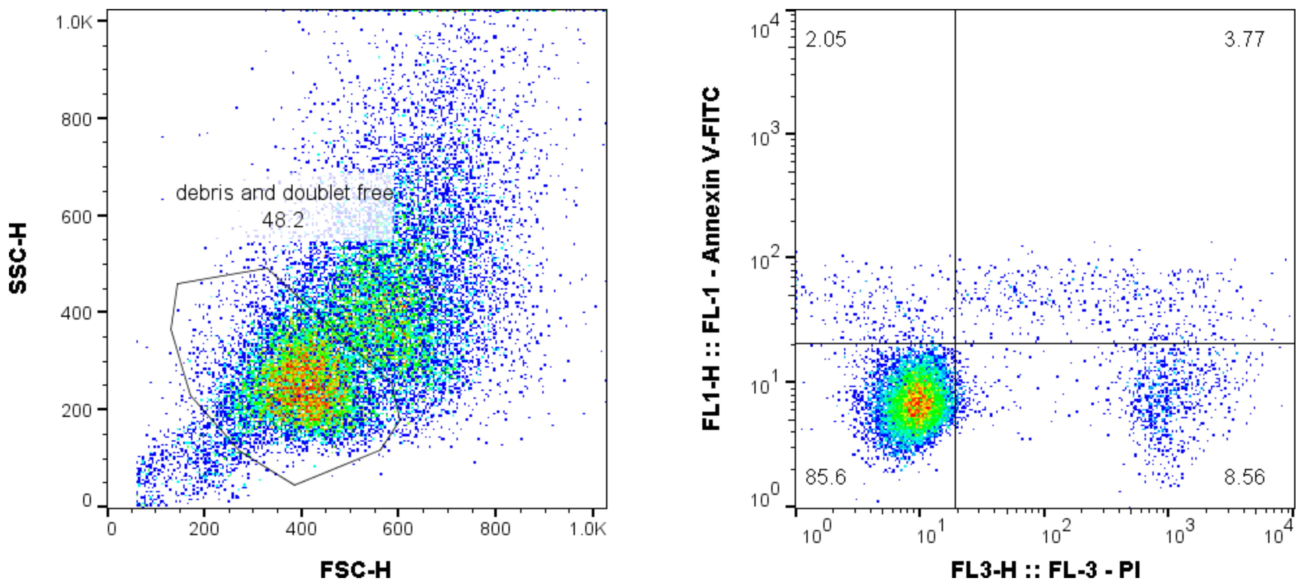

Figure S20. Phosphatidylserine externalization analysis gating strategy example (HT-29, normoxia, 10 µM SKR, 48 h).

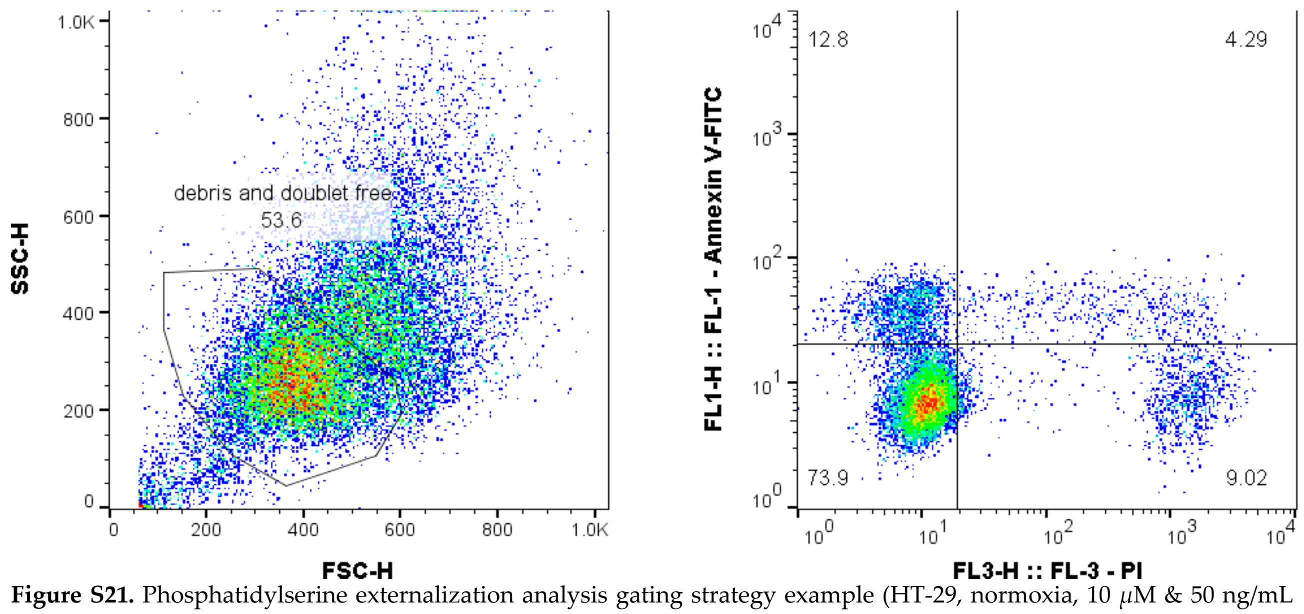

Figure S21. Phosphatidylserine externalization analysis gating strategy example (HT-29, normoxia, 10 µM & 50 ng/mL TRAIL, 48 h).

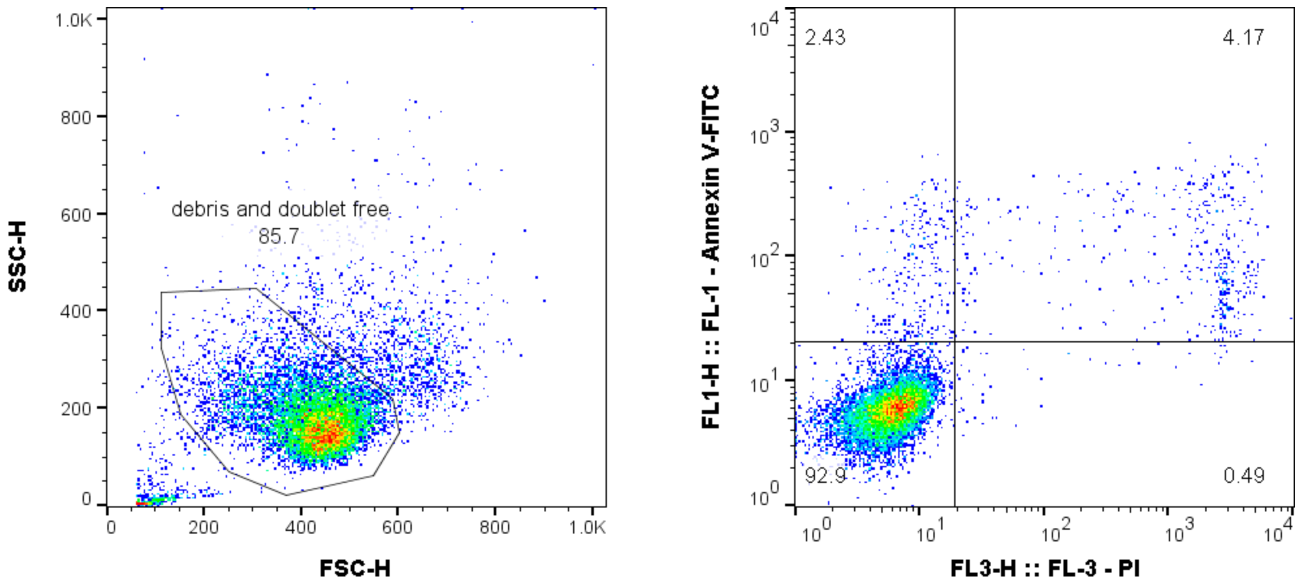

Figure S22. Phosphatidylserine externalization analysis gating strategy example (SW620, hypoxia, Control, 48 h).

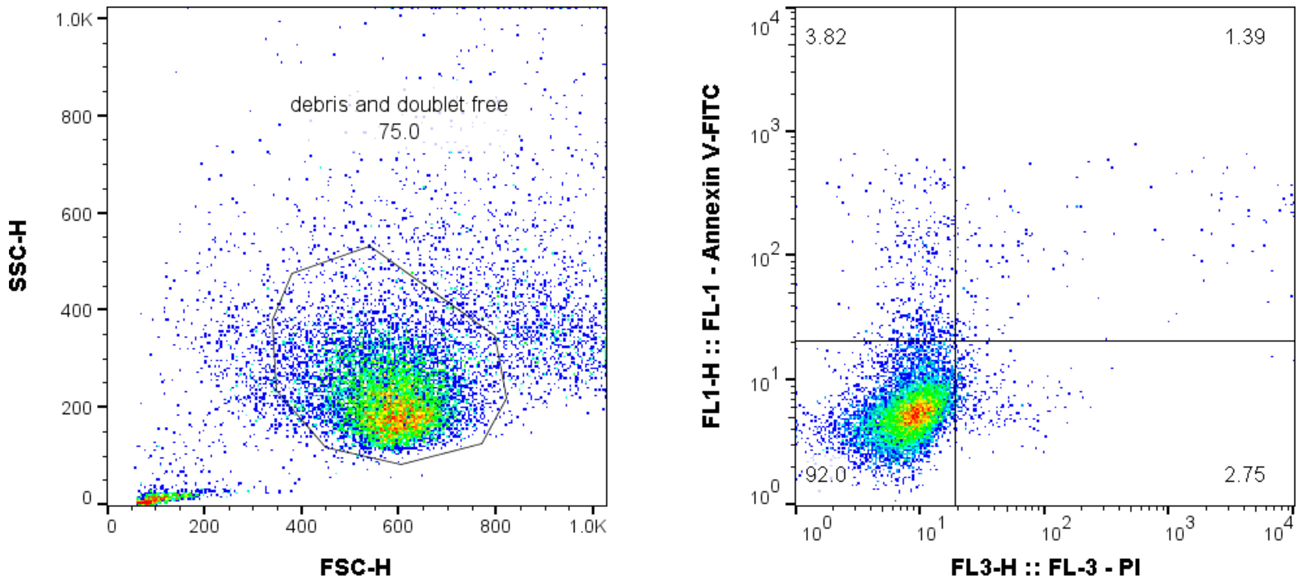

Figure S23. Phosphatidylserine externalization analysis gating strategy example (SW620, hypoxia, 50 ng/mL TRAIL, 48 h).

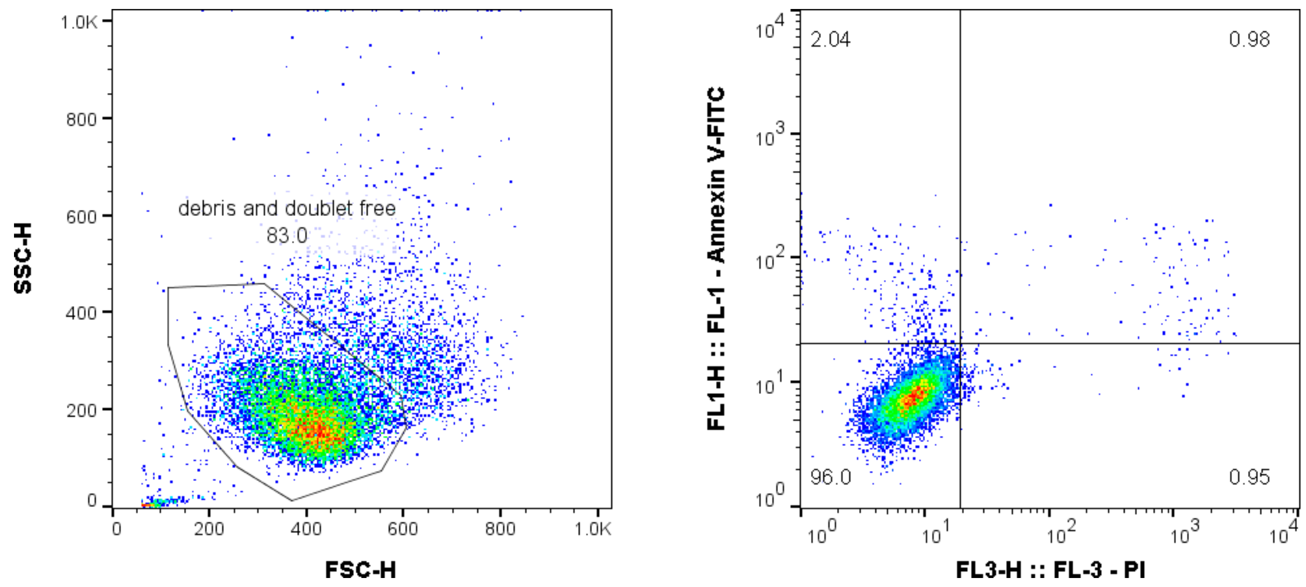

Figure S24. Phosphatidylserine externalization analysis gating strategy example (SW620, hypoxia, 10 µM SKR, 48 h).

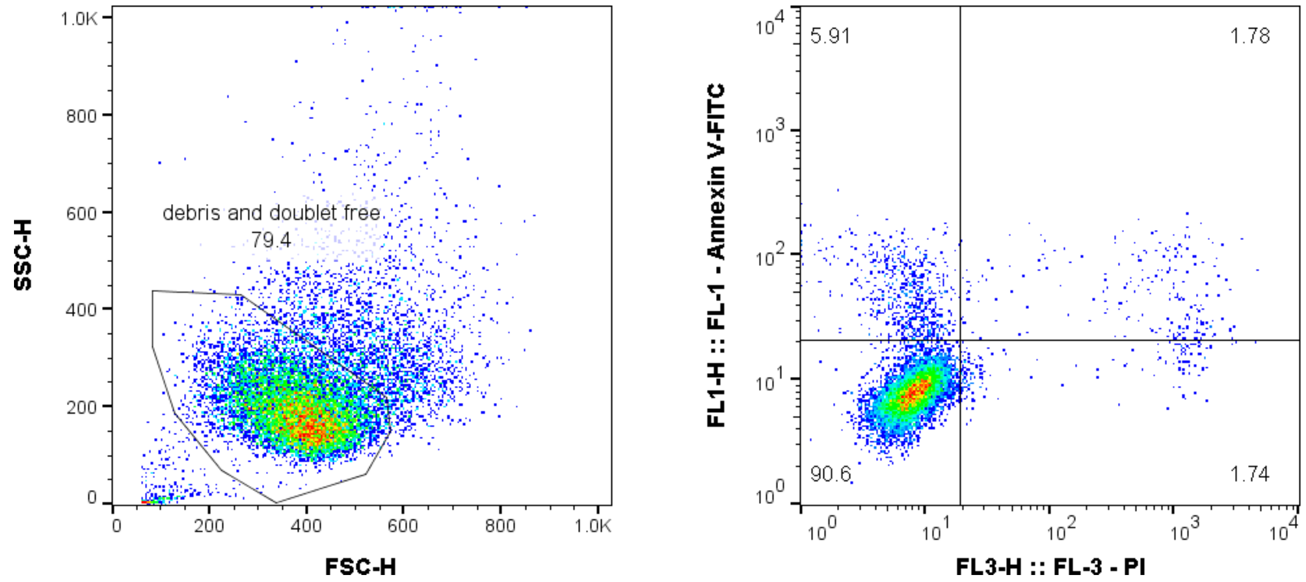

Figure S25. Phosphatidylserine externalization analysis gating strategy example (SW620, hypoxia, 10 µM & 50 ng/mL TRAIL, 48 h).

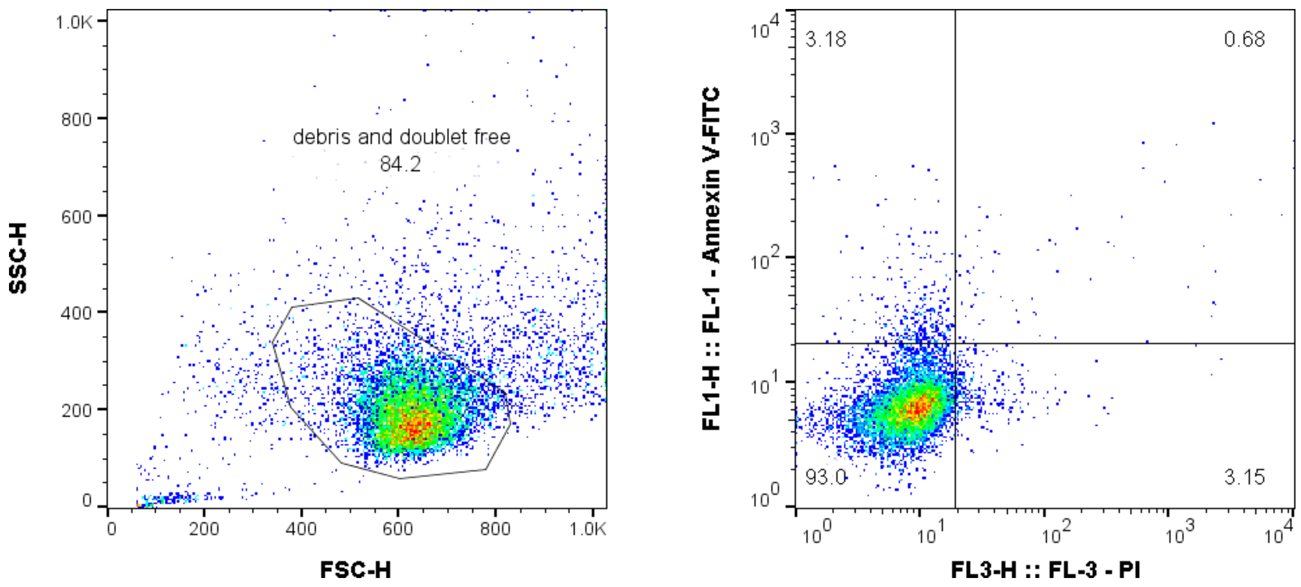

Figure S26. Phosphatidylserine externalization analysis gating strategy example (SW620, normoxia, Control, 48 h).

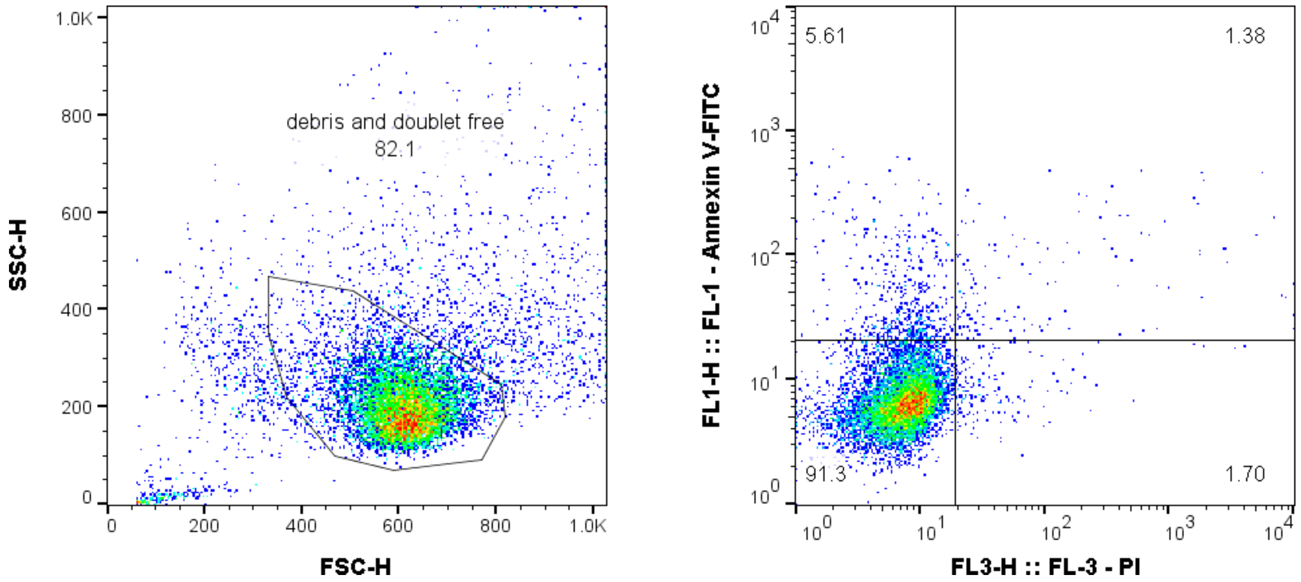

Figure S27. Phosphatidylserine externalization analysis gating strategy example (SW620, normoxia, 50 ng/mL TRAIL, 48 h).

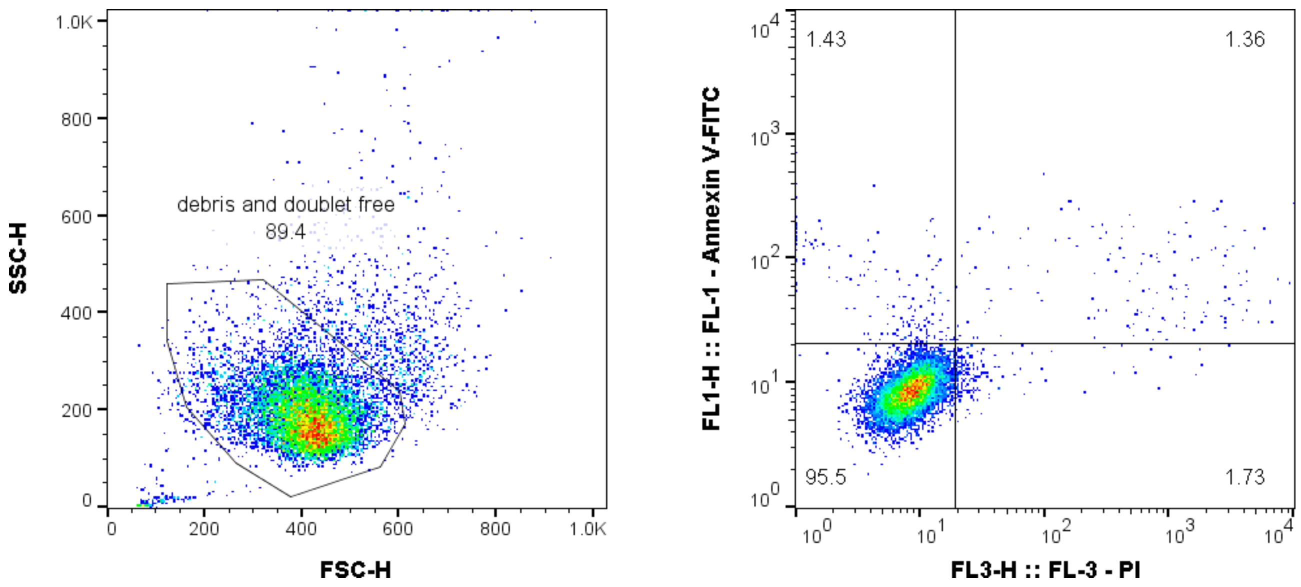

Figure S28. Phosphatidylserine externalization analysis gating strategy example (SW620, normoxia, 10 µM SKR, 48 h).

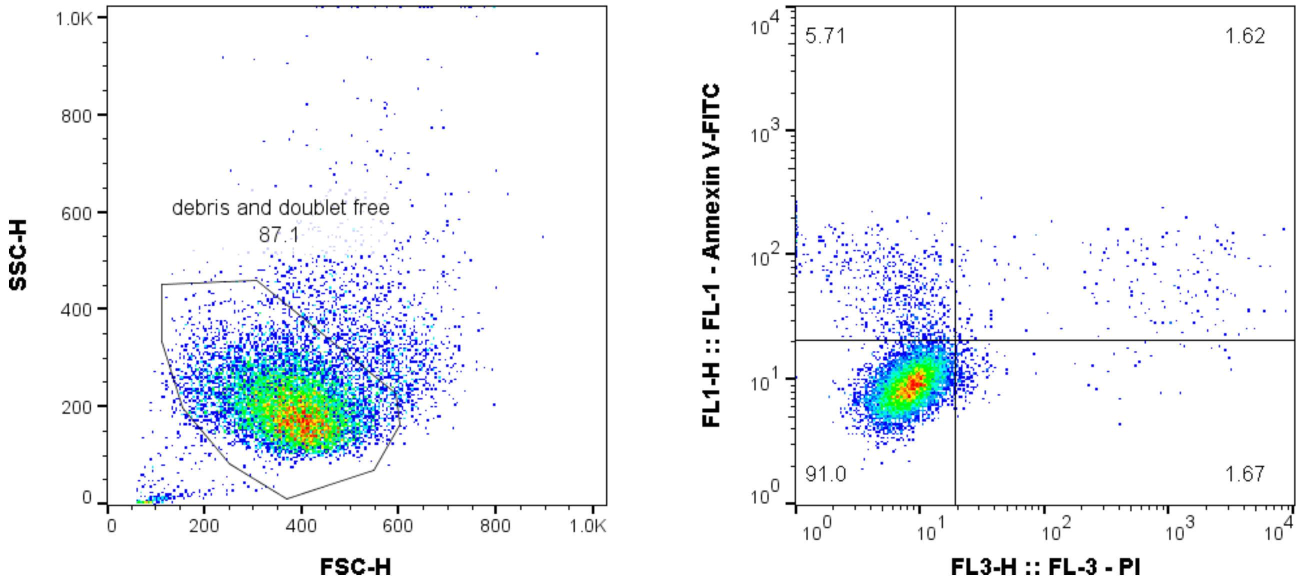

Figure S29. Phosphatidylserine externalization analysis gating strategy example (SW620, normoxia, 10 µM & 50 ng/mL TRAIL, 48 h).

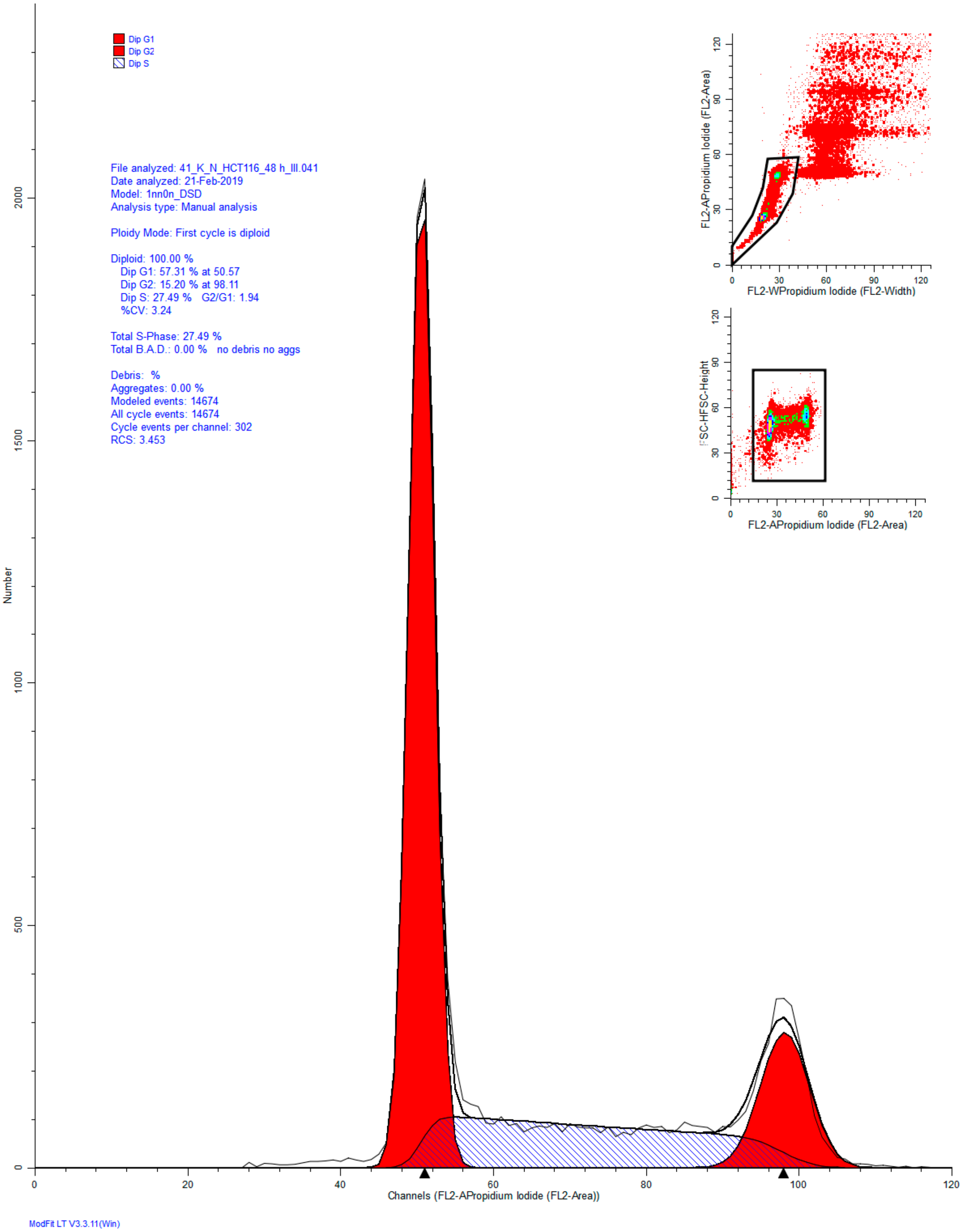

Figure S30. Cell cycle analysis gating strategy example (HCT 116, normoxia, Control, 48 h).

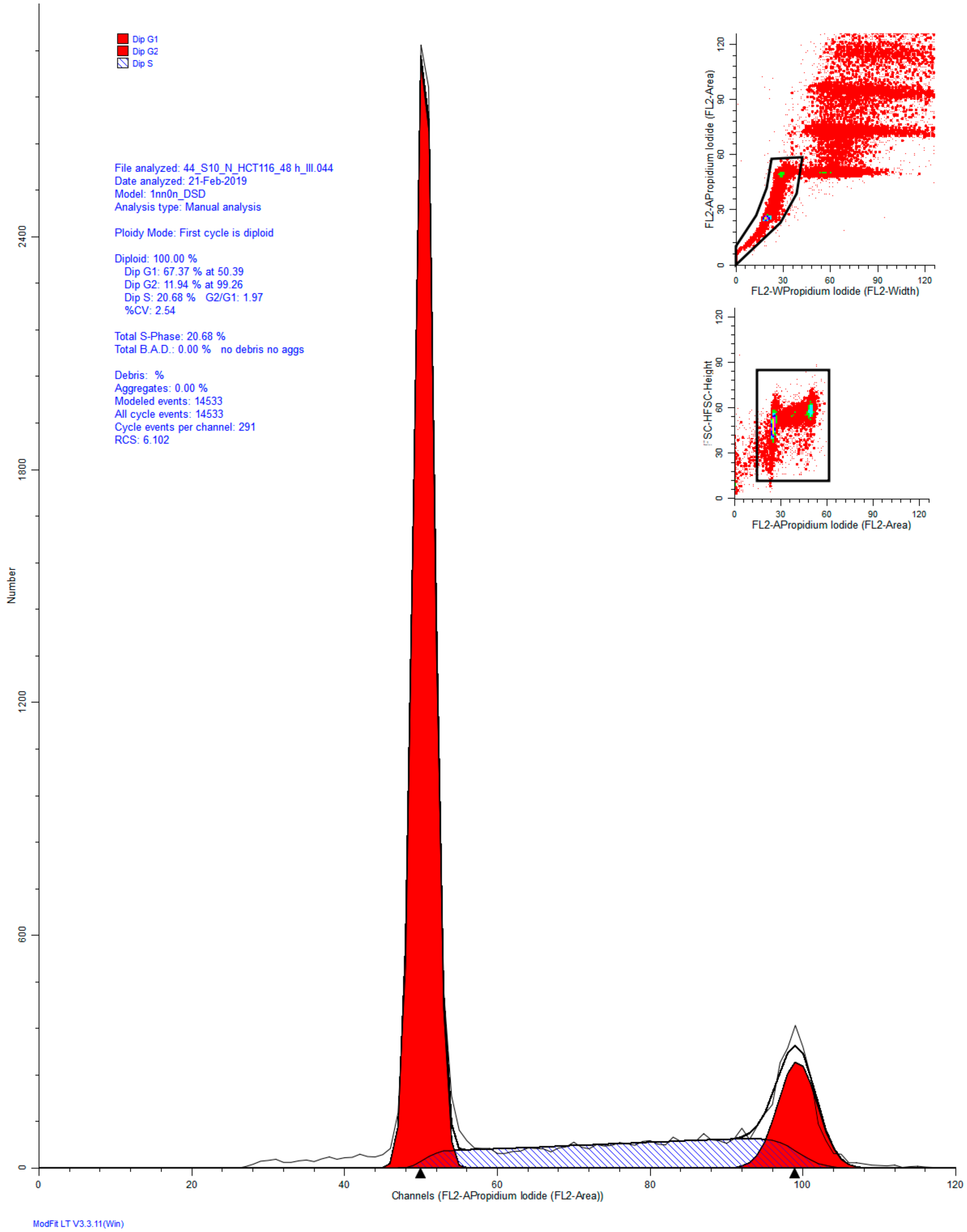

Figure S31. Cell cycle analysis gating strategy example HCT 116, normoxia, 10 µM SKR, 48 h.

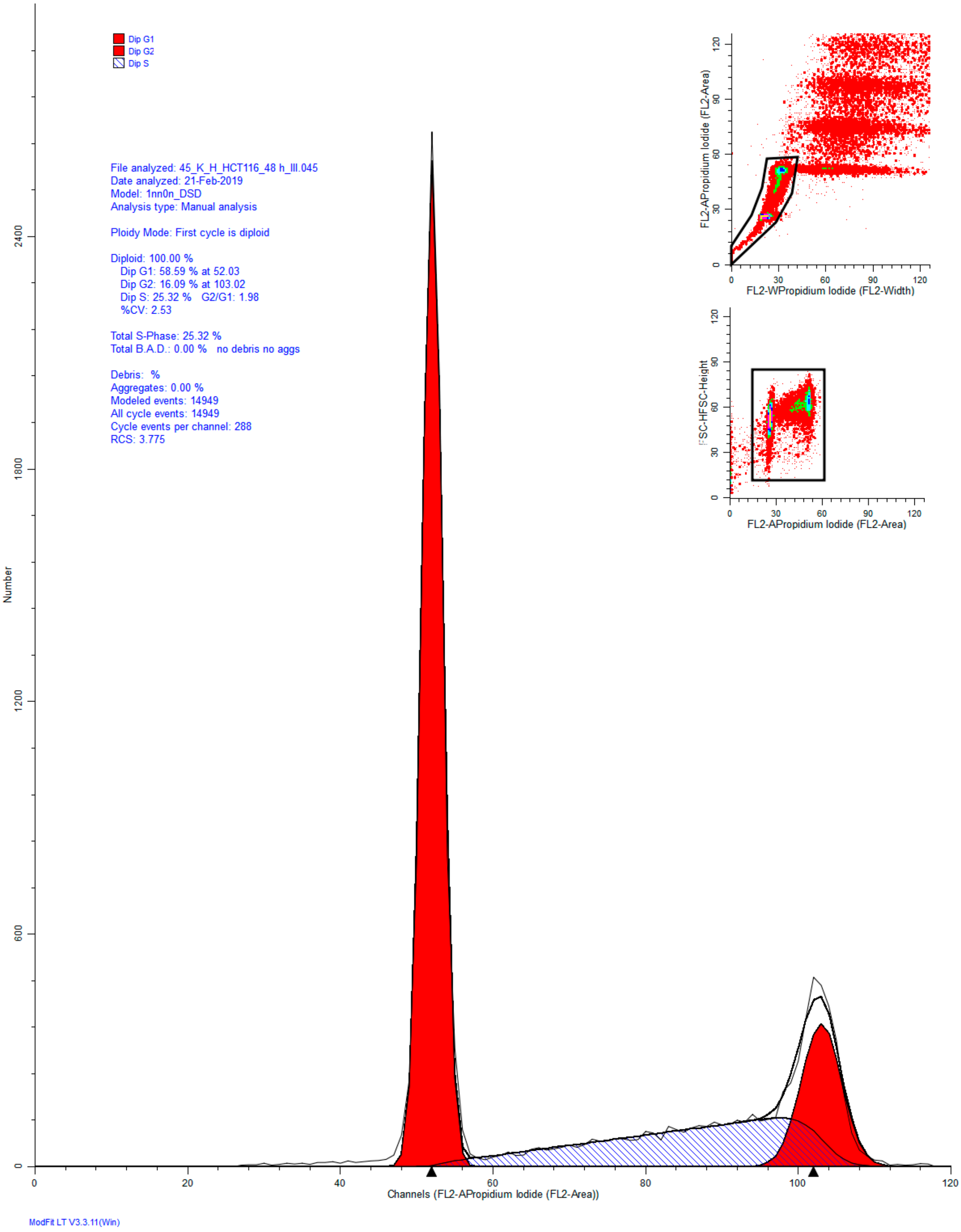

Figure S32. Cell cycle analysis gating strategy example HCT 116, hypoxia, Control, 48 h.

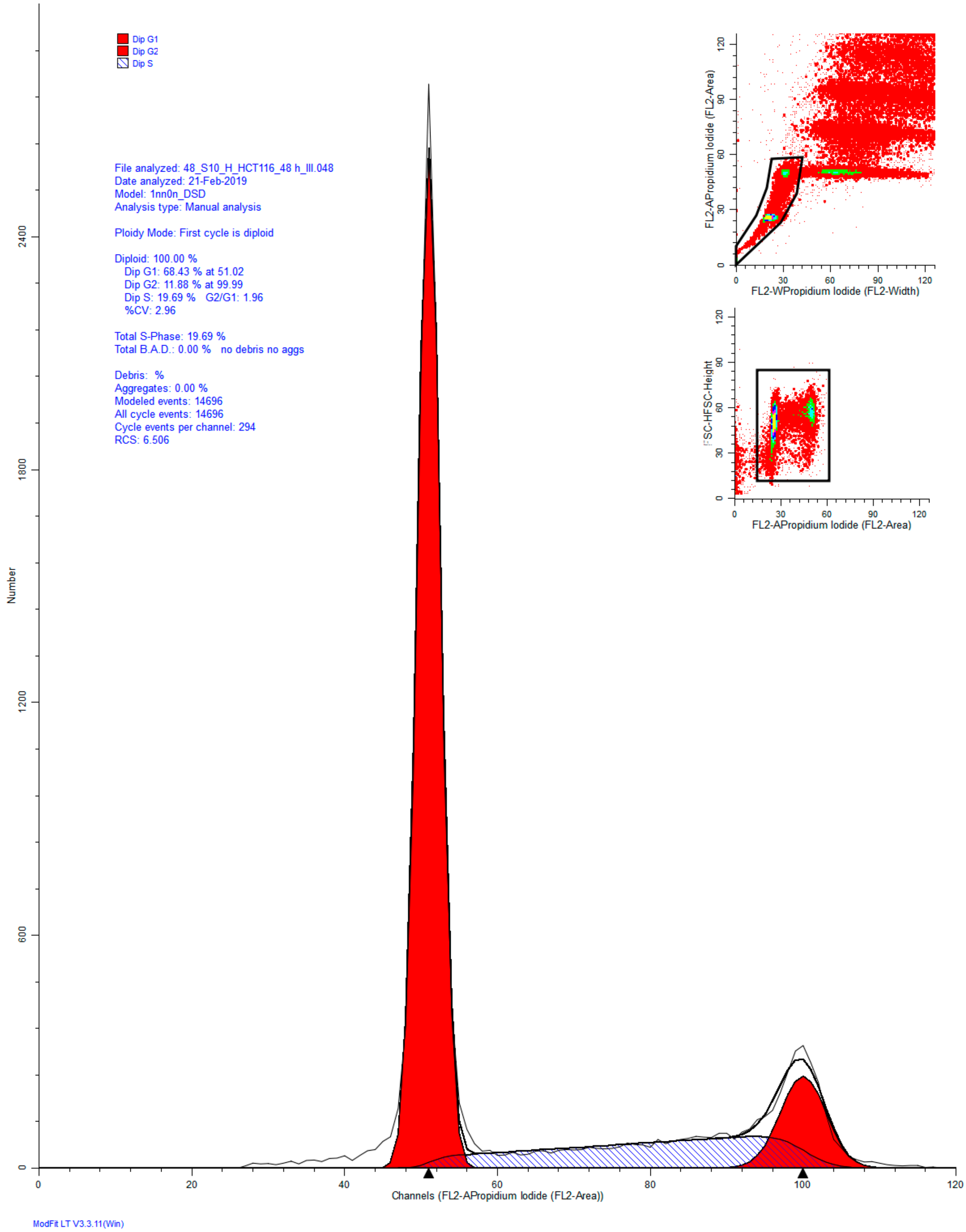

Figure S33. Cell cycle analysis gating strategy example HCT 116, hypoxia, 10 µM SKR, 48 h.

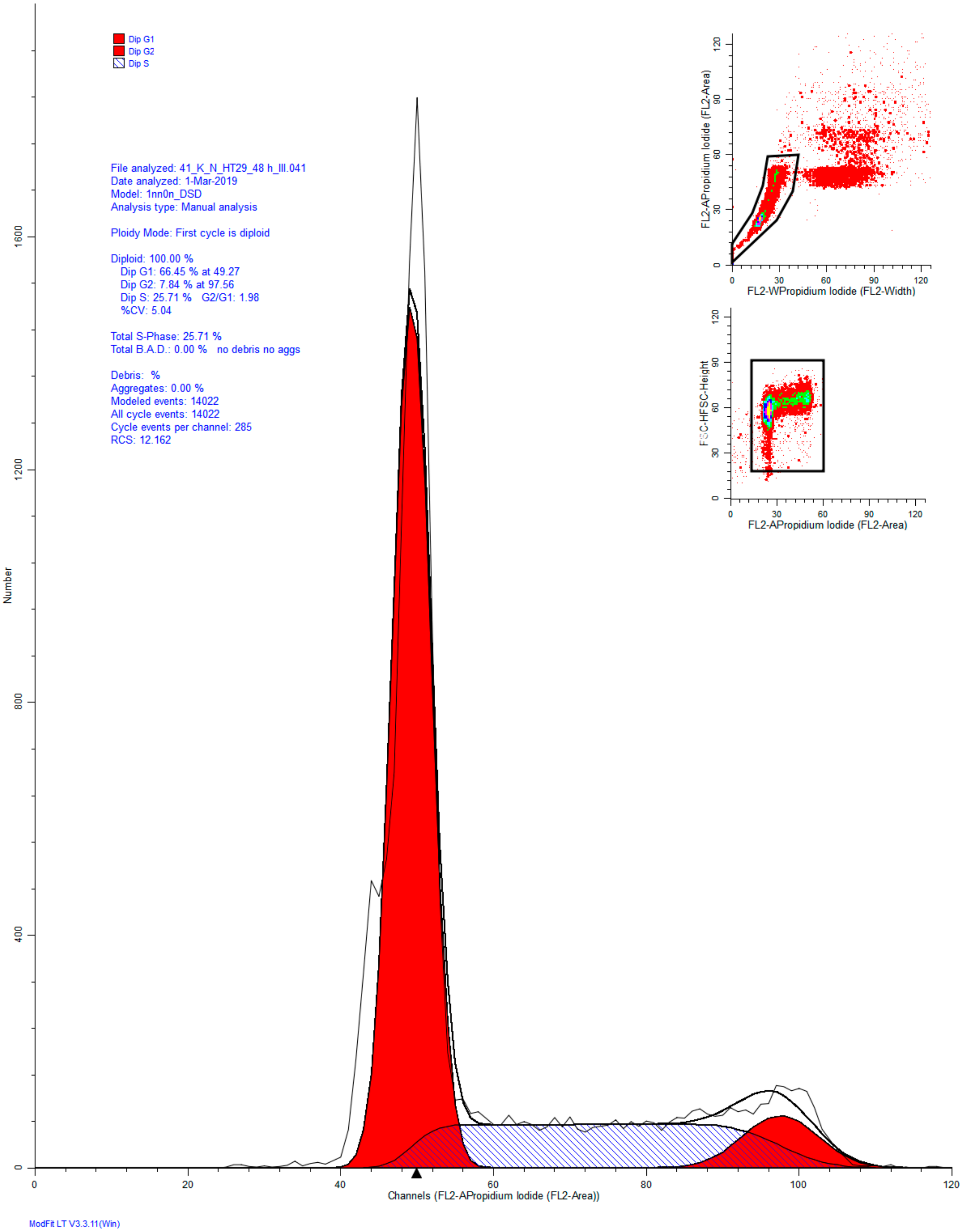

Figure S34. Cell cycle analysis gating strategy example HT-29, normoxia, Control, 48 h.

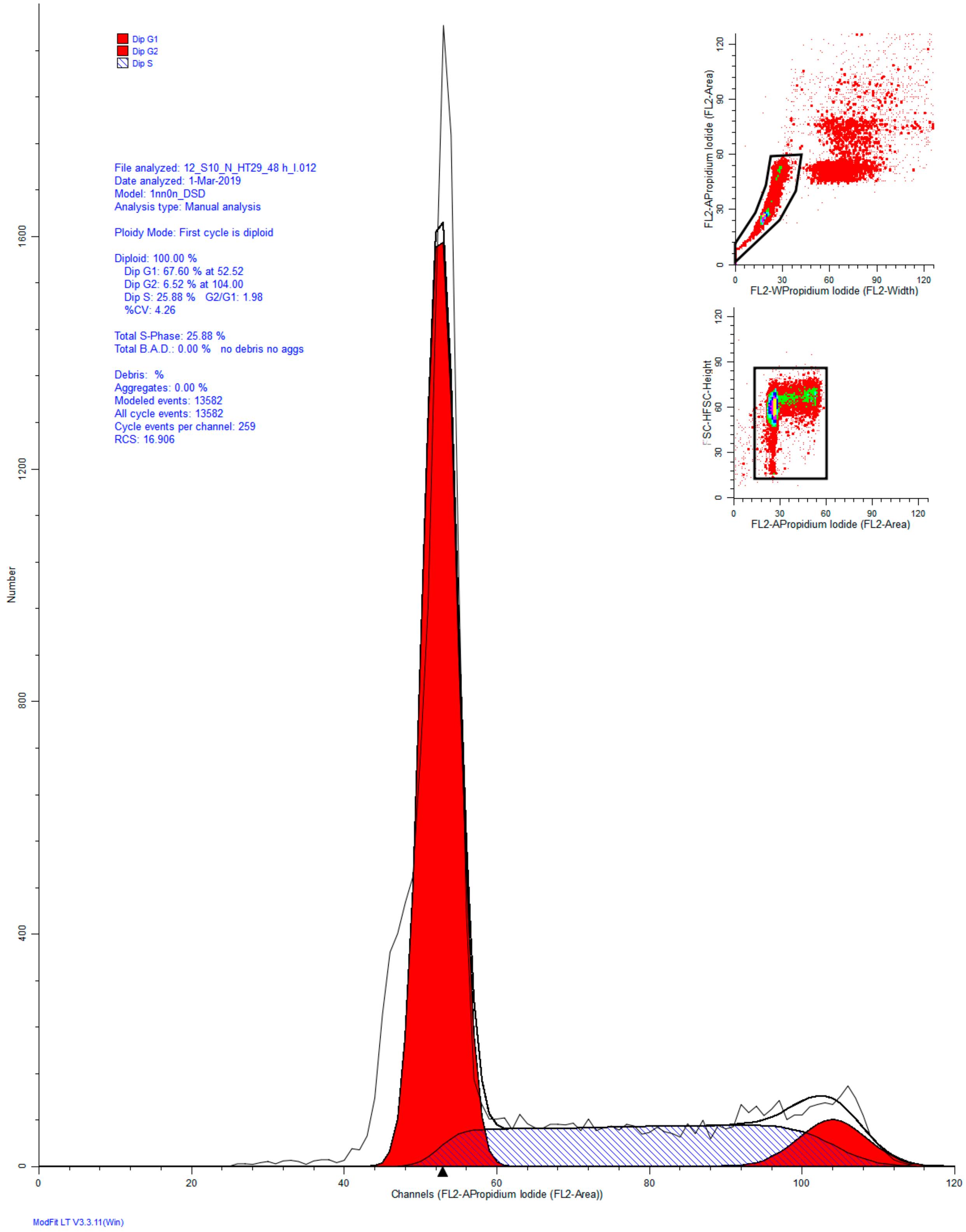

Figure S35. Cell cycle analysis gating strategy example HT-29, normoxia, 10 μM SKR, 48 h.

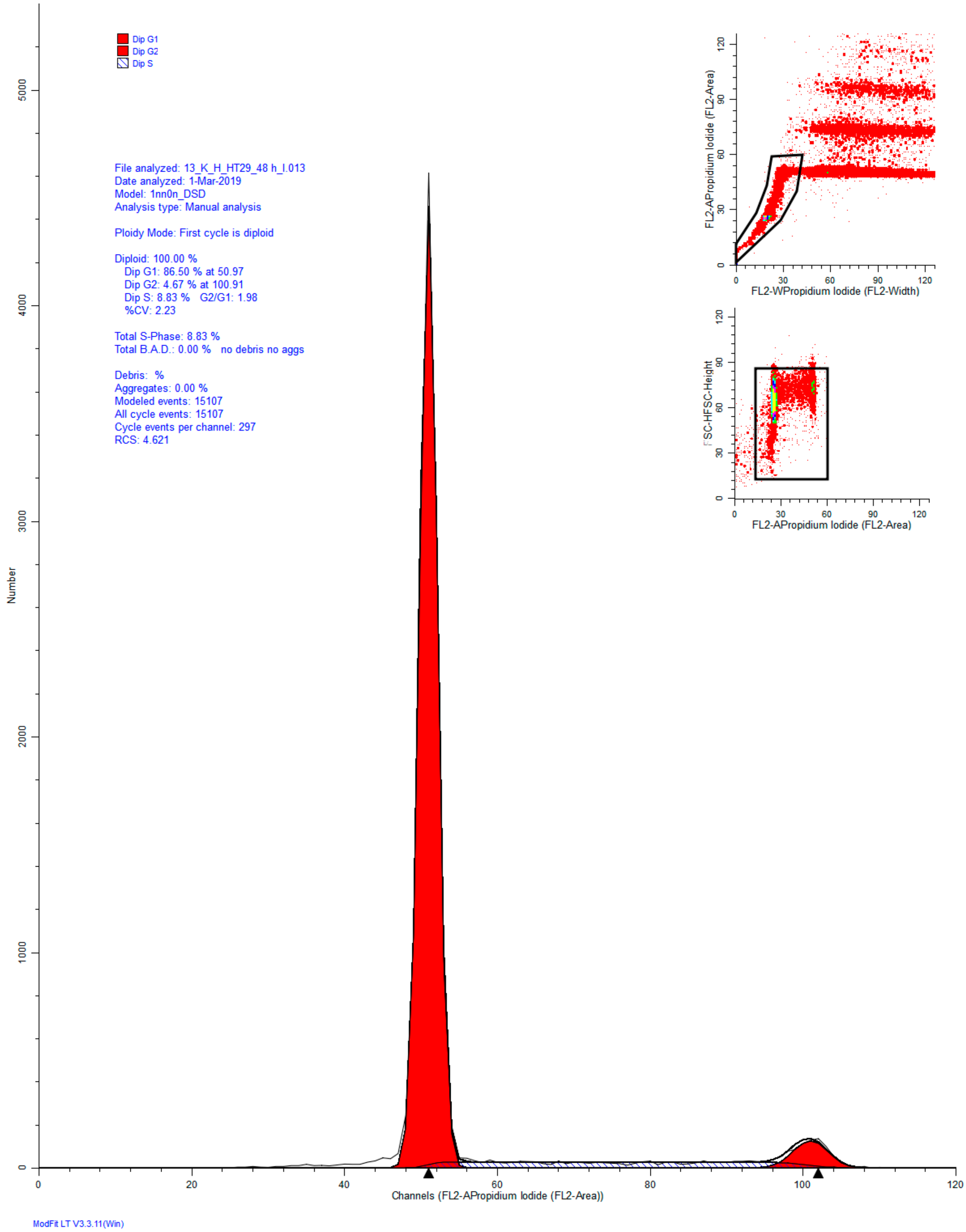

Figure S36. Cell cycle analysis gating strategy example HT-29, hypoxia, Control, 48 h.

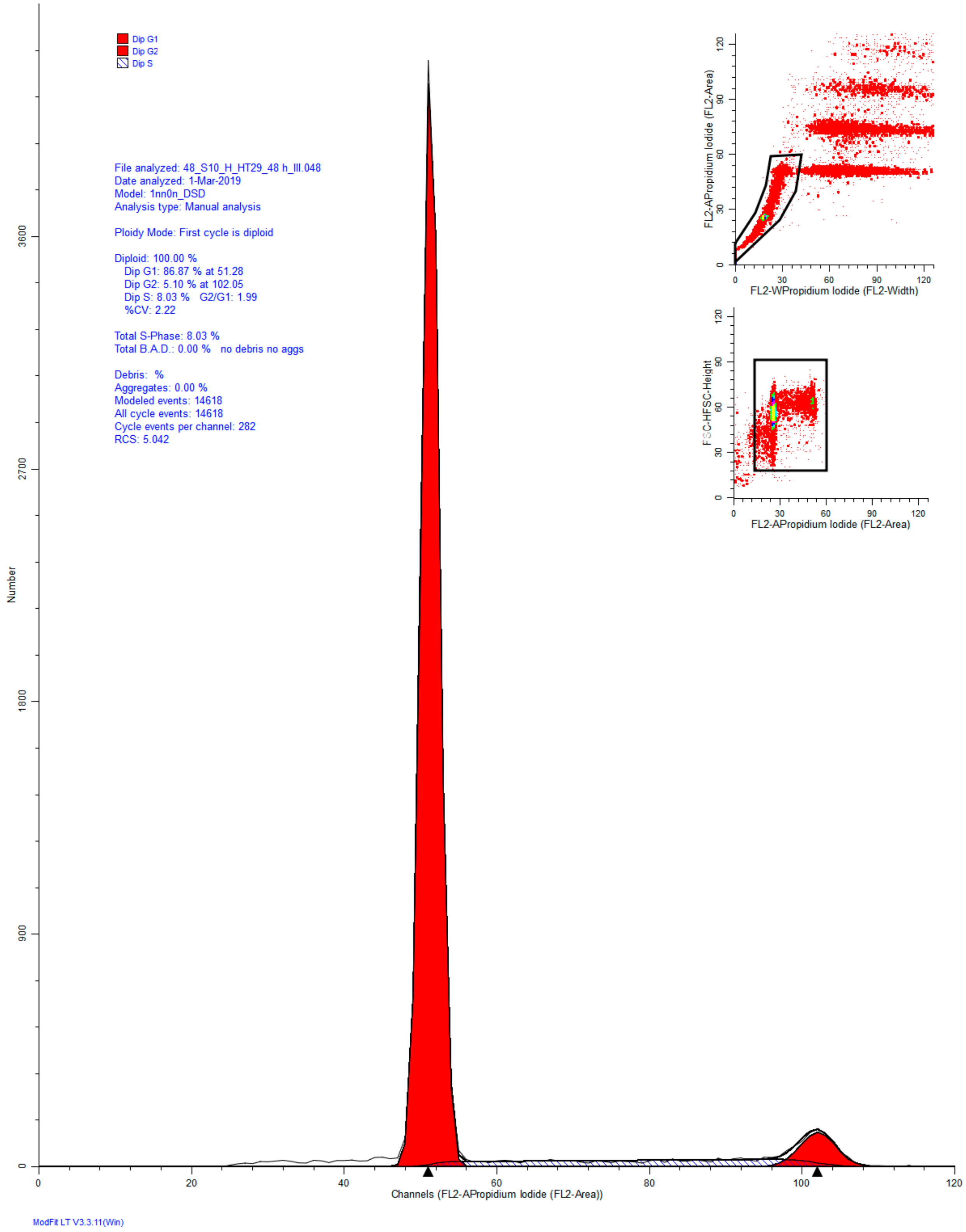

Figure S37. Cell cycle analysis gating strategy example HT-29, hypoxia, 10 µM SKR, 48 h.

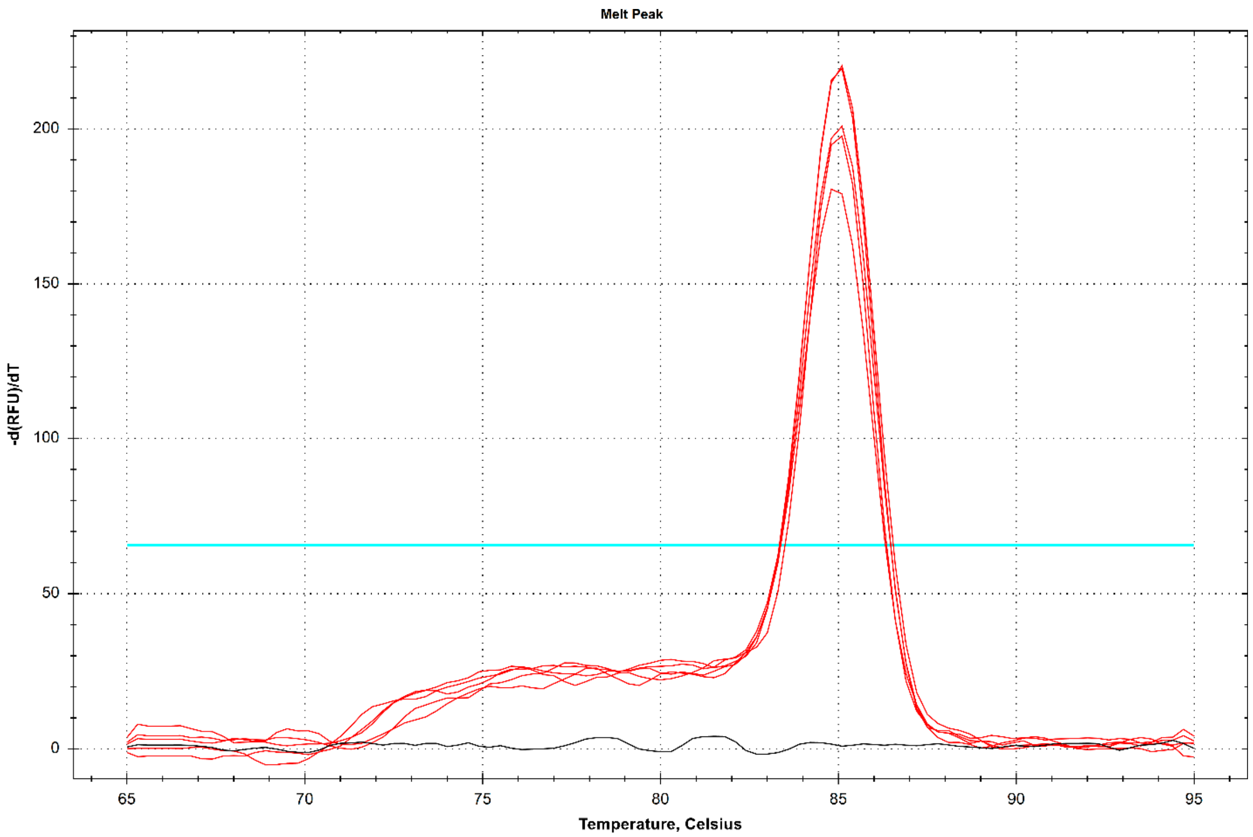

Figure S38. Melt curve of the *TNFRSF10B* (DR5—both isoforms) gene fragment amplified in RT-qPCR by oligonucleotide primers DR5_for and DR5_rev.

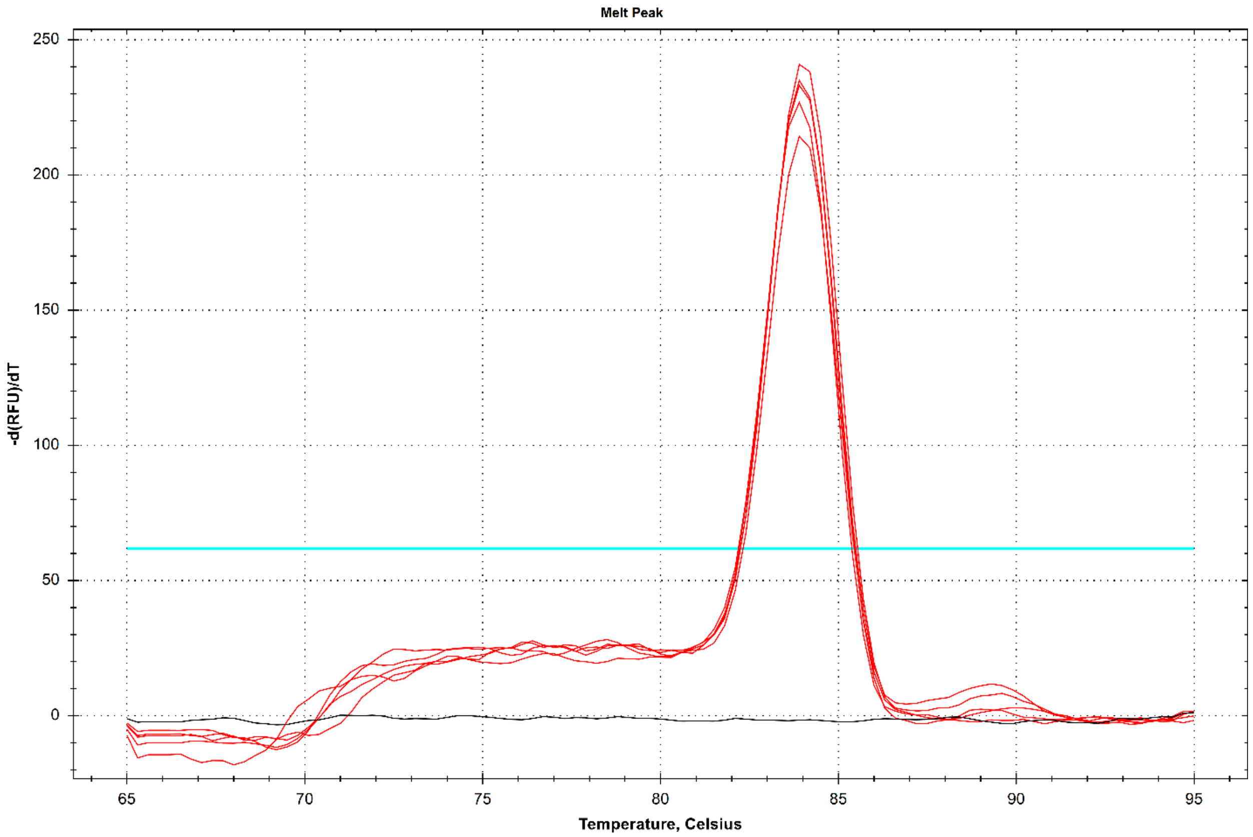

Figure S39. Melt curve of the *TNFRSF10B* (DR5—isoform 1) gene fragment amplified in RT-qPCR by oligonucleotide primers DR5.v1_for and DR5.v1_rev.

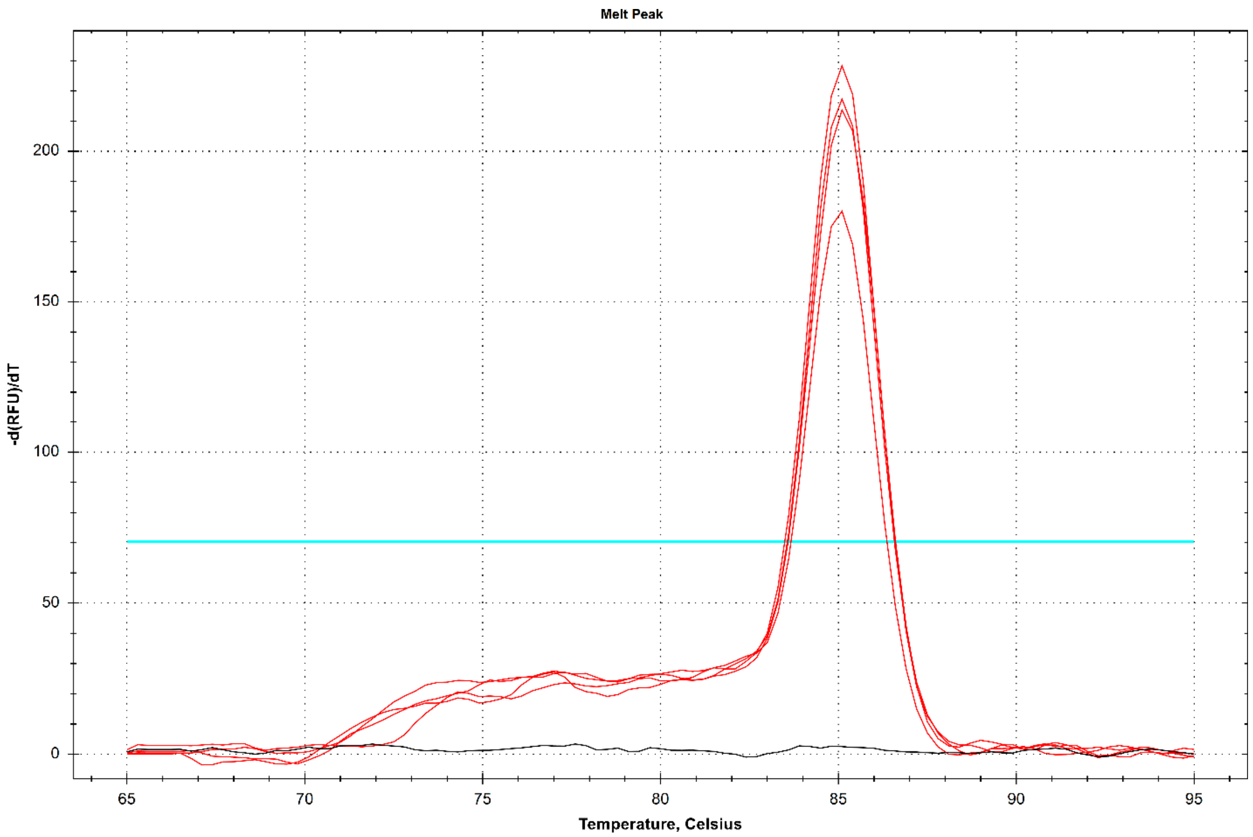

Figure S40. Melt curve of the *TNFRSF10B* (DR5—isoform 2) gene fragment amplified in RT-qPCR by oligonucleotide primers DR5.v2_for and DR5.v2_rev.

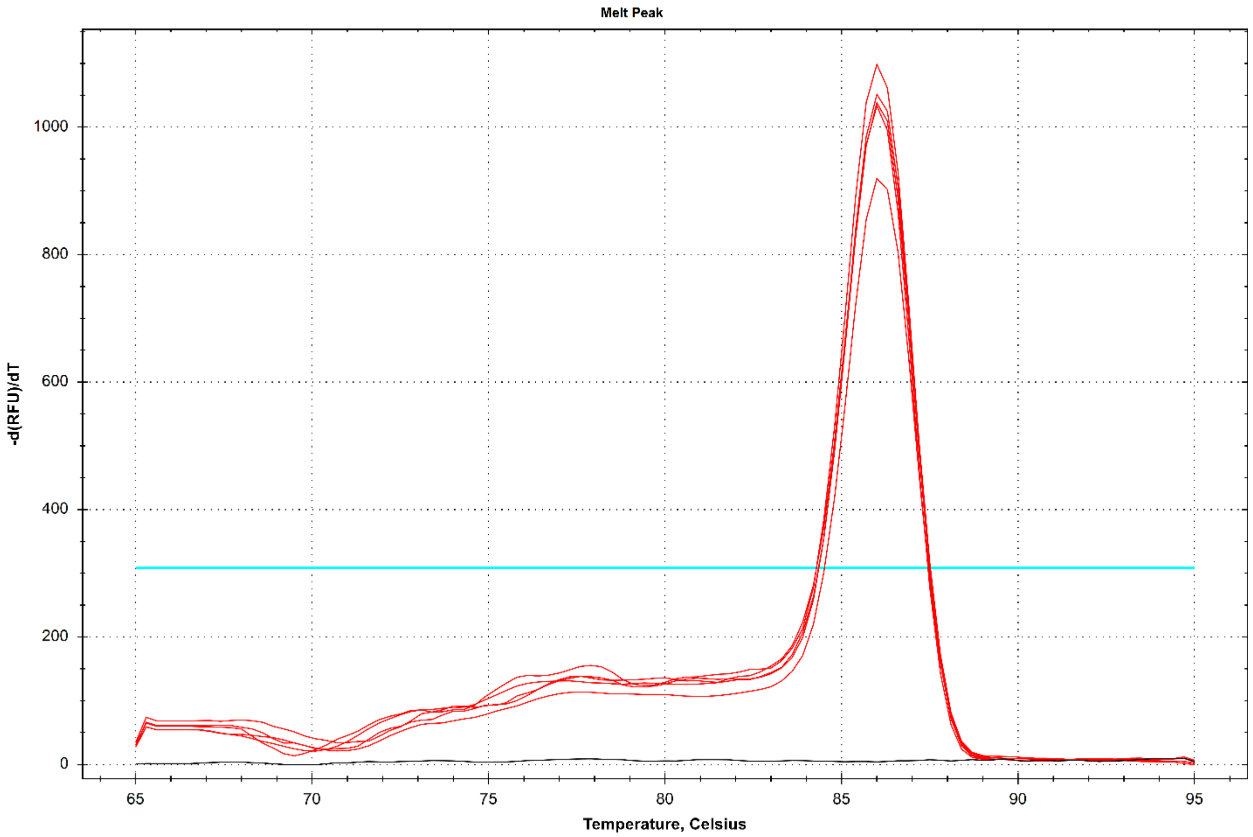

Figure S41. Melt curve of the *TNFRSF10A* (DR4) gene fragment amplified in RT-qPCR by oligonucleotide primers DR4_for and DR4_rev.

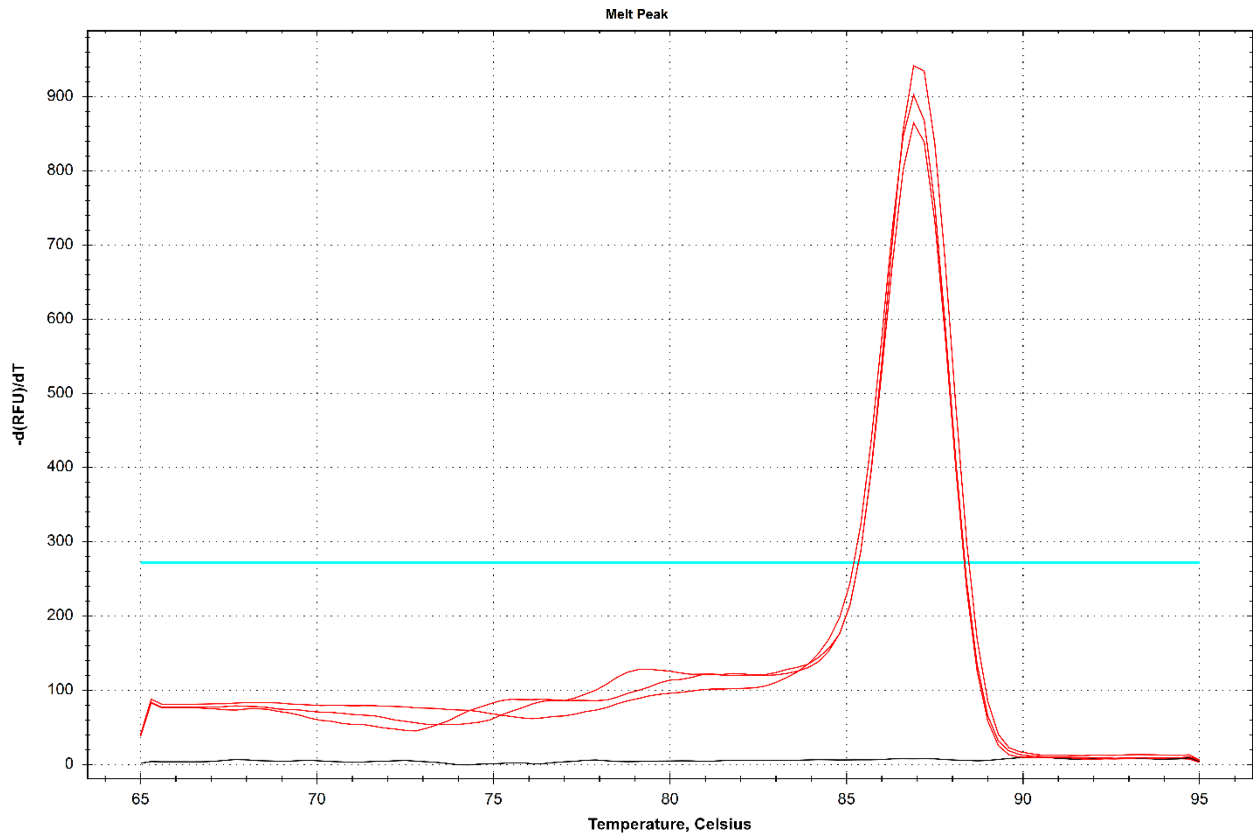

Figure S42. Melt curve of the *TNF* (TNF alpha) gene fragment amplified in RT-qPCR by oligonucleotide primers TNF_for and TNF_rev.

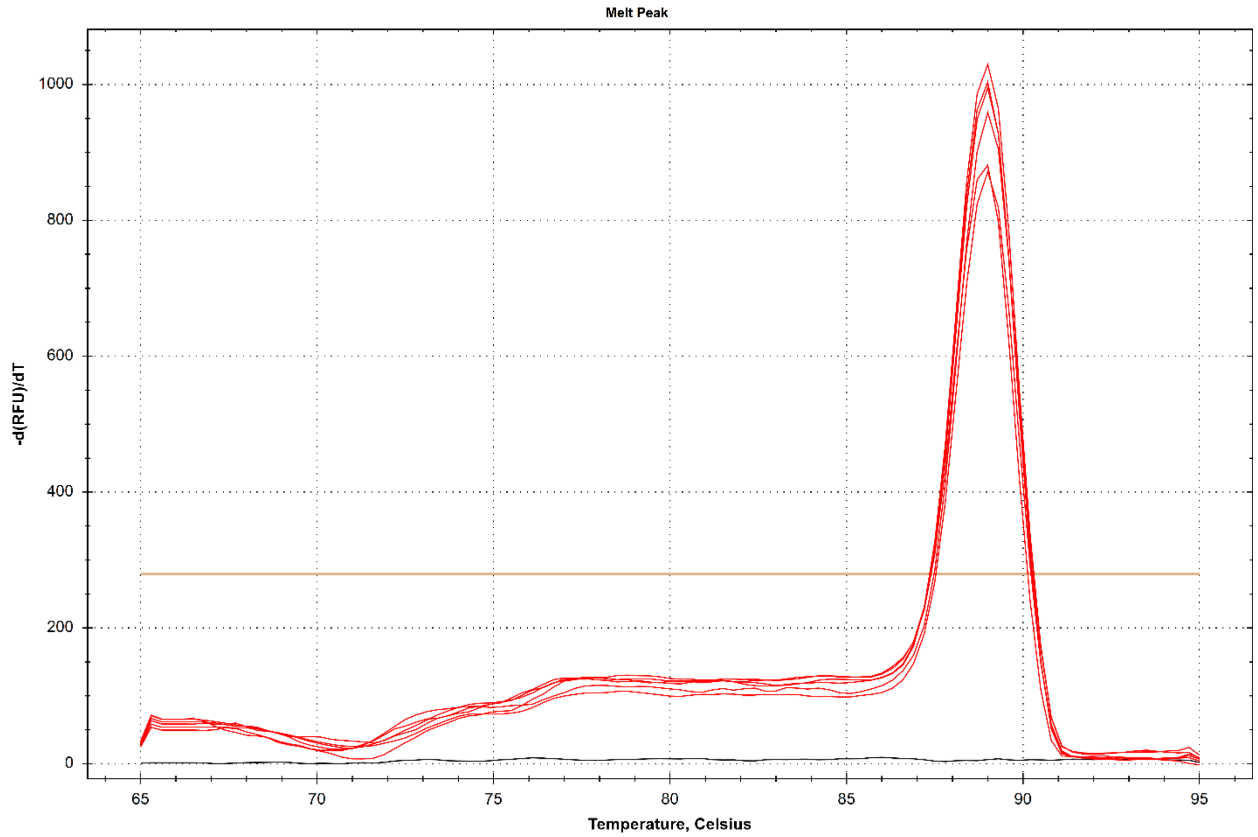

Figure S43. Melt curve of the *TNFRSF1A* (TNFR1) gene fragment amplified in RT-qPCR by oligonucleotide primers TNFRSF1A_for and TNFRSF1A _rev.

Figure S44. Melt curve of the *TNFRSF1B* (TNFR2) gene fragment amplified in RT-qPCR by oligonucleotide primers TNFRSF1B_for and TNFRSF1B_rev.

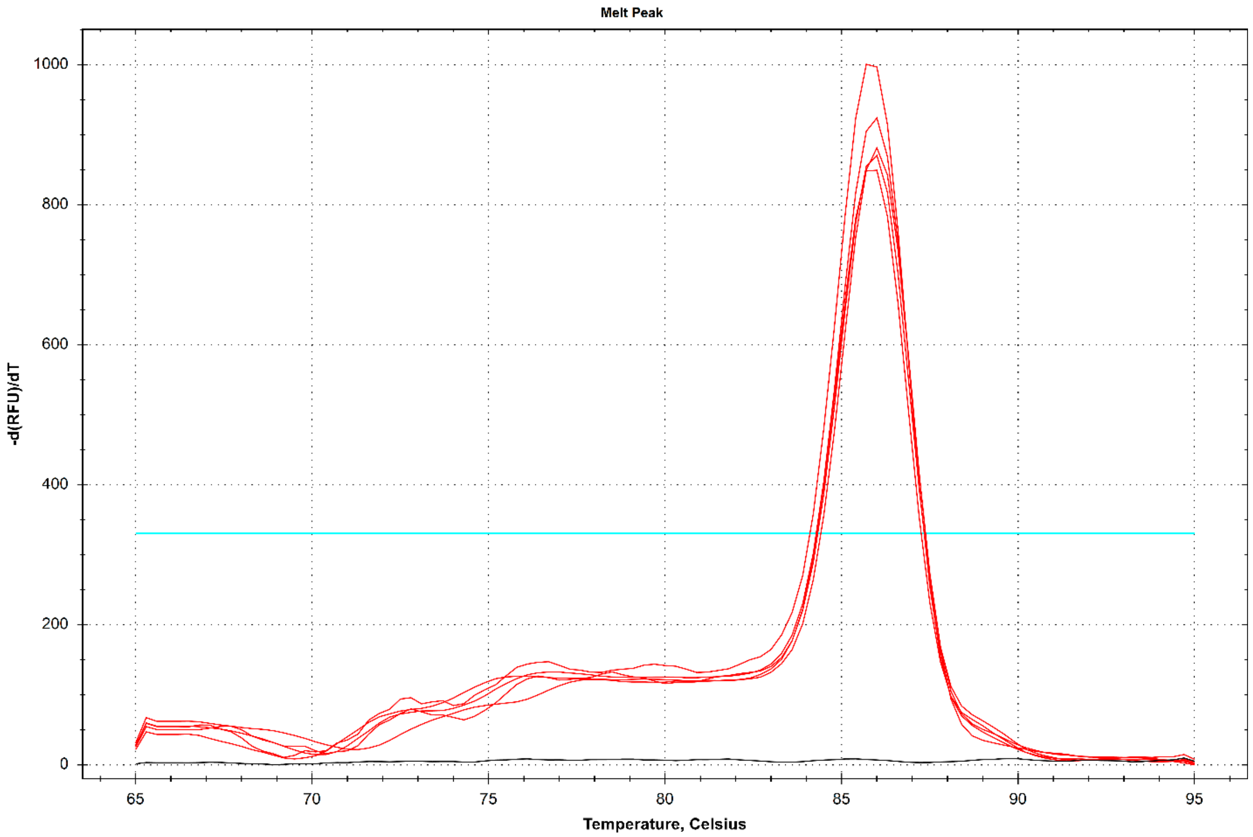

Figure S45. Melt curve of the *TNFRSF6B* (DcR3) gene fragment amplified in RT-qPCR by oligonucleotide primers DcR3_for and DcR3_rev.

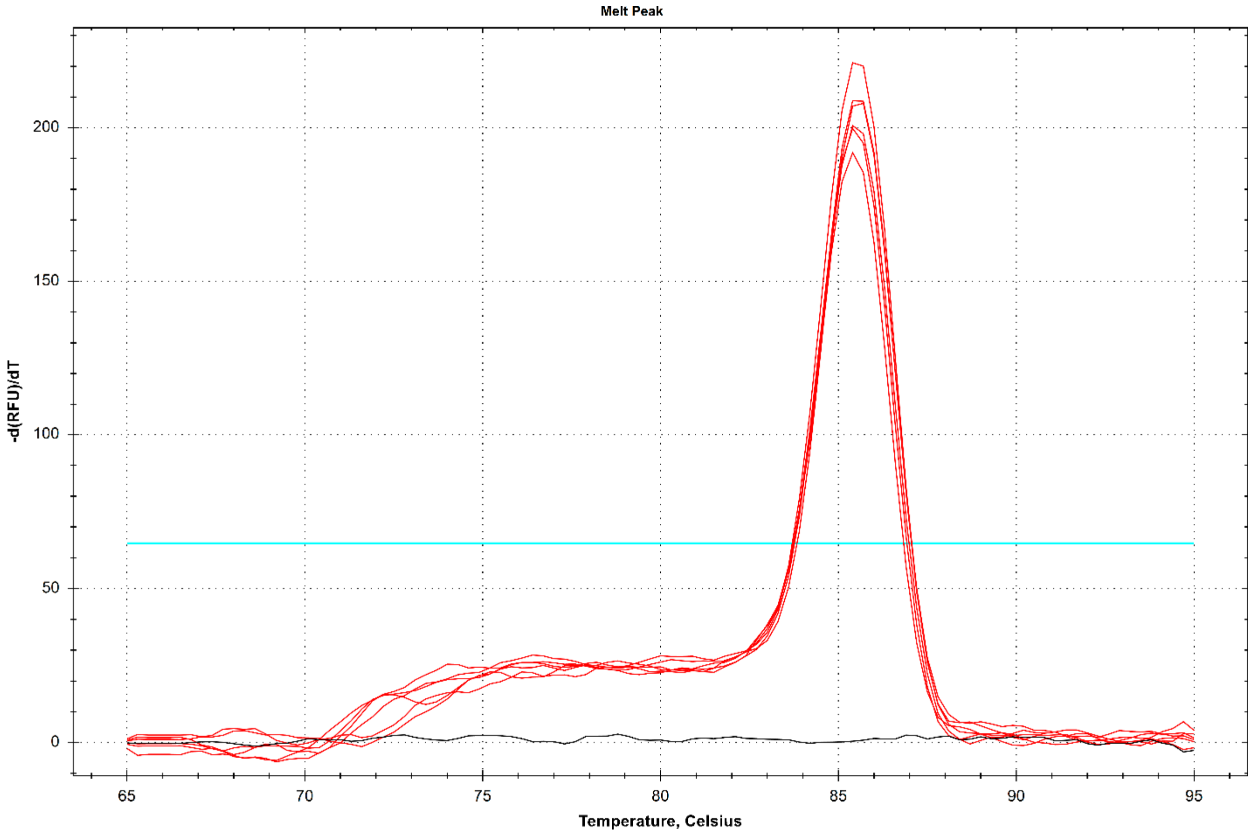

Figure S46. Melt curve of the *PMM1* gene fragment, reference gene, amplified in RT-qPCR by oligonucleotide primers PMM1_for and PMM1_rev.
